# 2-(Arylamino)thiazol-4(5H)-ones Mitigate Oxidative Stress and Confer Neuroprotection in Cellular and *Drosophila* Models of Alzheimer’s Disease

**DOI:** 10.3390/ijms27156642

**Published:** 2026-07-25

**Authors:** Nikhil Raj Selvaraj, Bhuvaneshwari S. V., Durga Nandan, Sandra San, Sudarslal Sadasivan Nair, Bipin G. Nair, Parvathy Venugopal, Rajaguru Aradhya, Vipin A. Nair

**Affiliations:** 1 School of Biotechnology, Amrita Vishwa Vidyapeetham, Kollam 690525, India; nikhilraj.ceib@gmail.com (N.R.S.); bhilae@gmail.com (D.N.); sandrasan@am.amrita.edu (S.S.); sudarslal@am.amrita.edu (S.S.N.); bipin@am.amrita.edu (B.G.N.); parvathyv@am.amrita.edu (P.V.); 2 Department of Chemistry, REVA University, Kattigenahalli, Yelahanka, Bangalore 560064, India; bhuvanashetty327@gmail.com

**Keywords:** thiazol, acetylcholinesterase inhibitors, Alzheimer’s disease, neuroprotection, oxidative stress, neurodegenerative diseases, *Drosophila*, SH-SY5Y cells

## Abstract

Alzheimer’s disease (AD) is a complex neurodegenerative disorder with limited effective therapies. In this study, a series of 2-(arylamino)thiazol-4(5H)-one derivatives was synthesized using an efficient microwave-assisted protocol, and evaluated for neuroprotective effects against 6-hydroxydopamine (6-OHDA)-induced oxidative stress (OS) in SH-SY5Y neuronal cells and an Aβ42-expressing *Drosophila melanogaster* model of AD. Among the synthesized compounds, **3n**, **3i**, and **3b** exhibited low cytotoxicity and significant neuroprotection, as evidenced by increased cell viability, reduced reactive oxygen species (ROS) production, and preserved mitochondrial membrane potential. Notably, compound **3n** demonstrated the highest activity and effectively ameliorated Aβ42-induced behavioral deficits in vivo. Molecular docking studies predicted favorable binding affinity within the acetylcholinesterase (AChE) active site, with the thiazol scaffold and aryl substituents stabilizing ligand binding through π–π stacking and hydrophobic interactions; compound **3n** also formed additional hydrogen bonds enhancing its affinity. Consistent with the docking predictions, in vitro AChE inhibition studies demonstrated that compounds **3n**, **3i**, and **3b** inhibited AChE in a concentration-dependent manner. Network pharmacology predicted seven potential core targets implicated in AD pathogenesis and related pathways, including OS, neuroinflammation, and synaptic dysfunction. Furthermore, in silico pharmacokinetic analysis indicated compliance with Lipinski’s and Veber’s rules, supporting favorable drug-like properties. While further experimental validation is essential, these findings highlight 2-(arylamino)thiazol-4(5H)-one derivatives, particularly compound **3n**, as promising multifunctional candidates for further preclinical development against AD.

## 1. Introduction

Neurodegenerative diseases (NDDs) encompass a diverse group of disorders marked by progressive neuronal dysfunction and cell death, resulting in cognitive decline, motor deficits, and ultimately death [1]. Alzheimer’s disease (AD) is the most prevalent neurodegenerative condition, currently affecting over 55 million individuals worldwide, with estimates projecting this figure to triple by 2050 [2]. The underlying pathophysiology of NDDs involves complex mechanisms including protein misfolding and aggregation, oxidative stress (OS), mitochondrial dysfunction, neuroinflammation and excitotoxicity [3]. This multifactorial nature has complicated drug development, with numerous single-target approaches failing to demonstrate disease-modifying effects in clinical trials.

Among these mechanisms implicated in AD progression, OS has emerged as a central contributor to neuronal death [4]. Elevated levels of reactive oxygen species (ROS) trigger lipid peroxidation, protein oxidation, and DNA damage, ultimately leading to neuronal death and synaptic dysfunction, the hallmarks of AD pathology [5,6]. Importantly, oxidative injury in AD is not merely a downstream consequence but may serve as an early pathogenic event that amplifies amyloid-beta (Aβ) toxicity [7]. The oligomeric Aβ species can insert into membrane systems, promoting OS that damages key cellular components involved in energy metabolism, signaling, neurotransmission, and protein degradation pathways [8]. This oxidative environment exacerbates protein aggregation, impairs synaptic function, and accelerates neurodegeneration. Thus, small molecules capable of reducing OS while preserving mitochondrial function represent promising candidates for disease-modifying intervention in AD.

In parallel with OS-centered therapeutic strategies, the cholinergic hypothesis continues to provide a major framework for anti-Alzheimer drug discovery [9]. Acetylcholinesterase (AChE), beyond its classical role in hydrolyzing acetylcholine and regulating cholinergic neurotransmission, has been implicated in broader pathological processes relevant to neurodegeneration, including neuroinflammation, apoptosis, oxidative injury, and Aβ aggregation [10]. Particularly notable is the role of AChE in accelerating amyloid fibrillogenesis through interactions at its peripheral anionic site, linking cholinergic dysfunction with amyloid pathology [11,12]. This multifunctional role has made AChE an enduring therapeutic target, as reflected by clinically used inhibitors such as Donepezil, Rivastigmine, and Galantamine, which provide symptomatic benefit through enhanced cholinergic transmission [13,14,15].

Importantly, newer generations of AChE inhibitors have moved beyond simple catalytic inhibition toward multifunctional ligand design [16]. For example, galantamine acts as an allosteric potentiator of neuronal nicotinic receptors and activates antiapoptotic pathways like Bcl-2 upregulation, supporting neuronal survival [17,18]. Some of these compounds additionally target butyrylcholinesterase and β-secretase, reflecting a broader multi-target-directed ligand strategy increasingly recognized as necessary for complex disorders such as AD [19].

**Figure 1:**
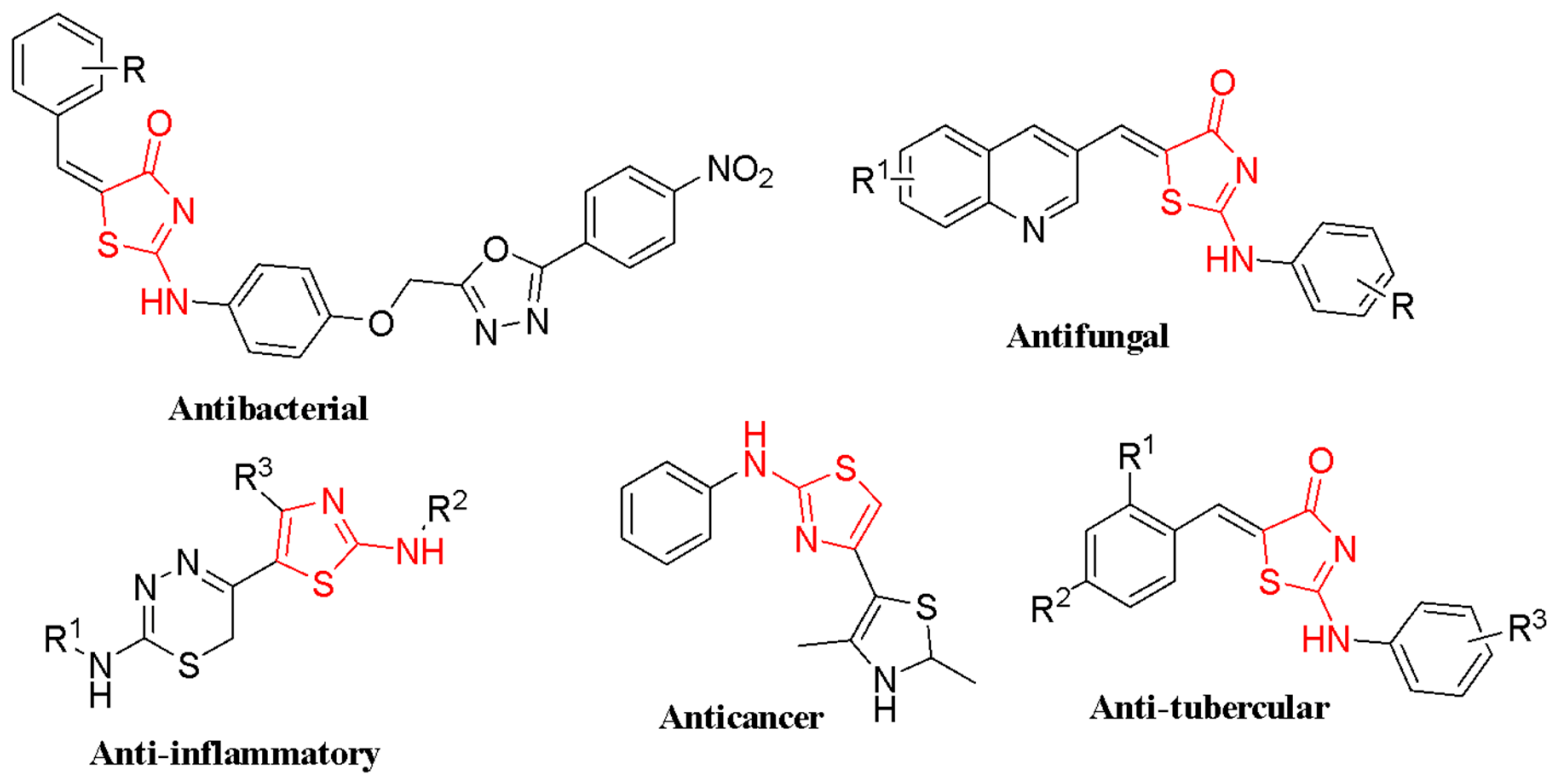
Representative biologically active molecules containing a thiazol moiety.

Within this context, thiazol-containing molecules have emerged as particularly attractive scaffolds in neurotherapeutic development. Recent medicinal chemistry studies show that thiazol-based scaffolds facilitate the development of multifunctional neuroprotective agents owing to their ability to simultaneously modulate oxidative stress, neuroinflammation, cholinergic dysfunction, mitochondrial impairment, and protein aggregation, all key contributors to AD pathogenesis [20,21]. The thiazol heterocycle, a five-membered ring containing sulfur and nitrogen, represents a privileged pharmacophore in medicinal chemistry, combining favorable physicochemical properties such as metabolic stability, hydrogen-bonding capacity, moderate lipophilicity, and substantial synthetic versatility, making it an attractive scaffold for designing drugs that target the central nervous system (CNS) (Figure 1) [20]. These features have enabled extensive structural diversification and have positioned thiazols as valuable scaffolds in numerous therapeutic areas, including neurodegenerative disease research.

Notably, thiazol derivatives exhibit a broad range of biological activities, including antioxidant, anti-inflammatory, antimicrobial, anticancer, antiviral, and neuroprotective effects, and the scaffold is represented in multiple clinically relevant drugs [22,23,24]. Recent studies further demonstrate that rationally designed thiazol derivatives can simultaneously target multiple pathological pathways implicated in AD, supporting their development as multifunctional neuroprotective agents [25,26]. Advances in synthetic methodologies, including multicomponent strategies, cyclization-based approaches, C–H functionalization methods, and environmentally sustainable catalytic systems have further expanded access to structurally diverse thiazol derivatives, facilitating rational structure optimization and accelerating the evaluation of their therapeutic potential [27,28,29,30,31,32,33].

Particularly relevant to neurodegeneration, thiazol-based compounds have emerged as promising multifunctional neuroprotective leads. Several thiazol and benzothiazol derivatives have demonstrated potent cholinesterase-related activity together with antioxidant, monoamine oxidase-modulating, and cytoprotective properties, supporting their development as multi-target-directed ligands [34,35]. Moreover, thiazol scaffolds have shown neuroprotective efficacy in diverse experimental models of neurodegeneration, including OS-driven cellular models and in vivo disease models, reinforcing their promise as pharmacophores capable of engaging both target-based and phenotypic neuroprotection mechanisms [23,36].

Among these, aminothiazol and thiazolone derivatives are particularly attractive because substitution around the thiazol core allows modulation of steric and electronic features relevant to target interaction and biological activity [37,38]. In particular, 2-(arylamino) thiazol-4(5H)-ones offer a synthetically accessible framework that can be structurally optimized to develop multifunctional agents targeting NDDs.

Given the multifactorial nature of AD, phenotypic screening approaches integrating cellular and in vivo models offer an effective strategy for identifying compounds with translational neuroprotective potential. Human SH-SY5Y cells treated with 6-Hydroxydopamine (6-OHDA) are widely used as a model of oxidative neuronal injury to evaluate cytoprotection, intracellular ROS accumulation, and mitochondrial dysfunction. Because OS and mitochondrial impairment are common pathological mechanisms shared among several NDDs, including AD, this model enables the assessment of the antioxidant and cytoprotective properties of candidate neuroprotective compounds [39,40]. Complementing this in vitro assay, transgenic *Drosophila melanogaster* expressing human Aβ42 exhibit key behavioral deficits and reduced lifespan associated with amyloid-mediated neurodegeneration, providing an AD-relevant in vivo platform for evaluating neuroprotective efficacy [41,42].

In the present study, we employed an improved microwave-assisted synthetic strategy to prepare a series of 2-(arylamino)thiazol-4(5H)-one derivatives and investigated their neuroprotective potential using integrated in silico, cellular, and *Drosophila* models. Molecular docking was employed to explore putative interactions with Acetylcholinesterase (AChE) as a target-guided design rationale, and selected compounds were further evaluated for their AChE inhibitory activity in vitro to experimentally assess docking predictions. Neuroprotective efficacy was assessed through cellular assays of viability, oxidative stress, and mitochondrial membrane integrity, together with behavioral and lifespan analyses in a *Drosophila* AD model. Our findings suggest that 2-(arylamino)thiazol-4(5H)-one derivatives exhibit AChE inhibitory activity and mitigate oxidative stress, conferring neuroprotection in complementary in vitro and in vivo models relevant to AD.

## 2. Results

### 2.1. Chemistry

Microwave-assisted organic synthesis (MAOS) has emerged as an efficient approach for heterocycle synthesis because of its reduced reaction times, improved yields, and compatibility with green chemistry principles. Building on our continued interest in heterocyclic compounds [43,44,45,46,47,48], we employed a microwave-assisted strategy for the synthesis of a series of 2-(arylamino)thiazol-4(5H)-one derivatives.

**Scheme 1:**
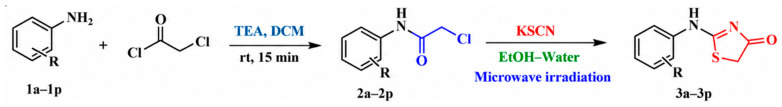
Synthetic route for 2-(arylamino)thiazol-4(5H)-ones.

Taking into account the importance of thiazolone derivatives in medicinal chemistry, we initiated the synthesis of 2-(arylamino)thiazol-4(5H)-ones (**3a**–**3p**) via an intramolecular cyclization reaction under microwave irradiation, employing an ethanol–water solvent system without the need for any acid, base, or catalyst. Compared to conventional thermal methods, this microwave-assisted protocol significantly reduced the reaction time while maintaining excellent yields. The synthetic route employed is illustrated in Scheme 1 [49,50,51,52]. Briefly, commercially available anilines were first treated with 2-chloroacetyl chloride to afford 2-chloro-*N*-aryl acetamides [44]. These intermediates underwent a subsequent reaction with KSCN in an ethanol–water medium under microwave irradiation (MWI) for 5 min, yielding the target compounds 2-(arylamino)thiazol-4(5H)-ones. This method is notable for being rapid, convenient, and environmentally friendly, providing an effective approach for the synthesis of target thiazol derivatives.

To synthesize the target compounds, we employed a stepwise optimization strategy. Initially, a model reaction was carried out using equimolar amounts of intermediate 2a and KSCN to identify suitable reaction conditions. The optimization was systematically performed by varying solvents, temperature, and reaction time, and the results are summarized in Table S1. The reaction performed in non-polar solvent toluene gave poor results, while dichloromethane improved the yield to 52% under identical conditions (Supplementary Table S1, Entries 1–2). Ether-based solvents such as 1,4-dioxane and THF provided moderate yields of 50% and 55%, respectively (Supplementary Table S1, Entries 3–4). The use of acetonitrile, a polar aprotic solvent, significantly enhanced the yield to 78%. High-boiling polar aprotic solvents such as DMF and DMSO further increased the yield to 82–84% (Supplementary Table S1, Entries 5–7). Interestingly, with the polar protic solvent, methanol yield of 85% was obtained, while ethanol afforded an even higher yield of 90% with a reduced reaction time of 5 min. Notably, the use of aqueous solvent water, a green and environmentally benign solvent, proved to be the most effective medium, providing the highest yield of 91%, but in 30 min (Supplementary Table S1, Entries 8–10). These findings indicated that polar protic solvents were most suitable for promoting the intramolecular cyclization reaction and guided the subsequent optimization of the ethanol–water solvent system under microwave irradiation.

To further refine the reaction conditions, the reaction was carried out under reflux in an ethanol–water (1:1) solvent mixture, which afforded an excellent yield of 93% within 25 min (Table 1, Entry 11). The conventional thermal method required prolonged heating, underscoring the advantages of microwave-assisted organic synthesis as a faster, more economical, efficient, and environmentally benign alternative. Therefore, we explored the reaction under microwave irradiation. Using ethanol as the solvent at 80 °C provided 90% yield, while replacing ethanol with water under identical conditions gave comparable results. Strikingly, when an ethanol–water (1:1) solvent system was employed under microwave irradiation at 80 °C, the yield further improved to 96% in 5 min (Table 1, Entries 12–14). This remarkable enhancement can be attributed to the superior absorption of microwave energy by polar solvents, which accelerates reaction rates and minimizes the formation of side products. The ethanol–water system serves as an effective polar protic organic co-solvent, combining the solubilizing power of ethanol with the green nature of water. Ethanol, in particular, is a non-toxic, environment friendly solvent with excellent microwave-absorbing capacity and the ability to dissolve a wide range of organic compounds. Together, this solvent combination offered the optimal balance of yield, reduced reaction time, and compliance with green chemistry principles for the intramolecular cyclization reaction.

To further optimize the reaction conditions under microwave irradiation, the model reaction was examined at room temperature, 50 °C, and 80 °C. The product yield increased progressively with temperature, with the highest yield obtained at 80 °C, indicating that elevated temperatures favor the intramolecular cyclization reaction. By optimizing both the solvent system and reaction temperature, the reaction time was reduced from 25 min under conventional reflux to only 5 min under microwave irradiation. The optimized conditions, comprising an ethanol–water (1:1) solvent system at 80 °C, were subsequently applied to the synthesis of a series of 2-(arylamino)thiazol-4(5H)-one derivatives (**3a**–**3p**). The reactions proceeded smoothly with 2-chloro-*N*-arylacetamides bearing both electron-donating and electron-withdrawing substituents, affording the desired products in consistently high yields (92–96%) within 4–5 min (Table 1). All synthesized compounds were characterized by ^1^H NMR, ^13^C NMR, and high-resolution mass spectrometry (HRMS). In addition, the lead compounds selected for biological evaluation (**3n**, **3i**, and **3b**) were analyzed by reversed-phase HPLC (Supplementary Figures S49–S52). Complete spectral and chromatographic data are provided in the Supplementary Materials (Sections S1 and S2).

### 2.2. Molecular Docking and Binding Interaction Analysis of 2-(Arylamino)thiazol-4(5H)-ones with AChE

Molecular docking was performed to predict binding modes of synthesized 2-(arylamino)thiazol-4(5H)-one derivatives within the active site of AChE (PDB ID: 4EY7). To validate the docking protocol, redocking of the co-crystallized donepezil was performed, yielding an RMSD of 0.432 Å. Molecular docking was subsequently simulated on the all the synthesized thiazol derivatives (**3a**–**3p**), and the binding conformations exhibiting the most favorable free energies were selected for interaction analysis. The results showed that all compounds were accommodated within the same active site occupied by the reference ligand, displaying favorable predicted binding free energies ranging from −7.153 to −8.638 kcal/mol (Supplementary Table S2, Supplementary Figure S53). Among the synthesized derivatives, **3n** exhibited the strongest predicted binding affinity (−8.638 kcal/mol), followed by 3i (−7.971 kcal/mol) and **3b** (−7.943 kcal/mol), whereas the unsubstituted derivative **3a** showed the lowest affinity (−7.153 kcal/mol).

Although all the synthesized compounds exhibited lower binding free energies than the co-crystallized reference ligand donepezil (−11.765 kcal/mol), several derivatives showed favorable interaction profiles within the AChE binding pocket. Compounds **3n**, **3i**, and **3b** were selected for further biological evaluation.

Detailed protein–ligand interaction analyses were performed for the three highest-ranked compounds to better understand the structural basis for their binding. The AChE binding pocket primarily comprises the mid-aromatic gorge, catalytic triad (Ser203, Glu334, His447), peripheral anionic site (Trp286, Tyr124, Asp74, Ser125, Phe295), omega loop, oxyanion hole, anionic sub-site (Gly448, Glu202, Ile451, Tyr133, Trp86), and acyl binding pocket (Supplementary Figure S54) [53]. Ligand binding within this gorge is predominantly driven by hydrophobic and aromatic interactions, with potent inhibitors typically interacting with multiple regions simultaneously to achieve high affinity.

**Figure 2:**
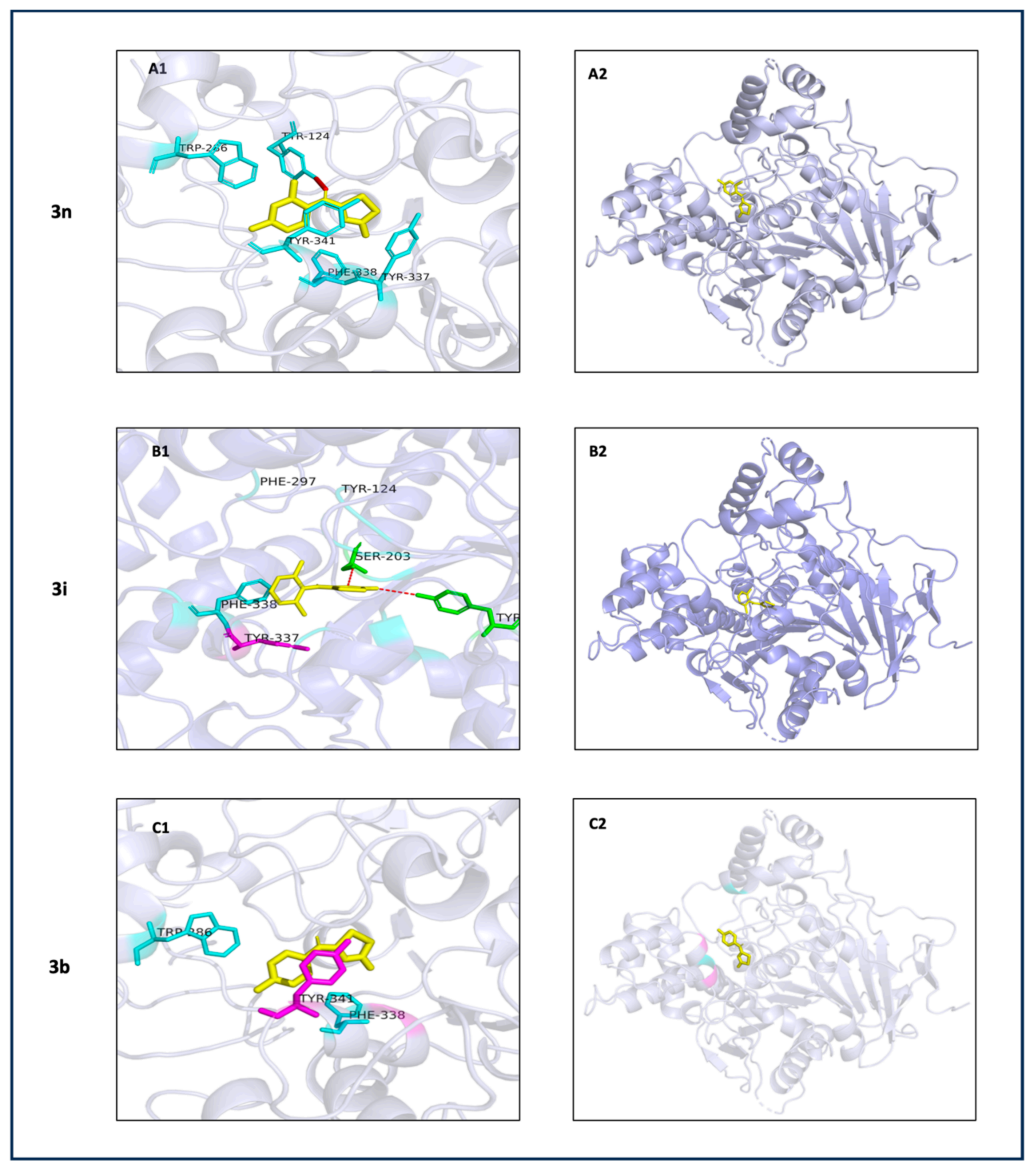
Protein–ligand interactions of compounds **3n**, **3i**, and **3b** with acetylcholinesterase (AChE; PDB ID: 4EY7) shown in 3D representations ((**A1**–**C1**) shows the zoomed-in and (**A2**–**C2**) shows the overall views).

Compound **3n** (binding energy: −8.638 kcal/mol) exhibited a well-defined binding orientation spanning both the peripheral anionic site and the aromatic gorge. The ligand formed π–π stacking interactions with key aromatic residues Tyr337, Phe338, and Tyr341, while a hydrogen bond interaction was observed with Tyr124. Additionally, hydrophobic contacts with Trp286 at the PAS region contributed to enhanced stabilization of the ligand within the binding pocket (Figure 2). This multi-site interaction profile suggests strong binding affinity and is consistent with the binding mode of known inhibitors such as donepezil and correlates with the superior docking score of **3n**.

Compound **3i** (binding energy: −7.971 kcal/mol) was primarily localized within the aromatic gorge and formed stabilizing interactions with residues Tyr337, Phe338, and Tyr341 through hydrophobic and π–π interactions (Figure 2). However, unlike compound **3n**, limited interaction with the PAS region was observed, which may account for its comparatively moderate binding affinity.

In contrast, compound **3b** (binding energy: −7.943 kcal/mol) was positioned closer to the catalytic active-site region and exhibited interactions with residues near the catalytic triad. Interaction analysis indicated hydrogen bonding with residues in the catalytic region, including Ser203 and His447. While this orientation suggests interaction near the catalytic machinery, the reduced involvement of aromatic gorge residues—particularly the absence of π–π stacking with Tyr341—likely results in comparatively lower binding stabilization (Figure 2).

Overall, compounds capable of simultaneously engaging both the PAS and aromatic gorge, particularly compound **3n**, exhibited the most favorable predicted binding profiles. These findings provided the rationale for selecting compounds **3n**, **3i**, and **3b** for subsequent in vitro evaluation of acetylcholinesterase inhibitory activity.

A detailed residue-level interaction analysis of the top compounds was performed using the Protein–Ligand Interaction Profiler (PLIP), and the results are summarized in Supplementary Table S3.

### 2.3. In Vitro AChE Inhibitory Activity

To evaluate the acetylcholinesterase (AChE) inhibitory activity of the selected compounds (**3n**, **3i**, and **3b**), their IC_50_ values were determined against acetylcholinesterase from *Electrophorus electricus* (EeAChE) using the well-established Ellman method [54,55,56,57]. Donepezil was included as the control. The inhibitory potency of each compound was expressed as the half-maximal inhibitory concentration (IC_50_) (Table 2).

The selected compounds inhibited AChE in a concentration-dependent manner, with compound 3n exhibiting the greatest inhibitory potency (IC_50_ = 12.03 ± 0.17 μM), followed by 3i (22.32 ± 0.26 μM) and 3b (29.51 ± 0.05 μM). As expected, the reference inhibitor donepezil displayed substantially greater potency, with an IC_50_ of 54.6± 1.5 nM.

### 2.4. In Silico Pharmacokinetics and Target Prediction of 2-(Arylamino)thiazol-4(5H)-one Derivatives

#### 2.4.1. In Silico Pharmacokinetics of 2-(Arylamino)thiazol-4(5H)-one Derivatives

Pharmacokinetic properties of the selected compounds (**3n**, **3i**, and **3b**) were predicted using the SwissADME web-based tool, with key physicochemical parameters summarized in Table 3. All investigated compounds were predicted to have ideal lipophilicity for oral and intestinal absorption (iLOGP value < 5) and exhibited water solubility. All selected compounds were predicted to have high human intestinal absorption percentages indicating their potentials to be used as oral drugs. All selected compounds were predicted to cross the blood–brain barrier (BBB) moderately, suggesting their potential to reach CNS targets.

Metabolic predictions focused on cytochrome P450 enzymes revealed that none of the compounds are likely to inhibit CYP2D6 or CYP3A4, two major isoforms involved in drug metabolism, suggesting a lower likelihood of related drug–drug interactions [58]. However, all compounds were predicted to inhibit CYP1A2, indicating possible interactions with metabolic pathways mediated by this enzyme. Drug-likeness assessments showed that all compounds complied with Lipinski’s and Veber’s rules, which is comparable to the reference drug donepezil.

**Figure 3:**
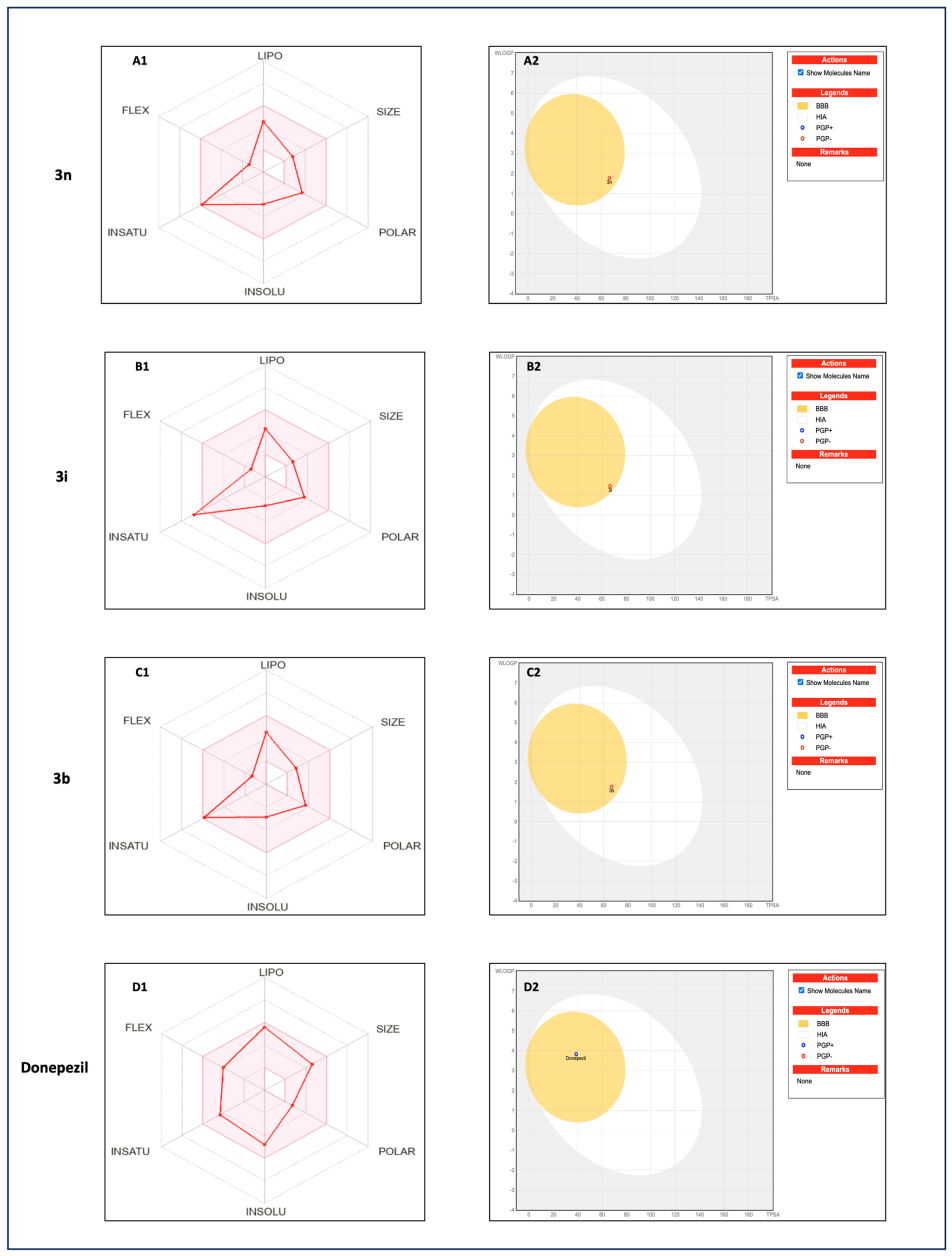
BOILED Egg plot radar graph showing the chemical structure and physicochemical properties of compounds (**3n**, **3i**, and **3b**) and reference compound donepezil. Bioavailability radar plots of (**A1**) compound **3n**, (**B1**) compound **3i**, (**C1**) compound **3b**, and (**D1**) donepezil and corresponding BOILED-Egg plots (**A2**–**D2**).

The physicochemical properties of compounds **3n**, **3i**, and **3b** were analyzed using a radar (spider) plot, illustrating key drug-likeness parameters: lipophilicity (LIPO), molecular size (SIZE), polarity (POLAR), solubility (INSOLU), flexibility (FLEX), and saturation (INSATU). The red lines depict the profiles of these compounds, while the shaded area represents the optimal range for drug-like characteristics. All three compounds largely fell within the acceptable limits, suggesting favorable physicochemical characteristics comparable to the reference drug donepezil (Figure 3).

Pharmacokinetic evaluation using the BOILED-Egg model placed compounds **3n**, **3i**, and **3b** within the yellow region, signifying a high likelihood of BBB penetration, a critical attribute for CNS therapeutics (Figure 3). The white region, indicative of high gastrointestinal (GI) absorption, was also considered. Additionally, these compounds are predicted to be P-glycoprotein negative (P-gp), suggesting a lower risk of efflux from the brain. Collectively, these findings support the favorable physicochemical and pharmacokinetic properties of compounds **3n**, **3i**, and **3b**, reinforcing their potential for further evaluation as neuroactive drug candidates.

#### 2.4.2. Target Prediction of 2-(Arylamino)thiazol-4(5H)-one Derivatives

Web-based target prediction tools were employed to identify potential targets of the selected thiazol derivatives (**3n**, **3i**, and **3b**) and AD-related targets. Results obtained from two databases, namely SwissTargetPrediction and SEA, yielded a total of 128 unique potential targets associated with the compounds. Additionally, a dataset of 275 AD-related genes was retrieved from GeneCards and DisGeNET databases.

**Figure 4:**
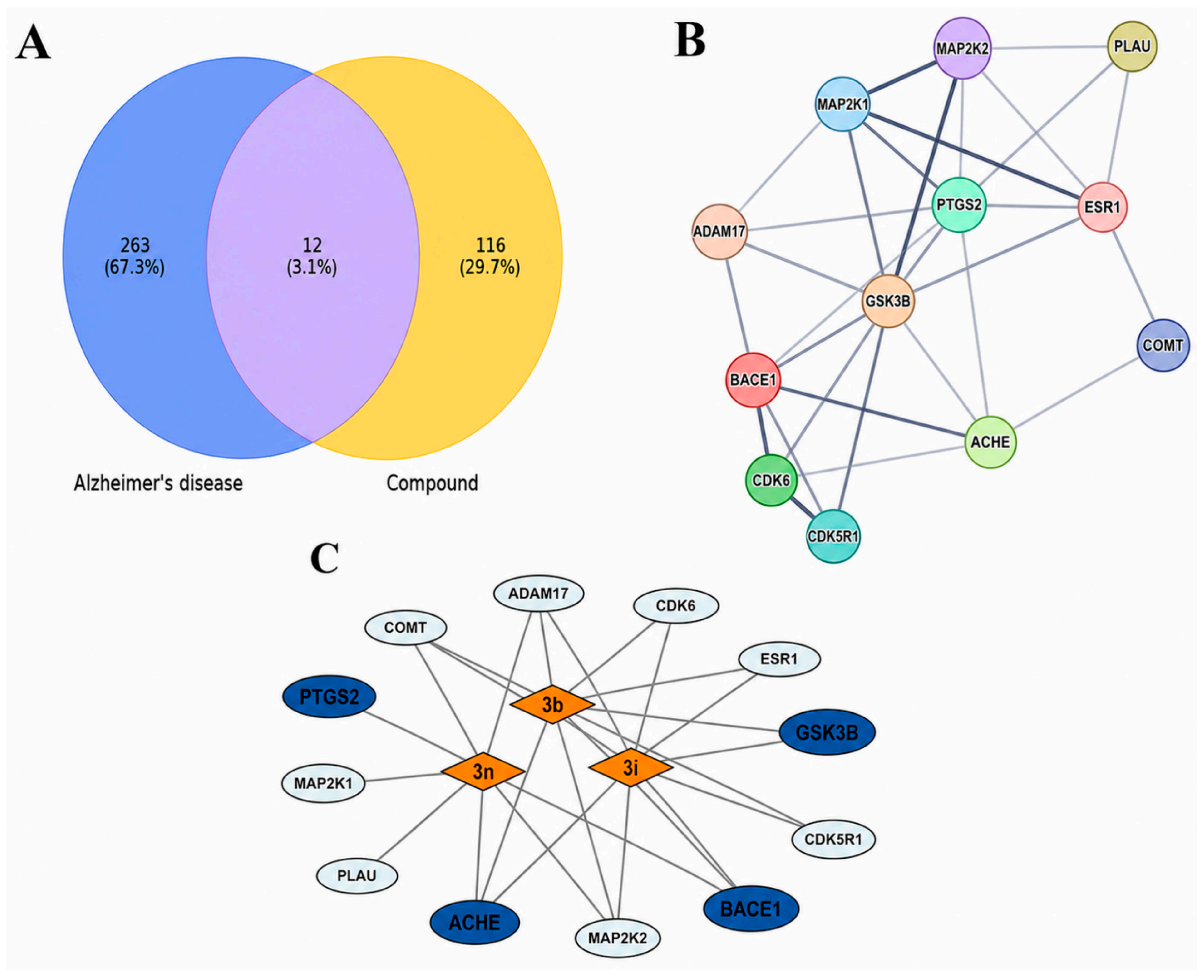
Illustration of the PPI network mapping. Common targets between the AD-related proteins and predicted protein targets of 2-(arylamino)thiazol-4(5H)-ones are represented in the (**A**) Venn diagram and (**B**) PPI network diagram of the AD-related proteins (confidence level > 0.4) in the STRING database. Nodes represent proteins, and edges represent functional associations. Edge thickness indicates the strength of interaction. (**C**) Compound-target network. 2-(Arylamino)thiazol-4(5H)-ones are represented as orange diamonds, while AD-related proteins are represented as blue circles, where the major targets are highlighted with dark blue color and larger size.

The overlapping targets between the compound-associated targets and AD-related genes were identified using a Venn diagram, which revealed 12 common targets, suggesting their potential relevance to AD pathology (Figure 4A).

Subsequently, these 12 common targets were imported into the STRING database to construct a protein–protein interaction (PPI) network, which was further visualized using Cytoscape 3.10.1 software. The resulting network revealed significant interactions among the targets, indicating their functional interconnectivity in neurodegenerative processes (Figure 4B).

Topological analysis of the PPI network identified several hub targets with higher degree centrality (DC), including glycogen synthase kinase 3 beta (GSK3B), prostaglandin-endoperoxide synthase 2 (PTGS2), beta-secretase 1 (BACE1), estrogen receptor 1 (ESR1), mitogen-activated protein kinase 1 (MAPK1), acetylcholinesterase (ACHE), and mitogen-activated protein kinase kinase 1 (MAP2K1). These targets were identified as core targets and were further analyzed based on their betweenness centrality (BC) and closeness centrality (CC) values (Table 4).

To further elucidate the interaction between the selected compounds and their predicted targets, a compound–target interaction network was constructed. The network revealed that multiple targets are shared among the compounds, indicating a multi-target therapeutic potential, which may be particularly relevant for complex diseases such as AD (Figure 4C).

#### 2.4.3. KEGG Pathway Enrichment Analysis

**Figure 5:**
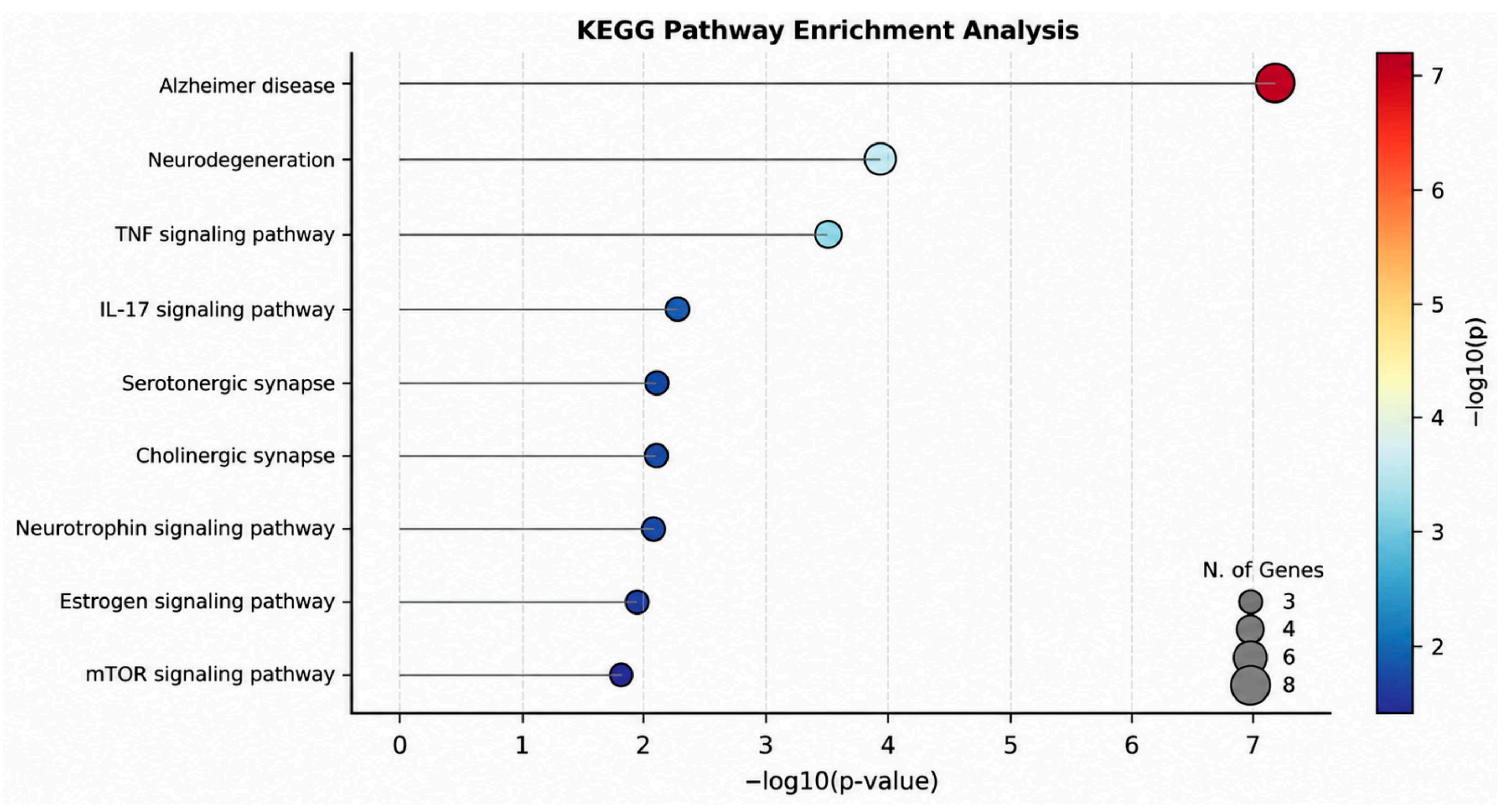
KEGG pathway enrichment analysis of overlapping targets. The top significantly enriched pathways are ranked by −log_10_(*p*-value). The color intensity reflects the enrichment significance, and the bubble size indicates the gene ratio. Pathways related to neurodegeneration (Alzheimer’s disease, pathways of neurodegeneration), neuroinflammation (TNF signaling, IL-17 signaling), synaptic transmission (cholinergic synapse, serotonergic synapse), and neuronal survival (neurotrophin signaling, mTOR signaling, estrogen signaling) were among the most significantly enriched. The horizontal dashed line denotes the significance threshold (*p* = 0.05, −log_10_(*p*) = 1.3).

To explore the potential molecular mechanisms of the identified targets, KEGG pathway enrichment analysis was performed (Figure 5). The overlapping targets were significantly enriched in pathways associated with neurodegeneration and inflammation, including AD, pathways of neurodegeneration, TNF signaling pathway, IL-17 signaling pathway, serotonergic synapse, cholinergic synapse, neurotrophin signaling pathway, estrogen signaling pathway, and mTOR signaling pathway (Figure 5).

Notably, several of these pathways are closely linked to the key pathological features of AD, including amyloid-beta accumulation, tau hyperphosphorylation, neuroinflammation, and synaptic dysfunction. For instance, BACE1 is directly involved in amyloid-beta production via the amyloidogenic pathway, whereas GSK3B plays a critical role in tau phosphorylation and neurofibrillary tangle formation [59,60]. Additionally, inflammatory mediators such as PTGS2 (COX-2) and signaling molecules including MAPK1 and MAP2K1 are implicated in neuro-inflammatory and stress-response cascades [61,62,63]. These findings suggest that the predicted targets of the selected compounds are associated with multiple AD-related signaling pathways involved in amyloid processing, neuroinflammation, synaptic function, and neuronal survival, supporting their potential relevance to AD.

Gene Ontology enrichment analysis further indicated the involvement of the identified targets in biological processes such as amyloid-beta response, neuronal apoptosis, and synaptic function (Supplementary Figure S55).

### 2.5. In Vitro Studies

#### 2.5.1. Cytotoxicity of 2-(Arylamino)thiazol-4(5H)-one Derivatives Against Neuroblastoma SH-SY5Y Cell Line

The cytotoxicity of the thiazol derivatives on the SH-SY5Y cell line was assessed using the MTT assay (Table 5). Dose–response curves for all tested compounds were fitted using a four-parameter variable-slope model (R^2^ > 0.98) and IC_50_ values were determined with corresponding 95% confidence intervals, indicating concentration-dependent cytotoxic effects in the neuroblastoma cell line (Supplementary Figure S56).

Based on these findings, subsequent neuroprotection experiments were performed using concentrations well below the respective IC_50_ values to minimize compound-induced cytotoxicity.

#### 2.5.2. Effect of 2-(Arylamino)thiazol-4(5H)-one Derivatives on Cell Viability of 6-OHDA-Induced SH-SY5Y Cells

The impact of 2-(arylamino)thiazol-4(5H)-one derivatives (**3a**–**3p**) on the viability of neuroblastoma SH-SY5Y cells was evaluated using the MTT assay. 6-Hydroxydopamine (6-OHDA), a neuro-toxic agent commonly employed to induce OS, was used to model mitochondrial dysfunction relevant to AD [64,65]. Although traditionally applied in Parkinson’s disease research, 6-OHDA effectively generates mitochondrial OS and ROS, making it suitable for investigating neuroprotective mechanisms associated with early AD pathology [66,67].

Exposure of SH-SY5Y cells to 100 μM 6-OHDA for 24 h resulted in a significant decrease in cell viability to approximately 58% relative to untreated controls (100%), confirming the induction of oxidative stress-induced neuro-toxicity. This outcome is consistent with the existing literature that attributes 6-OHDA’s neuro-toxicity to mitochondrial impairment and ROS accumulation, which are key factors implicated in the early stages of AD [68].

**Figure 6:**
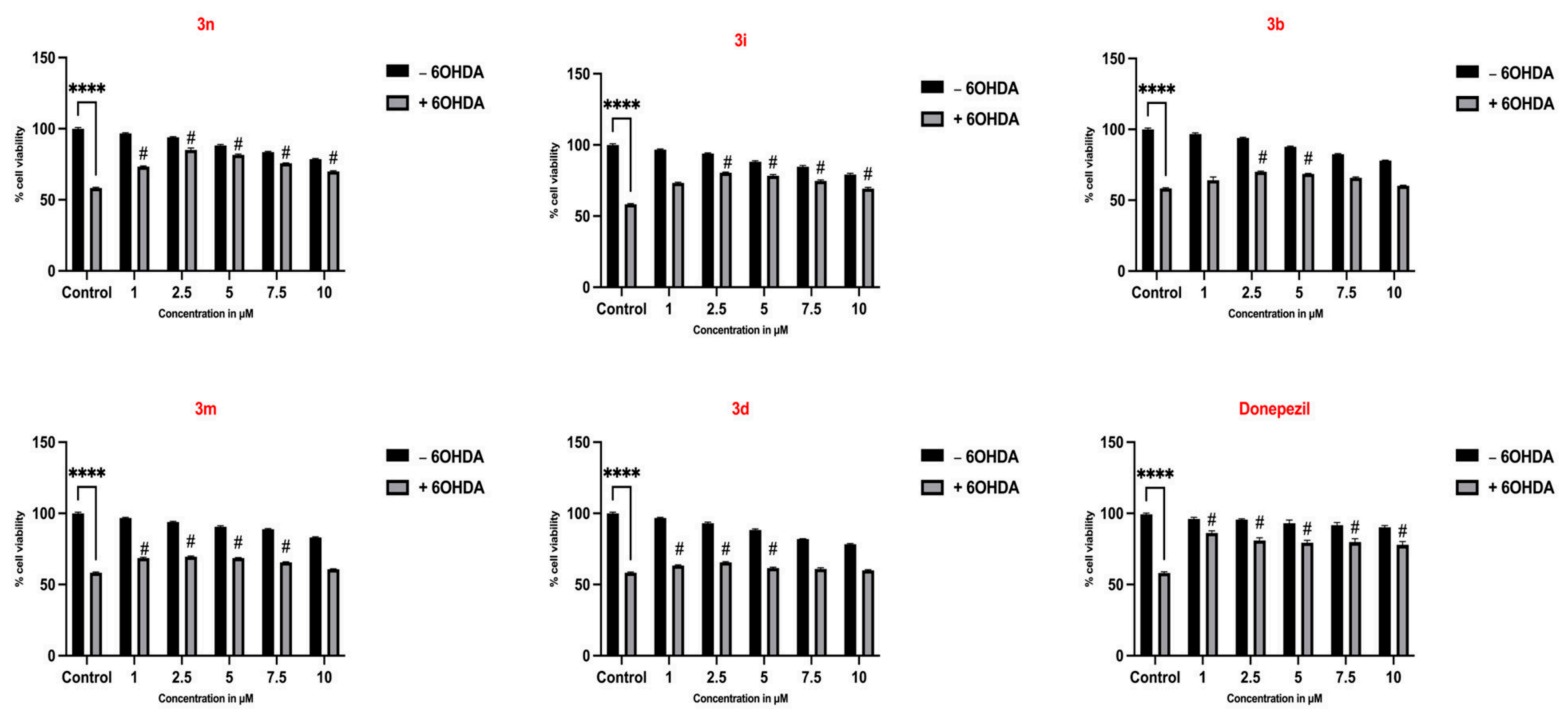
Neuroprotective activity of selected 2-(arylamino)thiazol-4(5H)-one derivatives and donepezil on 6-OHDA-induced SH-SY5Y neuronal cells. Cell viability was assessed using the MTT assay (*N* = 5). Data are reported as the mean ± SEM and were statistically analyzed with one-way ANOVA. **** *p* ≤ 0.0001 compared to the control group. # *p* ≤ 0.05 compared to the 6-OHDA-induced group. Dose-dependent responses for other synthesized derivatives are shown in Supplementary Figure S57.

To determine the intrinsic cytotoxicity of the synthesized derivatives, cells were treated with concentrations ranging from 1 to 10 μM, selected based on prior IC_50_ data to ensure non-toxic levels. No significant reduction in cell viability was observed at these concentrations, indicating that these concentrations did not produce significant cytotoxicity under the experimental conditions. Consistent with previous findings, treatment with 100 μM 6-OHDA alone for 24 h caused approximately a 40% decrease in viability, as illustrated in Figure 6.

Neuroprotective potential was assessed by pre-treating SH-SY5Y cells with each derivative for 4 h prior to 24 h exposure to 100 μM 6-OHDA (Figure 6, Supplementary Figure S57). Donepezil, a well-known AChE inhibitor, was used as a reference compound. The results indicated that pretreatment with donepezil at 1–10 μM increased the cell viability of 6-OHDA-induced cells, restoring cell viability to approximately 86% compared with the 6-OHDA group (*p* ≤ 0.05). Among the synthesized derivatives, compounds **3d**, **3m**, **3b**, **3i**, and **3n** at 2.5 μM significantly improved cell viability, restoring it to 65–85% relative to 6-OHDA-treated cells (Figure 6, Supplementary Figure S57). Notably, compound **3n** showed the strongest effect, restoring viability to approximately 85%. Based on its superior neuroprotective activity, together with its favorable molecular docking profile and AChE inhibitory activity, compound **3n** emerged as the most promising derivative in this series. Therefore, **3n**, **3i**, **3b**, **3m**, and **3d** were selected for subsequent mechanistic studies at a concentration of 2.5 μM.

#### 2.5.3. Effect of the Selected Compounds on Morphological Changes in the Cells

**Figure 7:**
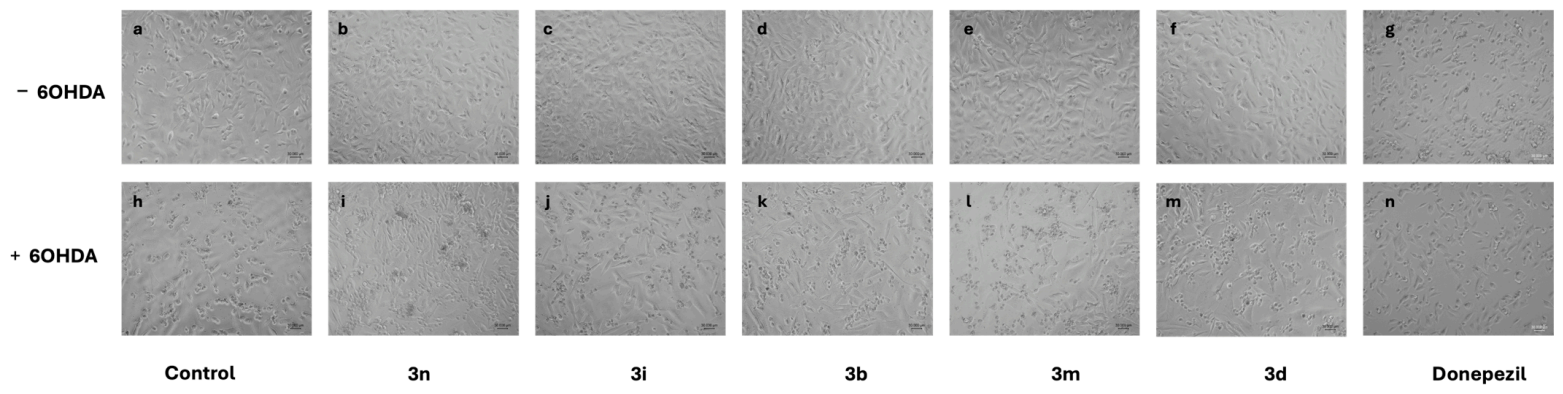
Representative bright-field microscopy images showing the morphology of SH-SY5Y cells pretreated with 2.5 μM of the selected derivatives (**3n**, **3i**, **3b**, **3m**, and **3d**) or donepezil, in the presence or absence of 100 μM 6-OHDA. Control cells after seeding for 24 h (**a**); Cells treated with 6-ohda only (**h**); Cells treated with **3n** alone for 24 h and co-treated with **3n** and 6-ohda ((**b**) and (**i**), respectively); Cells treated with **3i** alone for 24 h and co-treated with **3i** and 6-ohda ((**c**) and (**j**), respectively); Cells treated with **3b** alone for 24 h and co-treated with **3b** and 6-ohda ((**d**) and (**k**), respectively); Cells treated with **3m** alone for 24 h and co-treated with **3m** and 6-ohda ((**e**) and (**l**), respectively); Cells treated with **3d** alone for 24 h and co-treated with **3d** and 6-ohda ((**f**) and (**m**), respectively); Cells treated with donepezil alone for 24 h and co-treated with donepezil and 6-ohda ((**g**) and (**n**), respectively). Images were acquired at 20× magnification. Scale bar = 30 μm.

Morphological changes in SH-SY5Y cells were examined using bright-field microscopy to evaluate the protective effects of the selected compounds (**3n**, **3i**, **3b**, **3m**, and **3d**). Cells exposed to 100 μM 6-OHDA exhibited clear morphological alterations, including shrinkage, detachment, and a transition to a small, rounded shape, compared to untreated controls (Figure 7). In contrast, cells pretreated with donepezil or thiazol derivatives at 2.5 μM for 4 h prior to 6-OHDA exposure showed markedly reduced morphological damage. These cells maintained a more intact structure, exhibited less cellular damage, and demonstrated better growth and confluence relative to cells exposed to 6-OHDA without pretreatment (Figure 7). These observations indicate that pretreatment with the selected compounds effectively mitigated the aberrant morphological changes induced by OS.

Among the tested derivatives, compound **3n** exhibited the greatest preservation of normal cellular morphology, comparable to that observed with donepezil.

#### 2.5.4. Effect of Selected Compounds on Intracellular ROS Production

**Figure 8:**
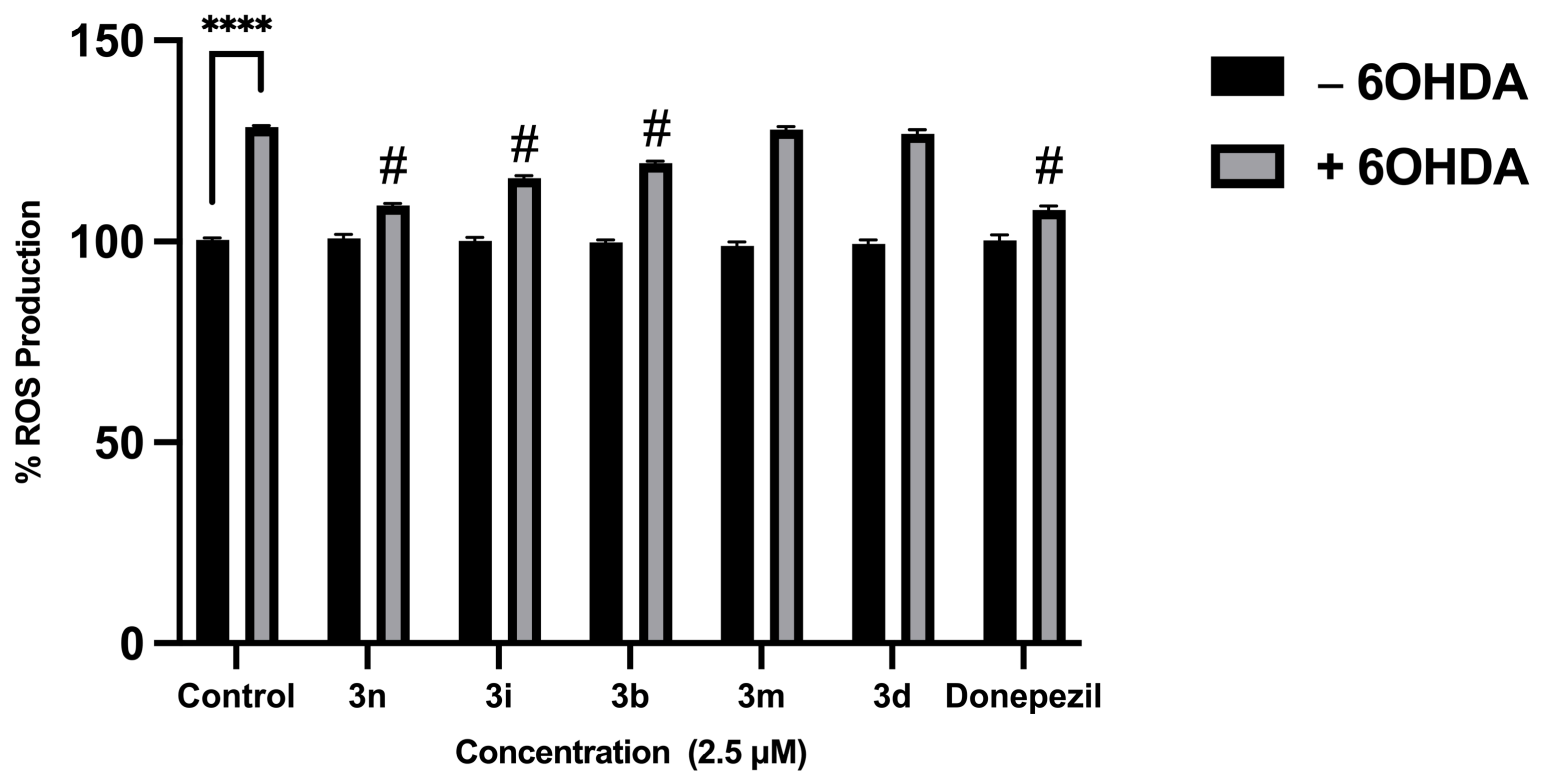
Quantification of intracellular ROS levels in SH-SY5Y cells following 6-OHDA exposure (*N* = 5). The SH-SY5Y cells were pretreated with 2.5 μM of thiazol derivatives or donepezil for 4 h followed by 100 μM of 6-OHDA for 24 h. Intracellular ROS levels were measured using the carboxy-DCFDA assay. The data are expressed as mean ± SEM, and statistical analysis was performed using one-way ANOVA. **** *p* < 0.0001 compared with the control cells; # *p* < 0.05 compared with the 6-OHDA.

**Figure 9:**
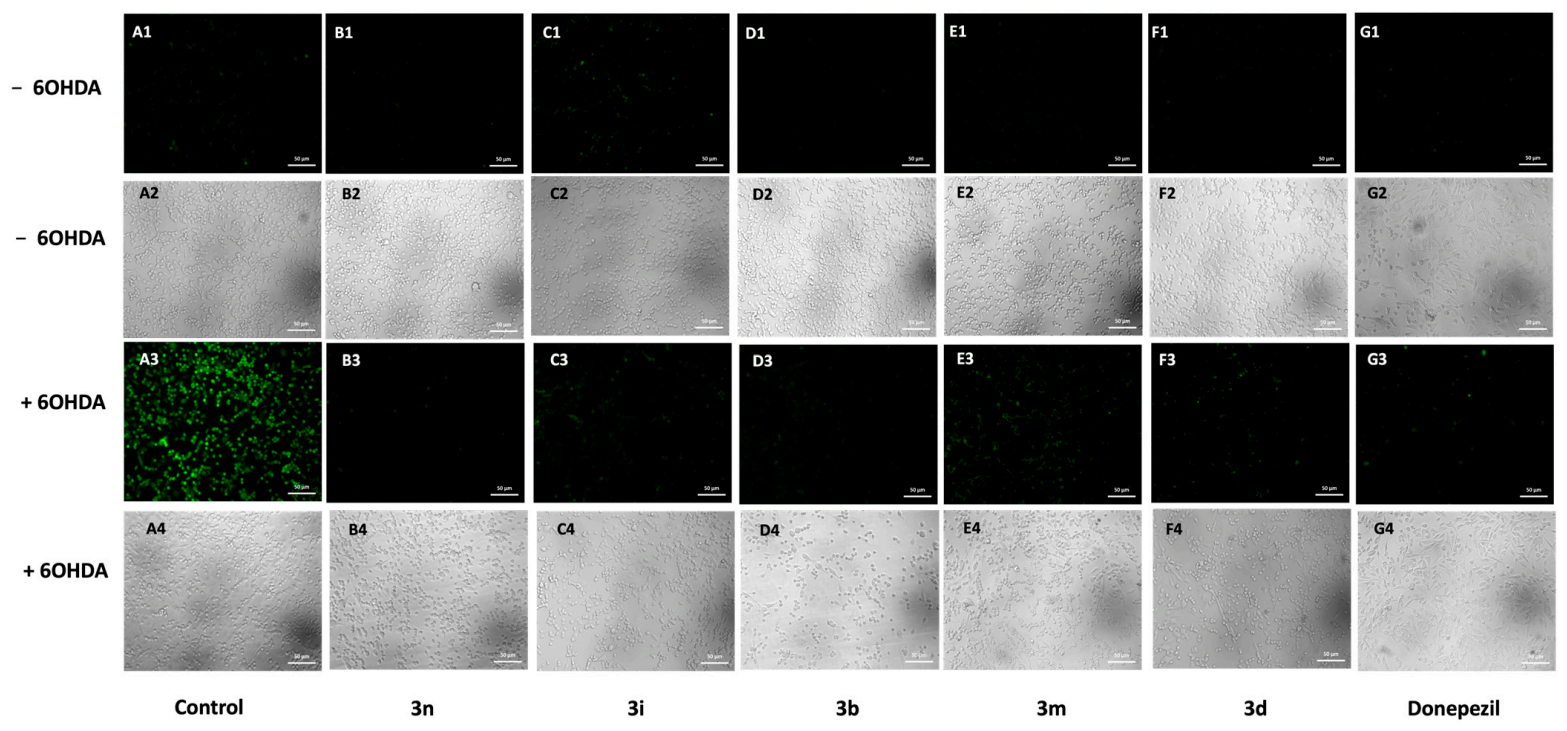
Representative fluorescence images of intracellular ROS detected using the carboxy-DCFDA assay in SH-SY5Y cells pretreated with compounds-**3n**, **3i**, **3b**, **3m**, and **3d** or donepezil for 4 h prior to exposure to 6-OHDA for 24 h. Control cells after seeding for 24 h (**A1**,**A2**); Cells treated with **3n** alone for 24 h (**B1**,**B2**); Cells treated with **3i** alone for 24 h (**C1**,**C2**); Cells treated with **3b** alone for 24 h (**D1**,**D2**); Cells treated with **3m** alone for 24 h (**E1**,**E2**); Cells treated with **3d** alone for 24 h (**F1**,**F2**); Cells treated with donepezil alone for 24 h (**G1**,**G2**); Cells treated with 6-OHDA alone for 24 h (**A3**;**A4**); Cells treated with **3n** for 4 h and then treated with 6-OHDA for 24 h (**B3**,**B4**); Cells treated with **3i** for 4 h and then treated with 6-OHDA for 24 h (**C3**,**C4**); Cells treated with **3b** for 4 h and then treated with 6-OHDA for 24 h (**D3**,**D4**); Cells treated with **3m** for 4 h and then treated with 6-OHDA for 24 h (**E3**,**E4**); Cells treated with **3d** for 4 h and then treated with 6-OHDA for 24 h (**F3**,**F4**); Cells treated with donepezil for 4 h and then treated with 6-OHDA for 24 h (**G3**,**G4**). Scale bar = 50 μm.

The effect of the selected compounds, i.e., **3n**, **3i**, **3b**, **3m**, and **3d,** on intracellular ROS levels were investigated using a fluorescence probe, carboxy-DCFDA, to reveal their antioxidant potential. Exposure of SH-SY5Y cells to 100 μM 6-OHDA for 24 h significantly increased intracellular ROS levels to 128.5 ± 0.5% relative to the untreated control group (Figure 8 and Figure 9). Treatment with the selected compounds or donepezil alone did not significantly alter basal ROS levels. However, pretreatment with 2.5 μM of each compound or donepezil for 4 h prior to 6-OHDA exposure significantly attenuated ROS accumulation. Among the tested derivatives, compound **3n** exhibited the greatest ROS suppressing effect, reducing ROS levels to 108.98 ± 0.7%, which was comparable to that of the reference compound donepezil (107.82 ± 0.85%) (*p* < 0.05). These findings indicate that the selected thiazol derivatives effectively reduce 6-OHDA-induced OS by suppressing intracellular ROS accumulation. These results are consistent with the enhanced neuroprotective effects of compound **3n** observed in cell viability and morphological analyses.

#### 2.5.5. Effect of the Selected Compounds on Mitochondrial Membrane Potential

**Figure 10:**
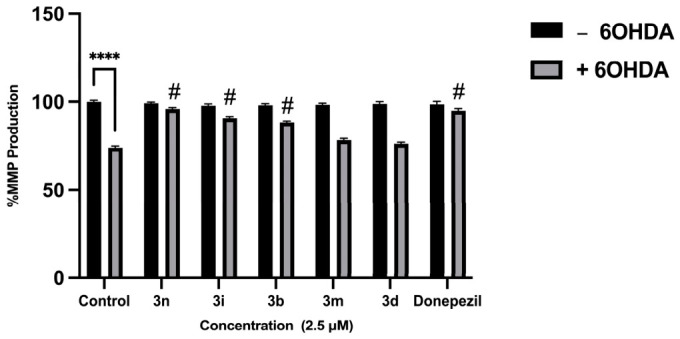
Effects of pretreatment with 2.5 μM of the selected thiazol derivatives or donepezil for 4 h on MMP in SH-SY5Y cells subsequently exposed to 100 μM 6-OHDA for 24 h. MMP was assessed by measuring rhodamine-123 fluorescence intensity (*N* = 5). Data are presented as the mean ± SEM and were analyzed using one-way ANOVA. **** *p* < 0.0001 compared to the control cells; ^#^ *p* < 0.05 compared with the 6-OHDA.

Mitochondrial membrane potential (MMP), a key indicator of mitochondrial function and early apoptosis, was assessed using the mitochondrial-specific fluorescent probe rhodamine-123 to evaluate the neuroprotective effects of compounds **3n**, **3i**, **3b**, **3m**, and **3d**. The assay measured fluorescence intensity corresponding to dye accumulation driven by the mitochondrial electrochemical gradient, where decreased fluorescence indicates reduced MMP and mitochondrial dysfunction (Figure 10). Exposure of SH-SY5Y cells to 100 μM 6-OHDA for 24 h significantly reduced MMP to 74.08 ± 1.04% compared to the untreated control group. Treatment with the selected compounds or donepezil alone did not significantly affect basal MMP levels. However, pretreatment with 2.5 μM of each compound or donepezil for 4 h prior to 6-OHDA exposure significantly attenuated the loss of MMP. Among the tested derivatives, compounds **3n** and **3i** exhibited the greatest protective effects, restoring MMP to 96.10 ± 1.07% and 90.72 ± 0.71%, respectively, comparable to the reference compound donepezil (95.08 ± 0.60%). Compounds **3b**, **3m**, and **3d** also significantly improved MMP relative to the 6-OHDA-treated group, although to a lesser extent. These findings indicate that pretreatment with the selected thiazol derivatives effectively preserves mitochondrial membrane potential and protects against 6-OHDA-induced mitochondrial dysfunction.

### 2.6. In Vivo Studies

#### 2.6.1. Effect of Compound **3n** on Motor Performance in the Drosophila AD Model

**Figure 11:**
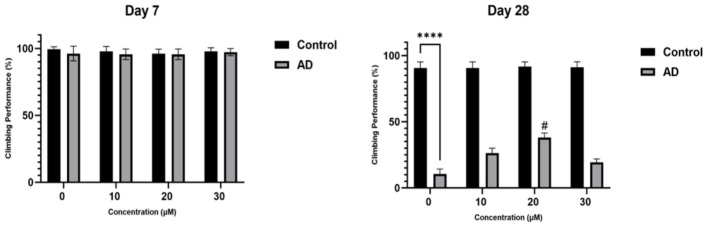
Climbing performance (%) of control and Aβ42 flies under untreated conditions and following treatment with different concentrations of compound **3n** (10 µM, 20 µM and 30 µM) at days 7 and 28 (*N* = 100). **** *p* ≤ 0.0001 compared to the control group. # *p* ≤ 0.05 compared to the Aβ42 group.

To evaluate whether compounds **3n**, **3i**, and **3b** could ameliorate behavioral deficits associated with AD, locomotor function was assessed using a negative geotaxis assay in Aβ42-expressing *Drosophila*. Aβ42 flies exhibit age-dependent neurodegeneration, with pronounced deficits emerging by approximately 4 weeks of age, and climbing performance was specifically analyzed at days 7 and 28. At day 7, no significant impairment in locomotor performance was observed. However, by day 28, untreated Aβ42 flies showed a marked decline in climbing performance, with climbing ability reduced to approximately 10%, indicating progressive neurodegeneration (*p* < 0.001, 2-way ANOVA with Dunnett’s post hoc multiple comparisons test). Treatment with compound **3n** significantly improved locomotor performance, with ~20% recovery observed at 20 μM (Figure 11). These findings demonstrate that compound **3n** reduces Aβ42-induced behavioral deficits in *Drosophila* in a time- and dose-dependent manner.

#### 2.6.2. Effect of Compound **3n** on Lifespan in the Drosophila AD Model

**Figure 12:**
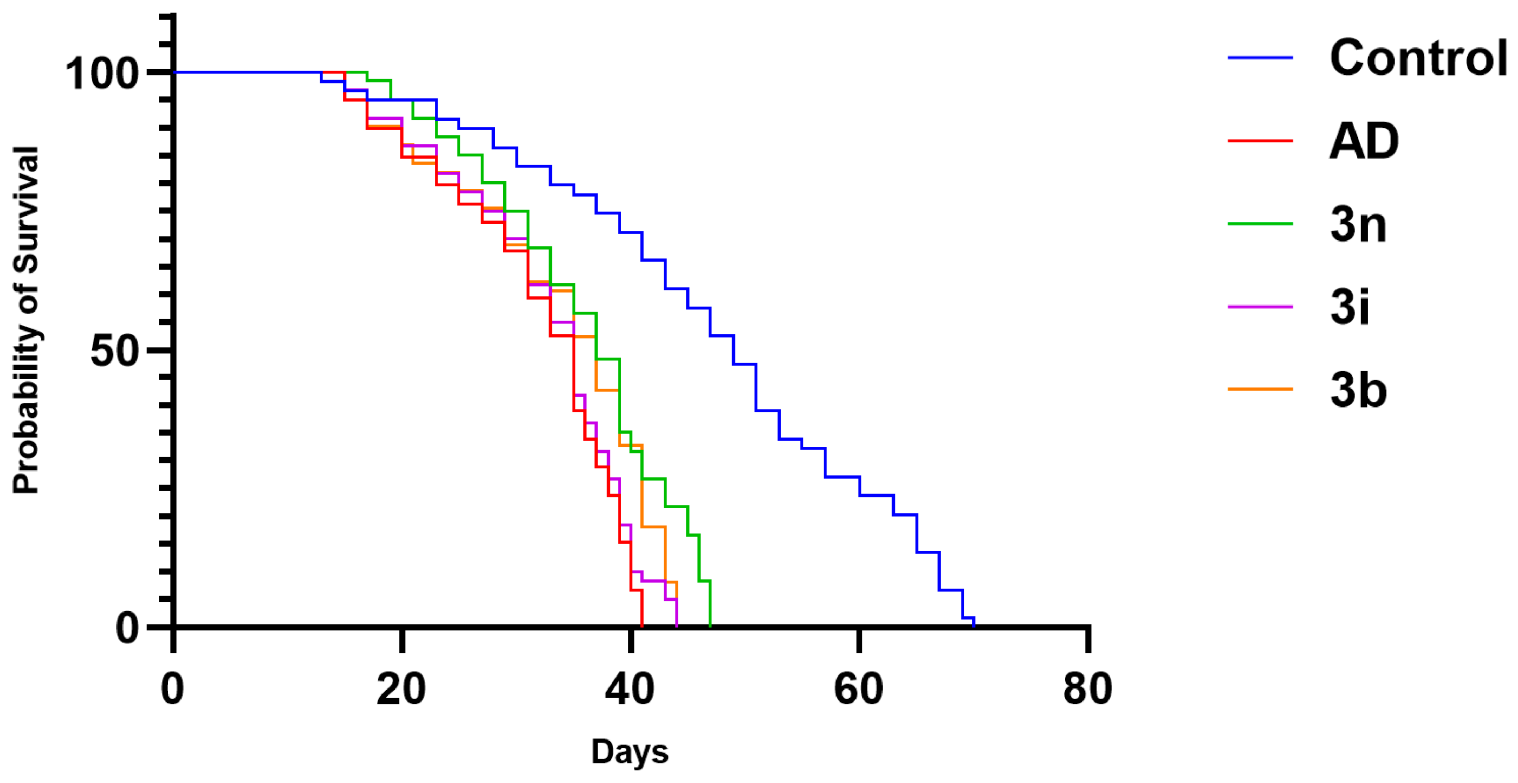
Kaplan–Meier survival curves of control and Aβ42-expressing *Drosophila* under normal conditions and upon treatment with compounds **3n**, **3i**, and **3b** (*N* = 100) at a concentration of 20 µM. Kaplan–Meier survival curves show a moderate reduction in lifespan in Aβ42 flies compared to controls.

To further evaluate the in vivo neuroprotective potential of compounds **3n**, **3i** and **3b**, their effect on lifespan was assessed in a *Drosophila* model of AD. Pan-neuronal overexpression of human Aβ42 using the elav-GAL4 driver resulted in a marked reduction in lifespan compared to control flies, which survived up to approximately 60 days. In contrast, Aβ42-expressing flies exhibited a shortened lifespan of approximately 40 days. Treatment with compound **3n** (20 µM) did not significantly alter the lifespan of control flies. However, in Aβ42-expressing flies, treatment with **3n** resulted in a moderate extension of lifespan, with treated flies surviving approximately 7 days longer than untreated AD flies (Figure 12). However, compounds **3i** and **3b** did not produce any improvements in lifespan compared to untreated Aβ42 flies. These findings indicate that compound **3n** partially alleviates Aβ42-induced phenotypes in vivo and further supports its neuroprotective potential in the *Drosophila* AD model.

## 3. Discussion

AD is the most prevalent neurodegenerative disorder and a leading cause of death among the elderly, characterized by progressive neuronal loss that results in cognitive decline and memory impairment [69]. Epidemiological data show that AD prevalence rises sharply with age, with a lifetime risk of about 15% in individuals over 65 and up to 40% in those older than 80 [70].

AD pathogenesis is complex and multifactorial, involving Aβ aggregation, tau hyperphosphorylation, OS, neuroinflammation, and cholinergic dysfunction [71]. Current therapeutic strategies are primarily symptomatic, focusing on the modulation of neurotransmitters such as acetylcholine and glutamate [72]. Clinically approved drugs, including AChE inhibitors (donepezil, rivastigmine, and galantamine) and N-methyl-D-aspartate (NMDA) receptor antagonists, provide only temporary symptomatic relief and are often accompanied with adverse effects such as gastrointestinal disturbances and hepatotoxicity [73,74]. Thus, there is an increasing interest in developing multifunctional therapeutic agents capable of simultaneously targeting several pathological mechanisms involved in AD.

Among the proposed therapeutic strategies, the cholinergic hypothesis remains a central approach in AD drug development. The inhibition of AChE prevents the hydrolysis of acetylcholine, thereby enhancing cholinergic neurotransmission [75,76,77,78]. Moreover, compounds capable of interacting with both the catalytic anionic site (CAS) and the peripheral anionic site (PAS) may additionally interfere with Aβ aggregation. Consequently, dual-site AChE inhibitors have attracted considerable attention as promising multifunctional anti-Alzheimer’s agents [53]. Notably, compounds such as donepezil, which interact with both CAS and PAS, often exhibit enhanced inhibitory activity and may also modulate Aβ aggregation, making dual-site binding a desirable feature in rational drug design [79,80,81,82].

Beyond cholinesterase inhibition, emerging strategies emphasize targeting multiple pathological pathways simultaneously. Key enzymes, such as β-secretase (BACE1), γ-secretase, and carbonic anhydrase, along with proteins involved in inflammation and OS, represent promising therapeutic targets [83,84]. Hence, the design of multi-target-directed ligands (MTDLs) has gained considerable attention as a promising strategy for managing complex NDDs such as AD [85].

In this context, heterocyclic scaffolds, particularly thiazol and thiazolidine derivatives, have demonstrated diverse pharmacological activities, including neuroprotective, anti-inflammatory, antioxidant, and enzyme inhibitory effects [86,87]. Several studies have reported that thiazol-based compounds exhibit significant inhibitory activity against AChE and other AD-related targets, as well as the ability to interfere with Aβ and tau aggregation pathways [88,89,90]. Collectively, these properties make thiazol derivatives attractive candidates for the development of novel anti-Alzheimer agents.

Accordingly, in the present study, we synthesized a series of 2-(arylamino)thiazol-4(5H)-one derivatives using a microwave-assisted one-pot approach and evaluated their neuroprotective potential through molecular docking, in vitro AChE inhibition, SH-SY5Y cell-based assays, and an Aβ42-expressing *Drosophila melanogaster* model. Among the synthesized derivatives, compound **3n** consistently exhibited strong biological profile, demonstrating the favorable predicted AChE binding, the lowest AChE IC_50_, enhanced cytoprotection, the attenuation of intracellular ROS generation, the preservation of mitochondrial membrane potential, and improved locomotor performance in vivo.

From a synthetic perspective, the microwave-assisted one-pot protocol provided an efficient approach for the preparation of the target 2-(arylamino)thiazol-4(5H)-one derivatives. Compared to conventional synthetic methods reported for related thiazol scaffolds, microwave-assisted synthesis offers advantages including shorter reaction times, operational simplicity, and improved energy efficiency while maintaining good product yields. Such an approach is particularly valuable in medicinal chemistry, as it facilitates the rapid generation of structurally diverse analogs for structure–activity relationship (SAR) studies and lead optimization.

Molecular docking was used as an initial computational strategy to prioritize compounds for experimental evaluation. These studies showed that all compounds were favorably accommodated within the AChE active-site gorge, with compound **3n** exhibiting the most favorable binding affinity. The interaction profile indicated that the thiazol core and aryl substituents contribute to binding through π–π stacking with key aromatic residues (Tyr337, Phe338, Tyr341), hydrophobic interactions with Trp286, and hydrogen bonding with Tyr124. Notably, compound **3n** demonstrated a dual-site binding mode spanning both PAS and the aromatic gorge, consistent with the characteristics of potent AChE inhibitors.

Importantly, these computational predictions were supported by the subsequent in vitro Ellman assay, in which compound **3n** showed the lowest AChE IC_50_ (12.03 ± 0.17 μM), followed by compounds **3i** (22.32 ± 0.26 μM) and **3b** (29.51 ± 0.05 μM). Although these compounds were less potent than donepezil, the agreement between docking predictions and experimental enzyme inhibition supports the reliability of the proposed binding mode of this series. Nevertheless, molecular docking provides a static approximation of protein–ligand interactions, and future molecular dynamics simulations together with MM-GBSA binding free-energy calculations will be valuable for validating ligand stability and estimating binding free energies.

In addition, the selected thiazol derivatives exhibited favorable in silico pharmacokinetic profiles. All lead compounds complied with Lipinski’s and Veber’s rules, suggesting favorable drug-like properties. High predicted gastrointestinal absorption together with predicted blood–brain barrier permeability further supports their suitability as potential CNS drug candidates, which is an essential consideration for AD therapeutics. Furthermore, the predicted absence of CYP2D6 and CYP3A4 inhibition suggests a lower likelihood of clinically relevant drug–drug interactions. Although these computational predictions require experimental validation, they indicate that the synthesized derivatives possess physicochemical characteristics favorable for further preclinical development.

Network pharmacology analysis further suggested that the compounds may modulate multiple AD-related targets, including GSK3B, BACE1, PTGS2, ESR1, MAPK1, AChE, and MAP2K1, which are associated with amyloid processing, tau pathology, neuroinflammation, and oxidative stress, supporting the concept that the synthesized thiazol derivatives may function as MTDLs. Such a multi-target profile is especially appealing in AD, as the simultaneous modulation of multiple pathological mechanisms is increasingly recognized as a more effective therapeutic strategy than focusing on single-target intervention. However, these target interactions are based solely on computational predictions and hence further biochemical and molecular studies will be necessary to validate these predicted targets and determine their contribution to the observed neuroprotective effects.

OS and mitochondrial dysfunction are recognized as central contributors to neuronal death in AD and are closely associated with Aβ-induced neuro-toxicity, synaptic dysfunction, and neuronal apoptosis [91,92,93,94]. Although the 6-OHDA-induced SH-SY5Y cell model is more commonly employed to study Parkinsonian neurodegeneration, it is also widely used as a robust model of OS and mitochondrial injury [95,96,97,98]. Therefore, in the present study, this model was employed to evaluate the antioxidant and cytoprotective potential of the synthesized compounds rather than to recapitulate the complete pathological features of AD. Importantly, these findings were further complemented by evaluation in an Aβ42-expressing *Drosophila melanogaster* model, providing disease-relevant in vivo validation.

Biological evaluation in SH-SY5Y cells demonstrated that the selected compounds exhibited low cytotoxicity and provided significant neuroprotection against 6-OHDA-induced OS. Among these derivatives, compound **3n** exhibited the strongest neuroprotective efficacy by restoring cell viability, preserving normal cellular morphology, reducing intracellular ROS, and maintaining mitochondrial function, comparable to the reference drug donepezil. Collectively, these observations indicate that compound **3n** effectively mitigates OS-induced mitochondrial dysfunction, which is a hallmark of neurodegeneration implicated in both Alzheimer’s and Parkinson’s diseases.

The neuroprotective efficacy of the lead compound was further evaluated in an Aβ42-expressing *Drosophila melanogaster* model, which recapitulates several key pathological features of AD, including Aβ-induced neurodegeneration, progressive locomotor impairment, and reduced lifespan. Consistent with the in vitro findings, treatment with compound **3n** significantly improved locomotor performance (by approximately 20% compared to the untreated control; *p* ≤ 0.05) and moderately extended lifespan by approximately seven days. These findings demonstrate that the beneficial effects of compound **3n** extend beyond cellular antioxidant activity and translate into functional improvement in an in vivo model of AD. The Aβ42-expressing *Drosophila* model has become an established platform for the rapid evaluation of candidate neuroprotective compounds because it reproduces several conserved molecular and cellular mechanisms associated with human AD, including OS, mitochondrial and synaptic dysfunction, and Aβ-mediated neuronal toxicity [99,100,101,102]. Previous studies have shown that compounds capable of reducing OS and preserving mitochondrial homeostasis deliberately improved locomotor performance and lifespan in Aβ-expressing flies [103,104,105,106]. The comparable improvements observed with compound **3n** therefore support the hypothesis that the attenuation of OS contributes to its neuroprotective efficacy in vivo. Importantly, the in vivo performance of compound **3n** mirrors its favorable docking profile, AChE inhibitory activity, cytoprotective effects, the reduction in intracellular ROS, and the preservation of mitochondrial membrane potential, suggesting that the compound possesses a consistent multifunctional neuroprotective profile across computational, biochemical, cellular, and organismal models.

The combined computational, biochemical, cellular, and in vivo findings provide valuable structure–activity relationship (SAR) insights into the present series of 2-(arylamino)thiazol-4(5H)-one derivatives (Supplementary Table S4). Among the synthesized analogs, biological activity was markedly influenced by both the nature and position of substituents on the aryl ring. Compound **3n**, bearing a 2,5-dimethylphenyl substituent, consistently exhibited the most favorable overall pharmacological profile, including the strongest predicted AChE binding affinity, the lowest AChE IC_50_, increased cytoprotective activity, the reduction in intracellular ROS, the preservation of mitochondrial membrane potential, and improved locomotor performance in the Aβ42-expressing *Drosophila* model. The activity of compound **3n** may be attributed to the 2,5-dimethyl substitution pattern, which appears to provide an advantageous balance between hydrophobic interactions and favorable ligand orientation within the AChE binding gorge. In contrast, the para-methyl derivative **3i** and the 2,6-dimethyl analog **3b** displayed intermediate activity despite possessing similar hydrophobic substituents, indicating that the spatial arrangement of methyl groups is an important determinant of biological activity. Likewise, mono-methyl-substituted derivatives (**3d** and **3m**) demonstrated comparatively lower neuroprotective activity during the initial screening, while most halogen- and methoxy-substituted analogs were comparatively less active, suggesting that both substituent identity and substitution pattern contribute to the multifunctional activity of this scaffold. These observations are consistent with recent medicinal chemistry studies of thiazol- and thiazolidinone-based AChE inhibitors, which likewise reported that hydrophobic aryl substituents and appropriate substitution patterns enhance ligand accommodation within the aromatic gorge through favorable π–π stacking and hydrophobic interactions with key aromatic residues [55,107,108,109]. However, unlike many previous studies that primarily evaluated enzyme inhibition, the present work demonstrates that the optimized substitution pattern in compound **3n** was associated not only with improved AChE inhibition but also with enhanced cytoprotection, antioxidant activity, mitochondrial protection, and in vivo neuroprotection, supporting its potential as a multifunctional lead compound for AD.

Collectively, these findings indicate that optimization of the aryl substitution pattern influences not only AChE binding but also the broader neuroprotective properties of this scaffold. The consistent superiority of compound **3n** across computational, biochemical, cellular, and in vivo evaluations identifies the 2,5-dimethylphenyl analog as a promising lead structure for further optimization toward multifunctional anti-Alzheimer agents.

Despite the encouraging findings, several limitations should be acknowledged. Although molecular docking predictions were supported by the in vitro Ellman assay, further molecular dynamics (MD) simulations and MM-GBSA binding free-energy calculations would provide a more comprehensive understanding of ligand stability and protein–ligand interactions. Moreover, the biological evaluation was limited to a 6-OHDA-induced SH-SY5Y oxidative stress model and an Aβ42-expressing *Drosophila melanogaster* model, which do not fully recapitulate the complexity of human AD. In addition, AChE inhibition was evaluated using *Electrophorus electricus* AChE, and future studies employing recombinant human AChE and butyrylcholinesterase (BChE), together with validation in mammalian models, will be important to establish the translational potential of these compounds. Finally, the pharmacokinetic and network pharmacology analyses were based on computational predictions and therefore require experimental confirmation. Collectively, these findings establish the 2-(arylamino)thiazol-4(5H)-one scaffold as a promising template for further optimization and continued preclinical investigation toward the development of multifunctional disease-modifying therapeutics for AD.

## 4. Materials and Methods

### 4.1. Reagents and Chemicals

Dulbecco’s Modified Eagle Medium (DMEM), fetal bovine serum (FBS), penicillin–streptomycin solution, 0.25% trypsin–EDTA solution, and 0.4% trypan blue solution were sourced from Gibco (Thermo Fisher Scientific, Waltham, MA, USA). Sodium bicarbonate (NaHCO_3_), sodium chloride (NaCl), potassium chloride (KCl), disodium hydrogen phosphate (Na_2_HPO_4_), and potassium dihydrogen phosphate (KH_2_PO_4_) were obtained from HiMedia Laboratories (Mumbai, India). Acetylcholinesterase (AChE) from *Electrophorus electricus* (electric eel; Type VI-S, lyophilized powder, 200–1000 U/mg protein; Cat. No. C3389-500UN), 5,5′-dithiobis(2-nitrobenzoic acid) (DTNB; ≥98%, BioReagent grade; Cat. No. D8130-1G), and acetylthiocholine iodide (ATCI; ≥99.0%; Cat. No. 01480-1G) were purchased from Sigma-Aldrich (St. Louis, MO, USA). Donepezil hydrochloride (≥98% purity; Cat. No. HY-B0034; CAS No. 120011-70-3) was purchased from MedChemExpress (Monmouth Junction, NJ, USA). 3-(4,5-Dimethylthiazol-2-yl)-2,5-diphenyltetrazolium bromide (MTT) and dimethyl sulfoxide (DMSO) were purchased from Sigma-Aldrich (St. Louis, MO, USA). 2′,7′-Dichlorodihydrofluorescein diacetate (H_2_DCFDA; catalog number: HY-126793) and rhodamine 123 (catalog number: HY-101894) were procured from MedChemExpress (Monmouth Junction, NJ, USA). 6-Hydroxydopamine (6-OHDA) was kindly provided by collaborators at REVA University, Bengaluru, India, originally sourced from Sigma-Aldrich (St. Louis, MO, USA).

### 4.2. Synthesis of 2-(Arylamino)thiazol-4(5H)-one Derivatives

Commercially available reagents were used without further purification. All reactions were conducted in oven-dried glassware under controlled atmospheric conditions. Reaction progress was monitored by thin-layer chromatography (TLC) on Merck silica gel 60 F254 plates under UV illumination. Column chromatography was performed with silica gel (60–120 mesh) using a hexane–ethyl acetate mixture as the eluent. ^1^H and ^13^C NMR spectra were recorded on a Jeol ECZ 400R spectrometer (JEOL Ltd., Tokyo, Japan) in CDCl_3_ with tetramethylsilane (TMS) as the internal standard. High-resolution mass spectrometric analysis was carried out in a Waters-Xevo G2-XS-QToF mass spectrometer (Merck KgaA, Darmstadt, Germany, Milford, CT, USA). Microwave-assisted reactions were performed in a *Monowave 200 instrument* (Anton Paar) using closed vials, with the reaction temperature monitored by an inbuilt infrared (IR) sensor.

General procedure for the synthesis of 2-chloro-N-aryl acetamides 2(a–p)

To a pre-cooled solution of substituted aniline (1.0 mmol, 0.095 g) and triethylamine (TEA, 1.0 mmol, 0.11 g) in dichloromethane (10 mL), 2-chloroacetyl chloride (1.0 mmol, 0.12 g) was added dropwise. The mixture was stirred at room temperature for 10 min, and the progress of the reaction was monitored by TLC. Upon completion, the solvent was evaporated under reduced pressure and the residue was washed successively with water and hexane, then dried to afford the amide derivative in good yield.

General procedure for the synthesis of 2-(arylamino)thiazol-4(5H)-ones 3(a–p)

A mixture of 2-chloro-*N*-aryl acetamide (1.0 mmol, 0.18 g) and potassium thiocyanate (KSCN, 1.2 mmol, 0.12 g) in ethanol–water (1:1, 10 mL) mixture was transferred into a 10 mL sealed pressure vial and irradiated in a microwave reactor at 80 °C for 5 min. Reaction progress was monitored by TLC. After completion, the reaction mixture was cooled to room temperature and poured into ice-cold water. The resulting solid was filtered and purified by column chromatography on silica gel (60–120 mesh) using a petroleum ether-ethyl acetate mixture (80:20) as the eluent, yielding the desired thiazol derivatives in excellent yields.

Spectral data of synthesized compounds (**3a**–**3p**)

2-(phenylamino)thiazol-4(5*H*)-one (**3a**)

White solid; Yield: 0.184g, 0.95 mmol, 96%; ^1^H NMR (400 MHz, DMSO) δ 11.16 (s, 1H, NH), 7.69 (s, 1H, Ar), 7.37 (s, 2H, Ar), 7.16 (s, 1H, Ar), 6.99 (s, 1H, Ar), 4.01 (s, 2H, CH_2_). ^13^C NMR (100 MHz, DMSO) δ 188.65, 178.67, 129.50, 125.14, 121.98, 120.71, 35.30; ESI-HRMS: C_9_H_8_N_2_OS [M+H]^+^; Calculated: 193.0357; Found: 193.0429 (Supplementary Figures S1–S3).

2-((2,6-dimethylphenyl)amino)thiazol-4(5*H*)-one (**3b**)

White solid; Yield: 0.204g, 0.92 mmol, 93%; ^1^H NMR (400 MHz, DMSO) δ 9.20 (s, 1H, NH), 7.23 (d, *J* = 6.5 Hz, 1H, Ar), 7.17 (s, 1H, Ar), 7.06 (s, 1H, Ar), 4.27 (s, 2H, CH_2_), 2.03 (s, 6H, 2xCH_3_). ^13^C NMR (100 MHz, DMSO) δ 171.53, 157.47, 136.52, 133.72, 129.29, 128.60, 34.04, 17.64; ESI-HRMS: C_11_H_12_N_2_OS [M+H]^+^; Calculated: 221.0670; Found: 221.0742 (Supplementary Figures S4–S6).

2-((4-chlorophenyl)amino)thiazol-4(5*H*)-one (**3c**)

White solid; Yield: 0.215g, 0.94 mmol, 95%; ^1^H NMR (400 MHz, DMSO) δ 11.28 (s, 1H, NH), 7.73 (s, 1H, Ar), 7.44 (s, 1H, Ar), 7.39 (s, 1H, Ar), 6.97 (s, 1H, Ar), 4.03 (s, 2H, CH_2_). ^13^C NMR (100 MHz, DMSO) δ 188.57, 178.79, 129.68, 128.87, 123.57, 122.24, 34.97; ESI-HRMS: C_9_H_7_ClN_2_OS [M+H]^+^; Calculated: 226.9968; Found: 227.0041(Supplementary Figures S7–S9).

2-(*m*-tolylamino)thiazol-4(5*H*)-one (**3d**)

White solid; Yield: 0.191g, 0.92 mmol, 93%; ^1^H NMR (400 MHz, DMSO) δ 11.08 (s, 1H, NH), 7.51 (d, *J* = 7.3 Hz, 1H, Ar), 7.25 (s, 1H, Ar), 6.97 (s, 1H, Ar), 6.81 (s, 1H, Ar), 4.00 (s, 2H, CH_2_), 2.31 (s, 3H, CH_3_). ^13^C NMR (100 MHz, DMSO) δ 188.68, 178.58, 139.19, 129.33, 125.88, 122.58, 121.11, 117.93, 35.40, 21.63; ESI-HRMS: C_10_H_10_N_2_OS [M+H]^+^; Calculated: 207.0514; Found: 207.0587 (Supplementary Figures S10–S12).

2-((4-bromophenyl)amino)thiazol-4(5*H*)-one (**3e**)

White solid; Yield: 0.256g, 0.94 mmol, 95%; ^1^H NMR (400 MHz, DMSO) δ 11.27 (s, 1H, NH), 7.68 (d, *J* = 5.6 Hz, 1H, Ar), 7.58 (s, 1H, Ar), 7.52 (s, 1H, Ar), 6.91 (s, 1H, Ar), 4.02 (s, 2H, CH_2_). ^13^C NMR (100 MHz, DMSO) δ 188.57, 178.80, 132.59, 123.97, 122.57, 117.02, 34.94; ESI-HRMS: C_9_H_7_BrN_2_OS [M+H]^+^; Calculated: 269.9462; Found: 270.9536 (Supplementary Figures S13–S15).

2-((2-fluorophenyl)amino)thiazol-4(5*H*)-one (**3f**)

White solid; Yield: 0.197g, 0.93 mmol, 94%; ^1^H NMR (400 MHz, DMSO) δ 11.99 (s, 1H, NH), 7.24 (d, *J* = 4.2 Hz, 1H, Ar), 7.16 (d, *J* = 4.1 Hz, 2H, Ar), 7.04 (d, *J* = 4.2 Hz, 1H, Ar), 4.02 (s, 2H, CH_2_). ^13^C NMR (100 MHz, DMSO) δ 174.73, 155.30, 152.88, 136.00, 126.14, 125.30, 123.73, 116.57, 34.89; ESI-HRMS: C_9_H_7_FN_2_OS [M+H]^+^; Calculated: 211.0263; Found: 211.0336 (Supplementary Figures S16–S18).

2-((3,5-dichlorophenyl)amino)thiazol-4(5*H*)-one (**3g**)

White solid; Yield: 0.247g, 0.94 mmol, 95%; ^1^H NMR (400 MHz, DMSO) δ 9.40 (s, 1H, NH), 7.70 (d, *J* = 2.2 Hz, 1H, Ar), 7.46 (s, 1H, Ar), 7.00 (s, 1H, Ar), 4.12 (s, 2H, CH_2_). ^13^C NMR (100 MHz, DMSO) δ 171.76, 158.21, 134.88, 128.35, 124.06, 120.54, 34.32; ESI-HRMS: C_9_H_6_Cl_2_N_2_OS [M+H]^+^; Calculated: 260.9578; Found: 260.9645 (Supplementary Figure S19–S21).

2-((4-fluorophenyl)amino)thiazol-4(5*H*)-one (**3h**)

White solid; Yield: 0.197g, 0.93 mmol, 94%; ^1^H NMR (400 MHz, DMSO) δ 9.29 (s, 1H, NH), 7.72 (d, *J* = 3.7 Hz, 1H, Ar), 7.31 (s, 1H, Ar), 7.21 (d, *J* = 4.0 Hz, 1H, Ar), 7.01 (d, *J* = 3.9 Hz, 1H, Ar), 4.13 (s, 2H, CH_2_). ^13^C NMR (100 MHz, DMSO) δ 188.53, 178.68, 131.27, 123.77, 122.69, 116.32, 34.09; ESI-HRMS: C_9_H_7_FN_2_OS [M+H]^+^; Calculated: 211.0263; Found: 211.0336 (Supplementary Figures S22–S24).

2-(*p*-tolylamino)thiazol-4(5*H*)-one (**3i**)

White solid; Yield: 0.194g, 0.94 mmol, 95%; ^1^H NMR (400 MHz, DMSO) δ 11.08 (s, 1H, NH), 7.57 (s, 1H, Ar), 7.18 (d, *J* = 2.4 Hz, 2H, Ar), 6.91 (s, 1H, Ar), 3.99 (s, 2H, CH_2_), 2.28 (s, 3H, CH_3_). ^13^C NMR (100 MHz, DMSO) δ 188.59, 178.27, 134.34, 129.86, 128.77, 120.64, 35.64, 20.94; ESI-HRMS: C_10_H_10_N_2_OS [M+H]; Calculated: 207.0514; Found: 207.0581 (Supplementary Figures S25–S27).

2-((3-chlorophenyl)amino)thiazol-4(5*H*)-one (**3j**)

White solid; Yield: 0.210 g, 0.92 mmol, 93%; ^1^H NMR (400 MHz, DMSO) δ 11.86 (s, 1H, NH), 7.96 (s, 1H, Ar), 7.39 (d, *J* = 1.4 Hz, 1H, Ar), 7.20 (s, 1H, Ar), 6.93 (d, *J* = 1.5 Hz, 1H, Ar), 4.00 (s, 2H, CH_2_). ^13^C NMR (100 MHz, DMSO) δ 188.60, 179.12, 133.87, 131.40, 124.60, 121.49, 120.49, 119.05, 34.84; ESI-HRMS: C_9_H_7_ClN_2_OS [M+H]^+^; Calculated: 226.9968; Found: 227.0040 (Supplementary Figures S28–S30).

2-((2-chlorophenyl)amino)thiazol-4(5*H*)-one (**3k**)

White solid; Yield: 0.214g, 0.94 mmol, 95%; ^1^H NMR (400 MHz, DMSO) δ 12.00 (s, 1H, NH), 7.49 (d, *J* = 7.1 Hz, 1H, Ar), 7.30 (d, *J* = 6.1 Hz, 1H, Ar), 7.13 (d, *J* = 7.1 Hz, 1H, Ar), 7.02 (s, 1H, Ar), 4.02 (s, 2H, CH_2_). ^13^C NMR (100 MHz, DMSO) δ 174.44, 145.75, 130.32, 128.46, 126.07, 122.94, 34.75; ESI-HRMS: C_9_H_7_ClN_2_OS [M+H]^+^; Calculated: 226.9968; Found: 227.0040 (Supplementary Figures S31–S33).

2-((2,3-dichlorophenyl)amino)thiazol-4(5*H*)-one (**3l**)

White solid; Yield: 0.247g, 0.94 mmol, 95%; ^1^H NMR (400 MHz, DMSO) δ 12.09 (s, 1H, NH), 7.38 (d, *J* = 4.0 Hz, 1H, Ar), 7.33 (s, 1H, Ar), 7.02 (d, *J* = 4.2 Hz, 1H, Ar), 4.05 (s, 2H, CH_2_). ^13^C NMR (100 MHz, DMSO) δ 174.20, 159.15, 147.99, 132.72, 129.03, 126.29, 124.54, 121.44, 34.84; ESI-HRMS: C_9_H_6_Cl_2_N_2_OS [M-H]^+^; Calculated: 259.9578; Found: 260.9645 (Supplementary Figures S34–S36).

2-(*o*-tolylamino)thiazol-4(5*H*)-one (**3m**)

White solid; Yield: 0.191g, 0.92 mmol, 93%; ^1^H NMR (400 MHz, DMSO) δ 11.70 (s, 1H, NH), 7.22 (s, 1H, Ar), 7.16 (d, *J* = 4.5 Hz, 1H, Ar), 7.06 (d, *J* = 4.7 Hz, 1H, Ar), 6.87 (s, 1H, Ar), 3.94 (s, 2H, CH_2_), 2.13 (s, 3H, CH_3_). ^13^C NMR (100 MHz, DMSO) δ 176.54, 171.11, 145.68, 131.01, 130.63, 127.11, 125.39, 121.75, 35.32, 17.87; ESI-HRMS: C_10_H_10_N_2_OS [M-H]; Calculated: 207.0514; Found: 207.0586 (Supplementary Figures S37–S39).

2-((2,4-dimethylphenyl)amino)thiazol-4(5*H*)-one (**3n**)

White solid; Yield: 0.202g, 0.91 mmol, 92%; ^1^H NMR (400 MHz, DMSO) δ 11.58 (s, 1H, NH), 7.07 (d, *J* = 5.8 Hz, 1H, Ar), 6.99 (d, *J* = 5.8 Hz, 1H, Ar), 6.81 (d, *J* = 6.0 Hz, 1H, Ar), 3.92 (s, 2H, CH_2_), 2.25 (s, 3H, CH_3_), 2.10 (s, 3H, CH_3_). ^13^C NMR (100 MHz, DMSO) δ 188.17, 177.96, 141.91, 134.89, 131.69, 131.00, 127.58, 122.36, 35.81, 20.95, 17.81; ESI-HRMS: C_11_H_12_N_2_OS [M+H]^+^; Calculated: 221.0670; Found: 221.0742 (Supplementary Figures S40–S42).

2-((4-methoxyphenyl)amino)thiazol-4(5*H*)-one (**3o**)

White solid; Yield: 0.206g, 0.92 mmol, 93%; ^1^H NMR (400 MHz, DMSO) δ 11.04 (s, 1H, NH), 7.60 (d, *J* = 9.0 Hz, 2H, Ar), 6.99 (s, 1H, Ar), 6.94 (s, 1H, Ar), 3.99 (s, 2H, CH_2_), 3.75 (s, 3H, OCH_3_). ^13^C NMR (100 MHz, DMSO) δ 188.47, 177.97, 132.50, 122.27, 114.98, 114.57, 55.75, 36.09; ESI-HRMS: C_10_H_10_N_2_O_2_S [M-H]^+^; Calculated: 223.0536; Found: 223.0536 (Supplementary Figures S43–S45).

2-((2-methoxyphenyl)amino)thiazol-4(5*H*)-one (**3p**)

White solid; Yield: 0.206g, 0.92 mmol, 93%; ^1^H NMR (400 MHz, DMSO) δ 7.80 (s, 1H, Ar), 7.17 (s, 1H, Ar), 7.05 (d, *J* = 8.4 Hz, 1H, Ar), 6.94 (s, 1H, Ar), 3.93 (s, 2H, CH_2_), 3.75 (s, 3H, OCH_3_). ^13^C NMR (100 MHz, DMSO) δ 177.55, 152.21, 134.76, 130.82, 126.80, 123.47, 121.08, 112.66, 55.90, 35.67; ESI-MS: C_10_H_10_N_2_O_2_S [M-H]^+^; Calculated: 223.0536; Found: 223.0536 (Supplementary Figures S46–S48).

### 4.3. Molecular Docking and Binding Interaction Analysis

Molecular docking was conducted to predict the binding interactions of synthesized 2-(arylamino)thiazol-4(5H)-one derivatives within the active-site gorge of acetylcholinesterase (AChE), using the crystal structure of human AChE complexed with donepezil (PDB ID: 4EY7) obtained from the Protein Data Bank. Docking simulations were performed using the SwissDock web server, implementing AutoDock Vina (version 1.2.5) [110].

For receptor preparation, only chain A was retained, while the co-crystallized ligand (donepezil; residue ID E20), crystallographic water molecules, and non-essential heteroatoms, including N-acetyl-D-glucosamine (NAG) and α-L-fucose (FUC), were removed. No additional modifications were made to the protein structure before docking. To validate the docking protocol, the co-crystallized ligand donepezil was re-docked into the prepared receptor. The predicted pose closely matched the crystallographic conformation, yielding a root mean square deviation (RMSD) of 0.43 Å, thereby confirming the reliability of the docking protocol.

Ligand structures were generated from SMILES notation and submitted to the SwissDock server. The submitted ligand structures corresponded to the 4(5H)-keto (lactam) tautomer of the thiazol-4(5H)-one scaffold, which is generally regarded as the predominant thermodynamically stable form under physiological conditions. Protonation states and partial atomic charges required for docking were assigned automatically using the default ligand preparation workflow implemented in SwissDock**.** The docking search space was centered at coordinates (−13, −47, 26 Å) using a cubic grid box of 20 × 20 × 20 Å encompassing both the catalytic active site (CAS) and peripheral anionic site (PAS). The exhaustiveness parameter was set to 20 to improve sampling, while all remaining parameters were kept at their default values. Multiple binding poses were generated for each ligand and ranked according to predicted binding free energy (ΔG, kcal mol^−1^), with the lowest-energy pose selected for subsequent interaction analysis.

Protein–ligand interaction analyses for the top-ranked docked complexes were conducted using the Protein–Ligand Interaction Profiler (PLIP) web server [111]. Docked structures obtained from SwissDock served as input to identify critical interactions such as hydrogen bonds, hydrophobic contacts, π–π stacking, and other non-covalent interactions between the ligands and AChE active-site residues.

PLIP automatically calculated interaction distances and residue-specific contacts, enabling the detailed characterization of binding patterns for the selected compounds. Both detailed zoomed-in views of interactions and overall ligand binding poses were generated to depict ligand orientation within the AChE active site. A schematic diagram of the AChE active-site architecture was created based on established structural features to aid the interpretation of ligand binding regions, including the peripheral anionic site (PAS), aromatic gorge, and catalytic active site (CAS).

### 4.4. Determination of In Vitro AChE Inhibitory Activity

The AChE inhibitory activity of the compounds **3n**, **3i**, and **3b** was measured using Ellman’s colorimetric method [55,56,57]. This assay is based on the hydrolysis of acetylthiocholine iodide (ATCI) by AChE, followed by the reaction of the released thiocholine with 5,5′-dithiobis-(2-nitrobenzoic acid) (DTNB) to produce the yellow-colored 5-thio-2-nitrobenzoate anion, which was quantified spectrophotometrically at 412 nm. Donepezil hydrochloride was used as the reference inhibitor [55,56,57].

Stock solutions of the test compounds and donepezil hydrochloride were prepared in dimethyl sulfoxide (DMSO) and subsequently diluted with 50 mM Tris-HCl buffer (pH 8.0) to obtain the desired test concentrations (0.005 μM–30 μM), while maintaining the final DMSO concentration below 1% (*v*/*v*). Acetylcholinesterase from *Electrophorus electricus* was dissolved in 20 mM Tris-HCl buffer (pH 7.5) to obtain an enzyme stock solution (610 U/mL). The assay was performed in 96-well microplates with a final reaction volume of 200 μL per well. Each reaction mixture consisted of 150 μL of 50 mM Tris-HCl buffer (pH 8.0), 20 μL of AChE solution, 10 μL of the test compound or donepezil. The mixtures were pre-incubated for 15 min at room temperature. Subsequently, 10 μL of acetylthiocholine iodide (ATCI) and 10 μL of 3.33 mM DTNB solution were added to each well, and the absorbance was immediately measured at 412 nm using a microplate reader. Wells containing DMSO without inhibitor served as the negative control (100% enzyme activity), whereas wells without enzyme served as blanks. Results are expressed as mean ± SEM from triplicate experiments and inhibitory activity was expressed as the IC_50_ value, defined as the inhibitor concentration producing a 50% reduction in enzyme activity.

The percentage inhibition of AChE activity was calculated relative to the vehicle control according to the following equation:

$$\%\;Inhibition\;=\;\left(1\;-\;\frac{\mathrm{A\_sample}\;-\;\mathrm{A\_blank}}{\mathrm{A\_control}\;-\;\mathrm{A\_blank}}\right)\;\times \;100$$

### 4.5. In Silico Pharmacokinetics and Target Prediction

Physicochemical properties, including molecular weight (MW), number of rotatable bonds, and topological polar surface area (TPSA), alongside pharmacokinetic parameters such as absorption, distribution, metabolism, were predicted using the SwissADME web tool (http://www.swissadme.ch, accessed on 16 April 2026). Compound structures in SMILES format served as input data for these predictions. Drug-likeness was evaluated based on Lipinski’s rule of five and Veber’s rule. Compliance with at least three Lipinski criteria—molecular weight ≤ 500 g/mol, lipophilicity (LogP) between −2 and 5, hydrogen bond acceptors (N or O) ≤ 10, and hydrogen bond donors (NH or OH) ≤ 5—was required. Veber’s rule further considered TPSA ≤ 140 Å^2^ and rotatable bonds ≤ 10 [112,113]. Additional pharmacokinetic parameters, including gastrointestinal absorption, BBB permeability, P-glycoprotein substrate prediction, and cytochrome P450 inhibition profiles, were also obtained from SwissADME.

Potential molecular targets of the compounds were predicted using their SMILES representations in two web-based platforms: SwissTargetPrediction (http://www.swisstargetprediction.ch, accessed on 30 April 2026) [114] and the Similarity Ensemble Approach (SEA) database (https://sea.bkslab.org/, accessed on 1 May 2026) [115]. AD-related targets were compiled by querying GeneCards (https://www.genecards.org/, accessed on 1 May 2026) [116] and DisGeNET (https://www.disgenet.org/, accessed on 1 May 2026) [117]. Overlapping targets between the compounds’ predicted targets and AD-associated genes were identified through Venn diagram analysis. Target prediction was performed using Homo sapiens as the target organism. A compound–target interaction network was constructed and visualized using the Cytoscape software (version 3.10.1). Protein–protein interaction (PPI) networks were generated via the STRING database, followed by network topology analysis to identify key targets based on degree centrality, defined by the number of direct connections per node [118].

### 4.6. In Vitro Studies

#### 4.6.1. Culture of SH-SY5Y Cells

Neuroblastoma SH-SY5Y cells obtained from National Center for Cell Science (NCCS, Pune, India) were maintained in DMEM with 10% heat-inactivated FBS and 1% penicillin–streptomycin at 37 °C in humidified air containing 5% carbon dioxide.

#### 4.6.2. Cytotoxicity of 2-(Arylamino)thiazol-4(5H)-one Derivatives on SH-SY5Y Cell Line

The cytotoxicity of 2-(arylamino)thiazol-4(5H)-one derivatives was assessed using SH-SY5Y cells. Cells were seeded in 96-well microtiter plates (Tarson) at a density of 30,000–50,000 cells per well and incubated for 24 h at 37 °C in a humidified atmosphere containing 5% CO_2_. Following this, varying concentrations of the derivatives and DMSO (negative control) were added, and cells were incubated for an additional 24 h. MTT reagent (0.5 mg/mL in medium) was then applied for 4 h to stain viable cells [119]. Formazan crystals formed were dissolved by shaking with DMSO, and absorbance was measured at 570 nm using a Synergy H1 microplate reader (BioTek, Winooski, VT, USA). The IC_50_ values were calculated as the concentration of each compound required to inhibit cell growth by 50%.

#### 4.6.3. Determination of SH-SY5Y Cell Viability by MTT Assay

SH-SY5Y neuronal cells were treated with varying concentrations (1–10 μM) of 2-(arylamino)thiazol-4(5H)-one derivatives for 24 h to evaluate cytotoxicity. For neuroprotective assessment, cells were pretreated with the same concentration range of derivatives for 4 h, followed by exposure to 100 μM 6-OHDA for an additional 24 h. Cell viability was determined by incubating with 0.5 mg/mL MTT reagent for 3 h at 37 °C, and absorbance was measured at 570 nm using a Synergy H1 microplate reader (BioTek). Viability at each concentration was expressed as a percentage relative to the untreated control group [120].

#### 4.6.4. Morphological Assessment by Bright-Field Microscopy

SH-SY5Y cells were seeded at a density of 1.0 × 10^5^ cells/mL in six-well plates and incubated at 37 °C in a humidified atmosphere containing 5% CO_2_. Following treatment as previously described, neuronal cell morphology was examined using an inverted light microscope (Lawrence Mayo) at 20× magnification [68].

#### 4.6.5. Measurement of ROS Production

Intracellular ROS production in SH-SY5Y neuronal cells was assessed using the fluorescent probe H_2_DCFDA. Cells were seeded at a density of 1.0 × 10^5^ cells/mL in 96-well plates and incubated for 24 h. Selected 2-(arylamino)thiazol-4(5H)-one derivatives were applied at 2.5 μM for 4 h as pretreatment, followed by exposure to 100 μM 6-OHDA for 24 h to induce neuro-toxicity. Subsequently, cells were incubated with 10 μM H_2_DCFDA for 30 min at 37 °C in the dark. Fluorescence was measured using a microplate reader (Synergy H1, BioTek) at excitation and emission wavelengths of 485 nm and 528 nm, respectively, and a fluorescence microscope (Zeiss). ROS levels were expressed as a percentage relative to the untreated control group [121].

#### 4.6.6. Measurement of Mitochondrial Membrane Potential by Rhodamine-123

Mitochondrial membrane potential (MMP) was assessed using the mitochondrial-specific fluorescent dye rhodamine-123. SH-SY5Y cells were seeded overnight at 1.0 × 10^5^ cells/mL in 96-well plates, followed by pretreatment with 2-(arylamino)thiazol-4(5H)-one derivatives for 4 h and subsequent exposure to 100 μM 6-OHDA for 24 h. Rhodamine-123 was then added at a final concentration of 10 μM, and the plates were incubated for 30 min in the dark. After incubation, cells were washed twice with phosphate-buffered saline (PBS), and MMP levels were measured using a microplate reader (Synergy H1, BioTek) at excitation and emission wavelengths of 488 nm and 525 nm, respectively [122].

All experiments were performed in three independent biological replicates, and data are presented as mean ± SEM.

### 4.7. In Vivo Studies

#### 4.7.1. Fly Stocks and Husbandry

*Drosophila melanogaster* were maintained at 25 °C under 12 h light/dark cycles on a standard yeast cornmeal diet. The fly strains used included BL#458, BL#33774, and Canton-S, all obtained from the Bloomington Stock Center. For experimental crosses, Canton-S wild-type flies were mated with flies carrying GAL4 transgenes, and heterozygous GAL4/+ progeny served as controls. The pan-neuronal driver Elav-Gal4 was employed to drive expression. Virgin female Elav-Gal4 flies were crossed with male UAS-APP.Abeta42 (BL#33774) or Canton-S wild-type males [123]. After eclosion, three-day-old male progeny were fed varying concentrations of selected thiazol derivatives. For the climbing assay, flies were maintained on standard food supplemented with compounds **3n**, **3i**, or **3b** at final concentrations of 10, 20, or 30 μM. For the longevity assay, flies were maintained on food supplemented with 20 μM of each compound throughout the experimental period. Fresh drug-containing food was provided every three days. The experimental workflow is summarized in Supplementary Figure S58.

#### 4.7.2. Climbing Assay

The negative geotaxis assay was conducted following the method described by Sun et al. [124]. Male flies collected within three days post-eclosion were randomly divided into groups of 20 flies per vial (five biological replicates per treatment group; *n* = 100 flies) and maintained on standard food containing compounds **3n**, **3i**, or **3b** (10, 20, or 30 μM). Climbing performance was evaluated on days 7, 14, 21, and 28 following continuous drug treatment. Every 7 days, flies were transferred to empty vials marked with genotype and height scales (up to 8 cm) and allowed to acclimate for 10 min. Flies were then gently tapped to the bottom, and the distance climbed within 10 s was recorded. Each fly group underwent 10 trials, with a 5 min rest interval between trials. The number of flies crossing a line of 8 cm within 10 s was counted and expressed as a percentage, representing climbing performance. Data were analyzed using two-way ANOVA with Dunnett’s multiple comparisons.

#### 4.7.3. Survival Assay

The longevity assay was conducted over 70 days using twenty (n = 100) newly eclosed male and female flies (1:1 sex ratio) per group. Each treatment group consisted of five biological replicates of 20 flies each (*n* = 100 flies). Flies were maintained on standard food supplemented with 20 μM of compounds **3n**, **3i**, or **3b**, while control groups received vehicle-treated food. Flies were transferred to fresh medium, with or without the drug, every three days and maintained at 25 °C. Mortality was recorded every two days, and surviving flies were moved to fresh food vials accordingly [125]. Lifespan data were analyzed and presented as Kaplan–Meier survival curves.

### 4.8. Statistical Analysis

All quantitative data are presented as mean ± standard error of the mean (SEM) from at least three independent experiments, unless otherwise stated. Statistical analyses were performed using GraphPad Prism version 10.3.1 (GraphPad Software, San Diego, CA, USA). Comparisons among multiple groups in cell-based assays were performed using one-way analysis of variance (ANOVA) followed by Dunnett’s multiple-comparison test. Climbing assay data were analyzed using two-way ANOVA followed by Dunnett’s multiple-comparison test, whereas lifespan data were presented as Kaplan–Meier survival curves. Cytotoxicity IC_50_ values were calculated by nonlinear regression analysis and were reported with 95% confidence intervals (95% CI). Differences were considered statistically significant at *p* < 0.05.

## 5. Conclusions

A series of 2-(arylamino)thiazol-4(5H)-one derivatives was synthesized and evaluated for their neuroprotective activity against 6-OHDA-induced neuronal damage in SH-SY5Y cells and an Aβ42-induced *Drosophila* model of AD. Among these, three derivatives (**3n**, **3i**, and **3b**) exhibited low cytotoxicity alongside significant neuroprotection, demonstrated by increased cell viability, reduced ROS production, and the preservation of MMP. Notably, compound **3n** showed the most favorable neuroprotective activity and was effective in mitigating Aβ42-associated pathological phenotypes in vivo. Molecular docking studies predicted that these compounds possess acetylcholinesterase (AChE) inhibitory potential, with favorable binding interactions within the enzyme’s active site, and these computational predictions were supported by in vitro AChE inhibition studies. Target prediction further identified seven core proteins, including GSK3B, PTGS2, BACE1, ESR1, MAPK1, AChE, and MAP2K1, implicated in AD pathogenesis and related pathways including OS, neuroinflammation, and synaptic dysfunction. In silico pharmacokinetic analyses predicted that these derivatives comply with Lipinski’s and Veber’s rules, indicating favorable drug-like properties. Collectively, these findings identify compound **3n** as the lead derivative in this series, exhibiting a consistent profile across computational prediction, experimental AChE inhibition, cellular neuroprotection, and in vivo efficacy. Although further studies, including evaluation in mammalian AD models together with detailed pharmacokinetic, blood–brain barrier permeability, toxicity, and mechanistic investigations, are required, the present study highlights 2-(arylamino)thiazol-4(5H)-one derivatives as promising multifunctional lead scaffolds for further optimization and preclinihcal evaluation as potential therapeutics for AD.

## Figures and Tables

**Table 1:** Synthesized 2-(arylamino)thiazol-4(5H)-ones (**3a**–**3p**).

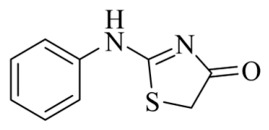	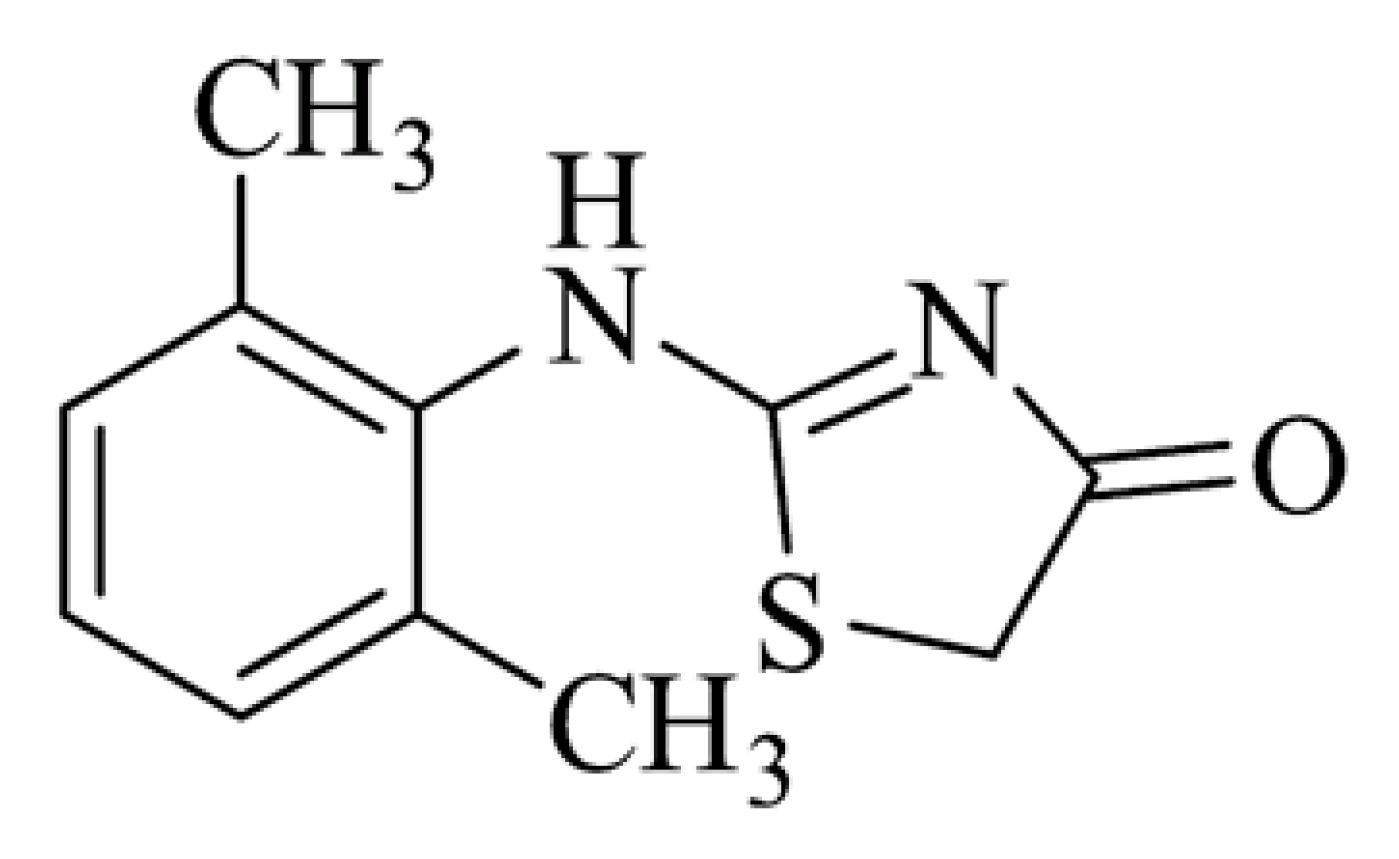	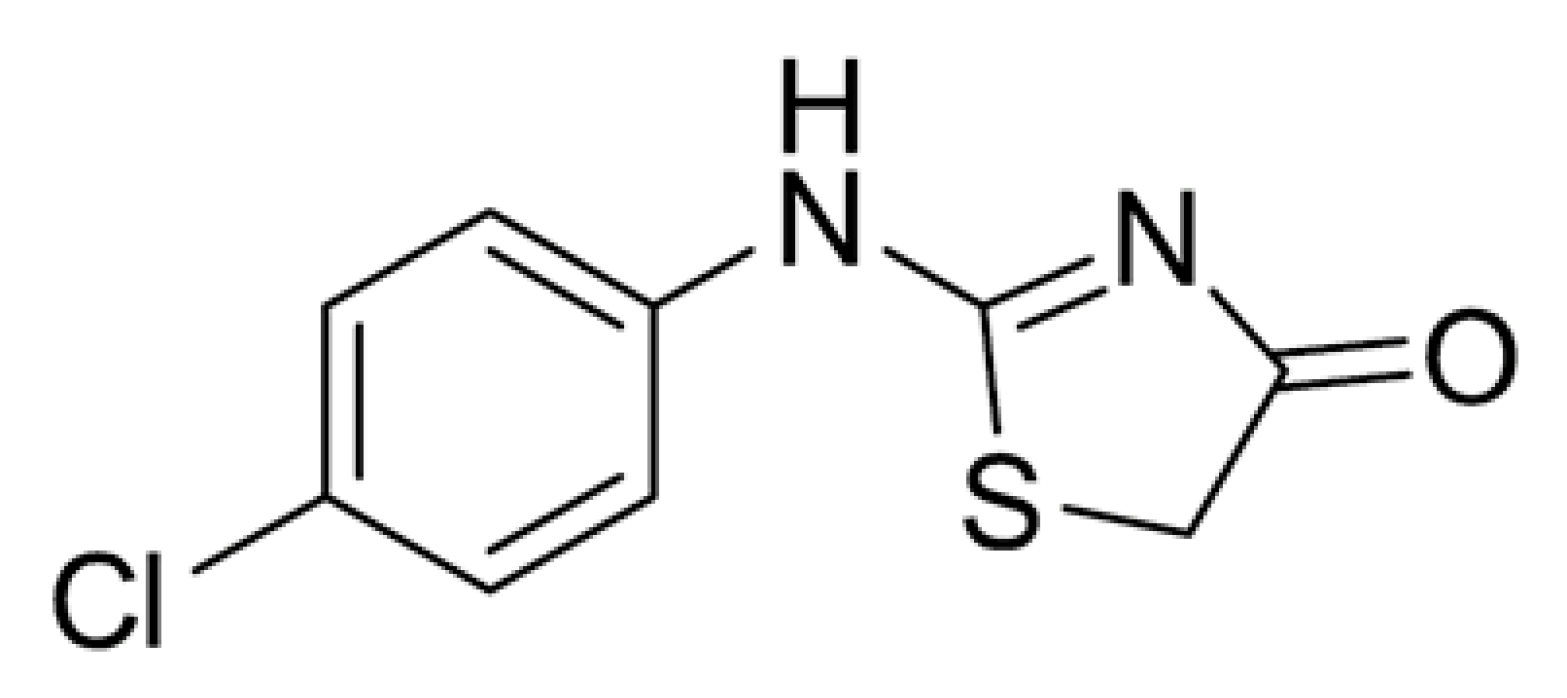	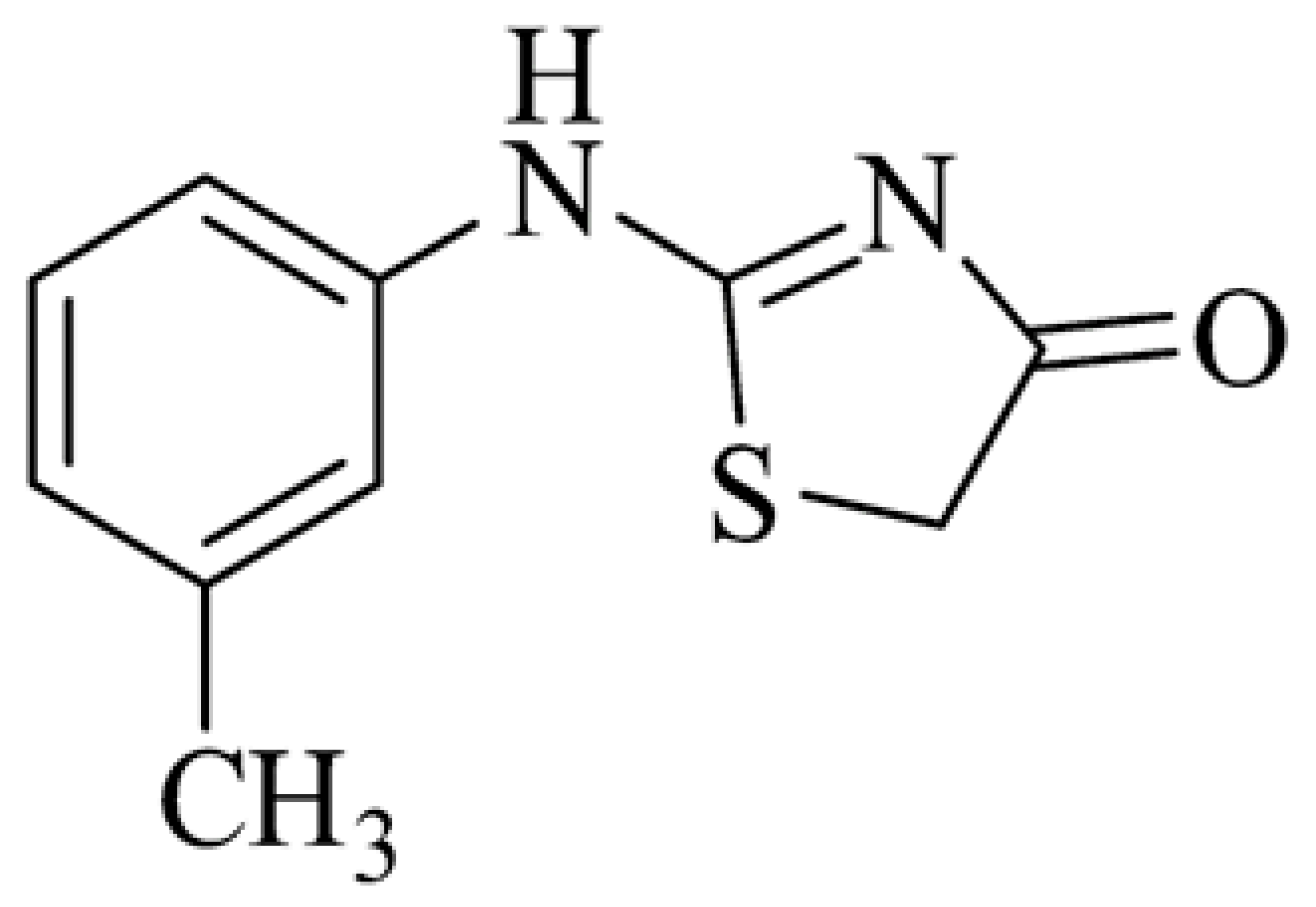
**3a** 4 min: 96% yield	**3b** 4 min: 95% yield	**3c** 4 min: 93% yield	**3d** 4 min: 95% yield
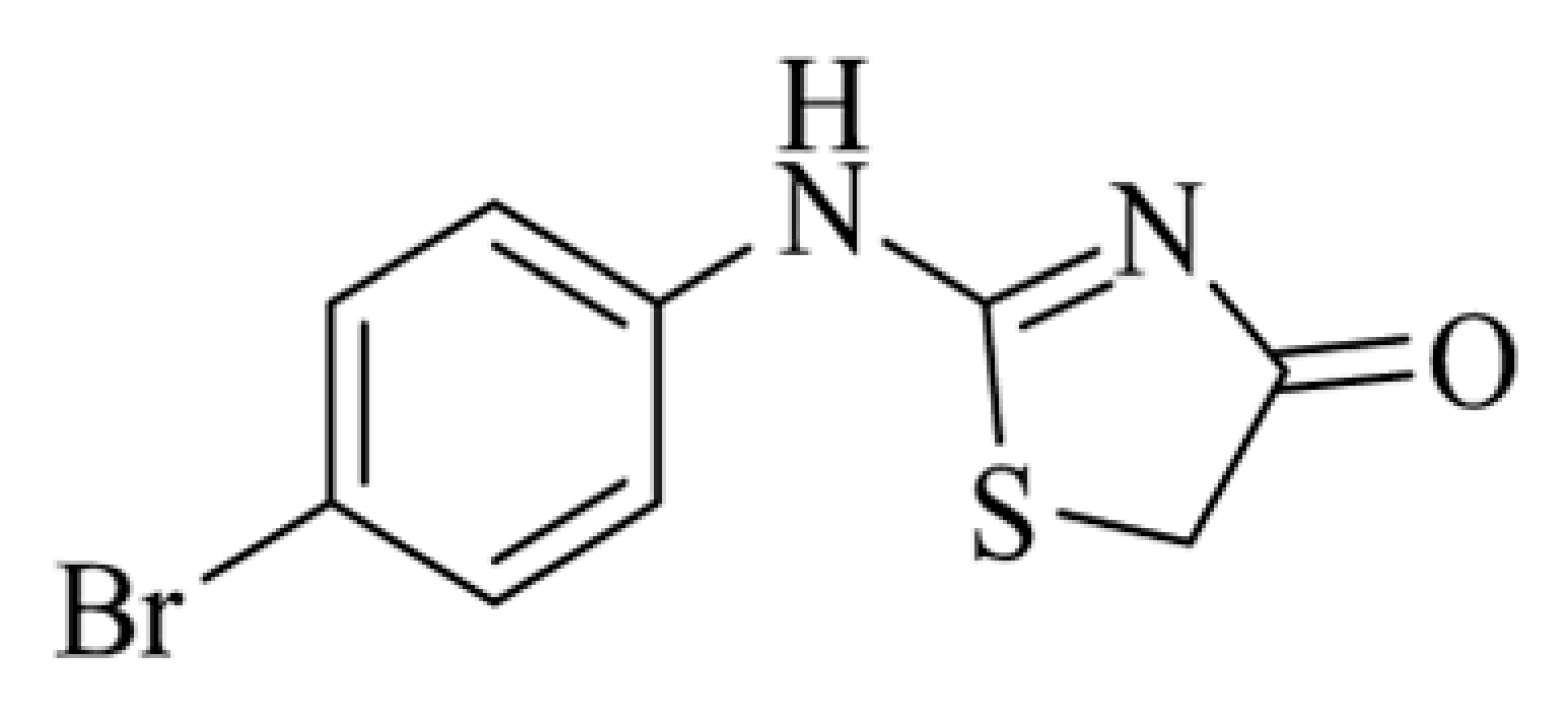	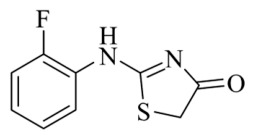	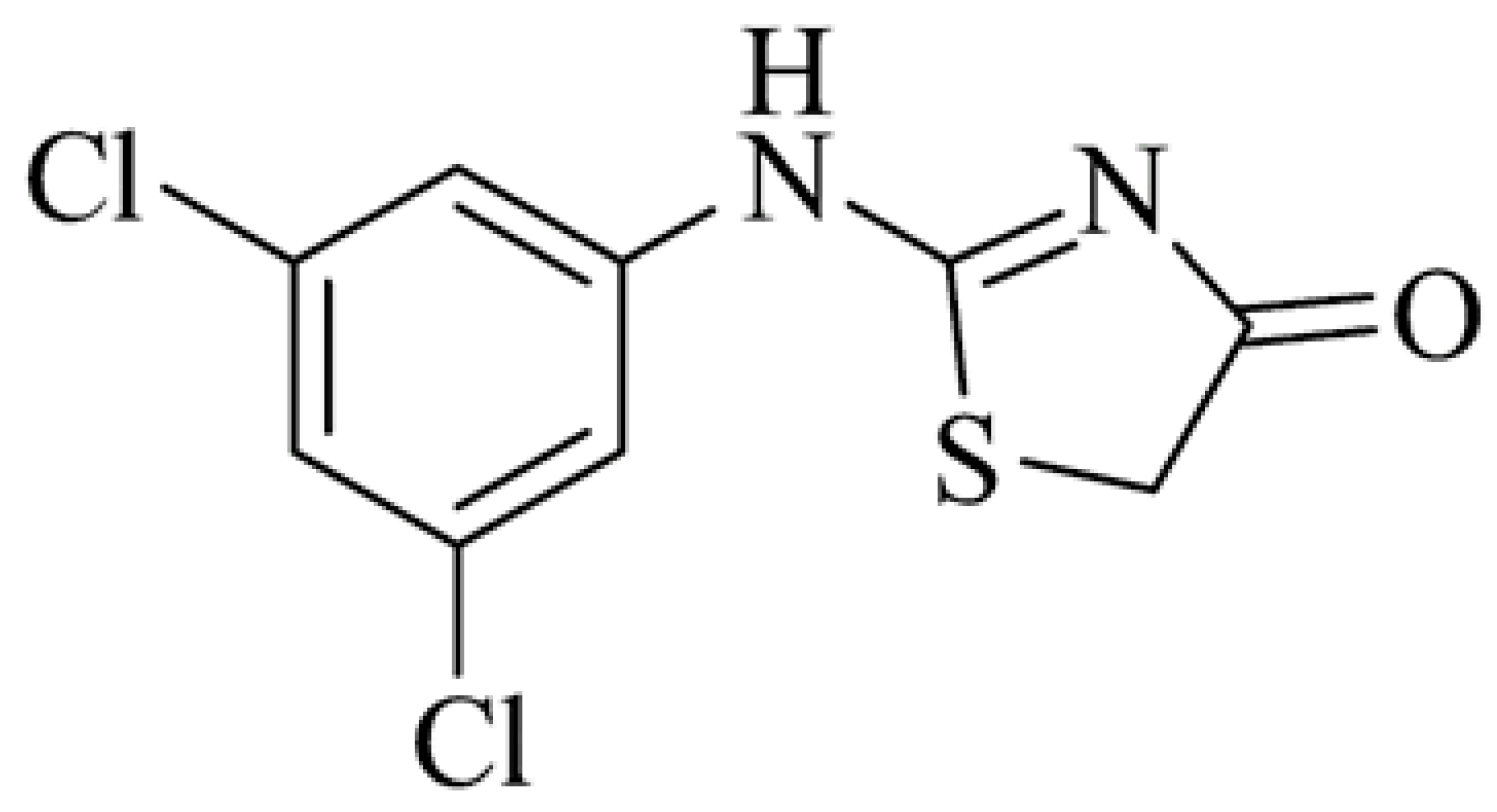	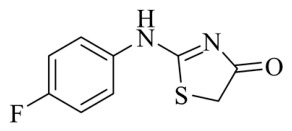
**3e** 5 min: 93% yield	**3f** 5 min: 93% yield	**3g** 5 min: 95% yield	**3h** 4 min: 94% yield
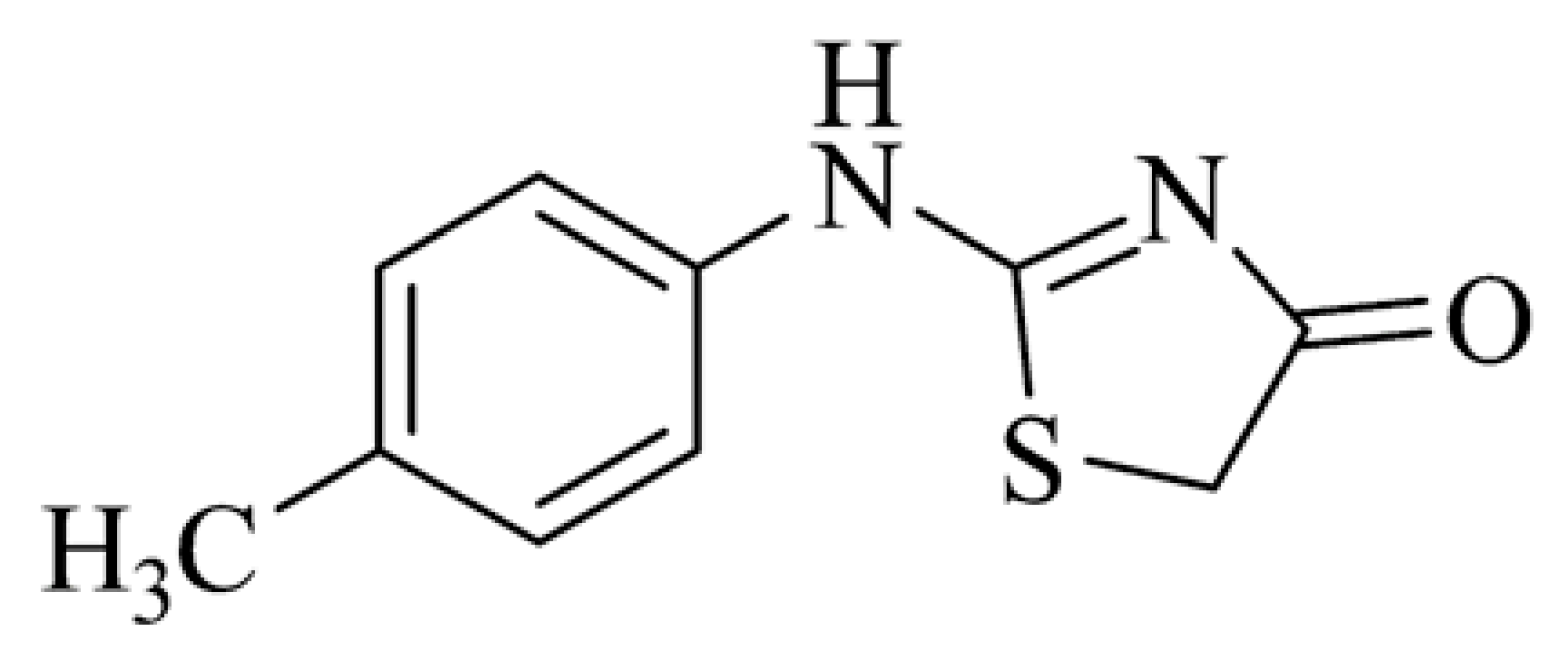	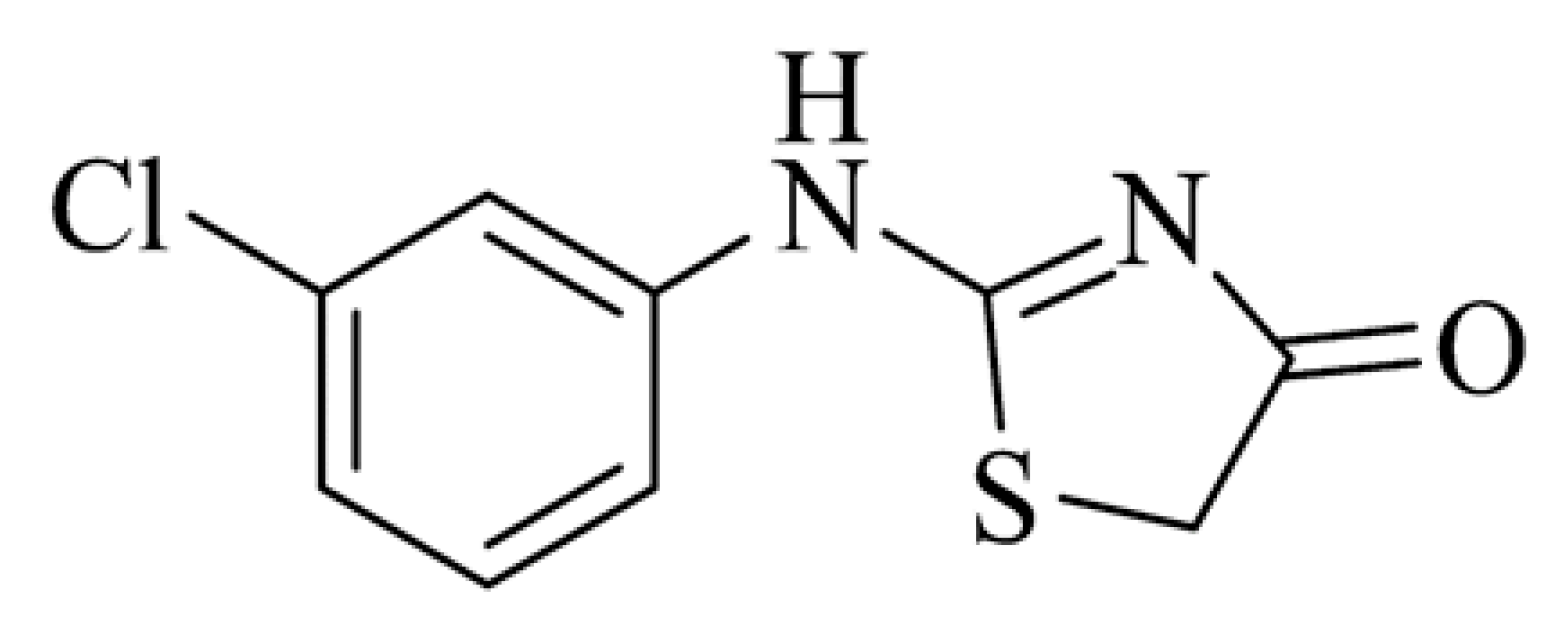	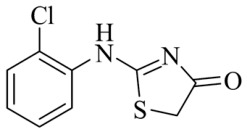	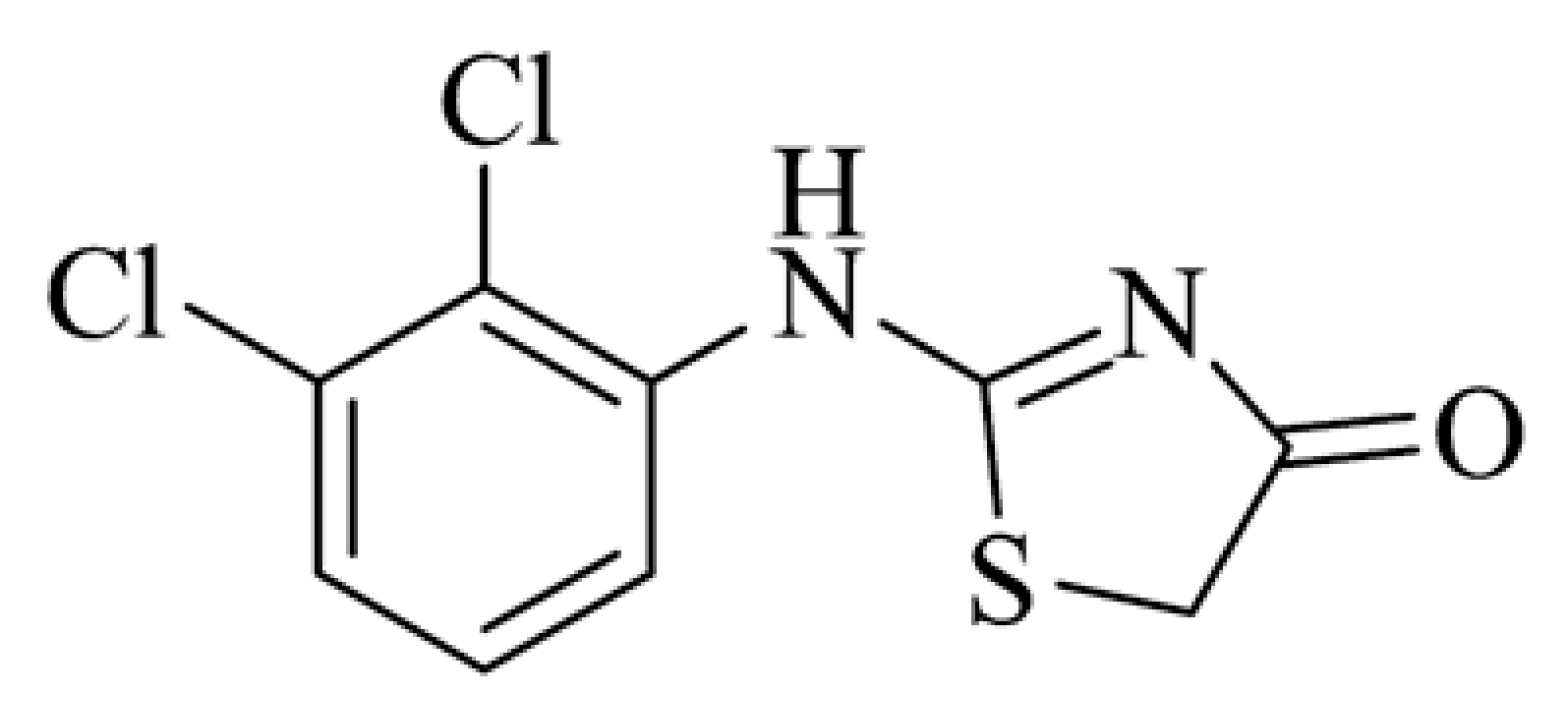
**3i** 4 min: 94% yield	**3j** 4 min: 95% yield	**3k** 5 min: 93% yield	**3l** 5 min: 93% yield
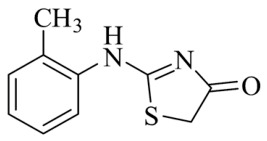	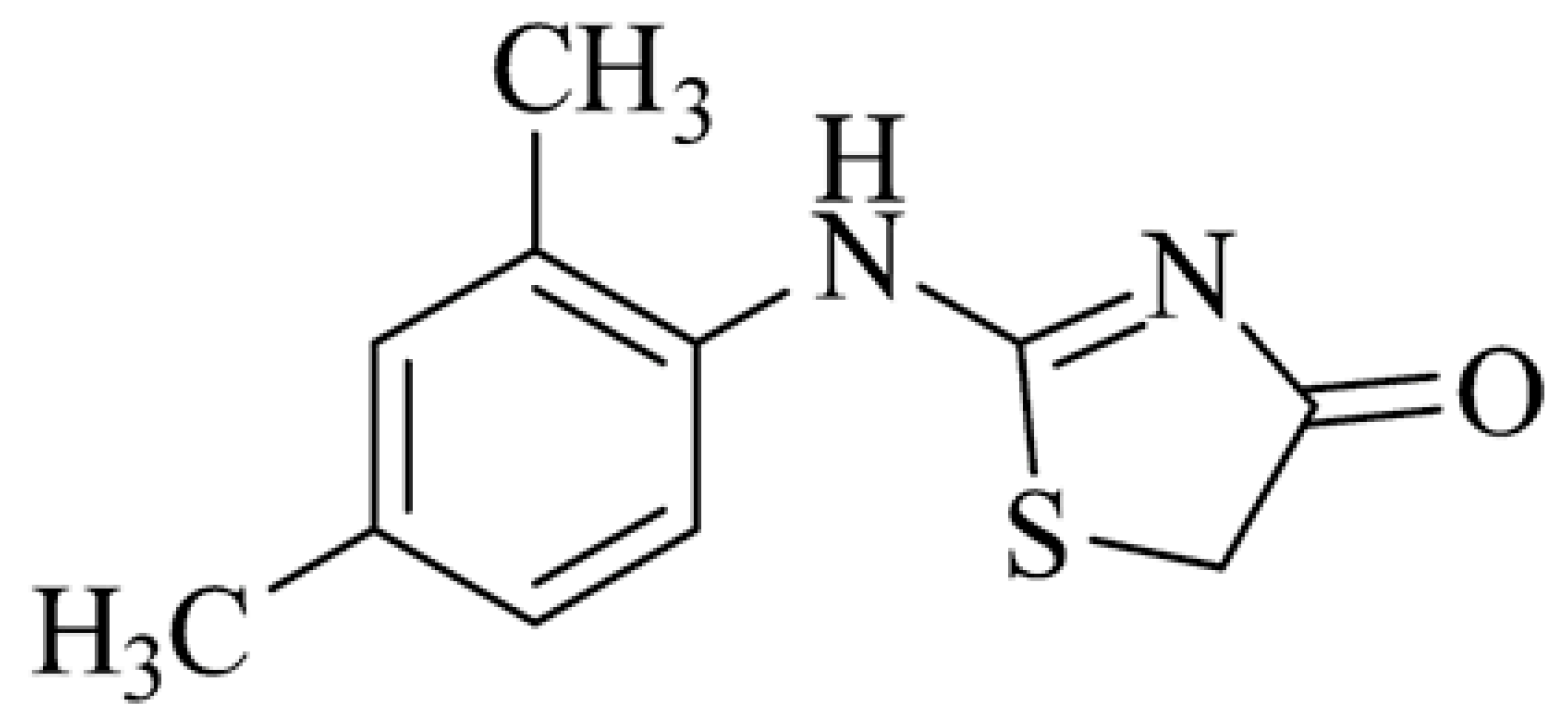	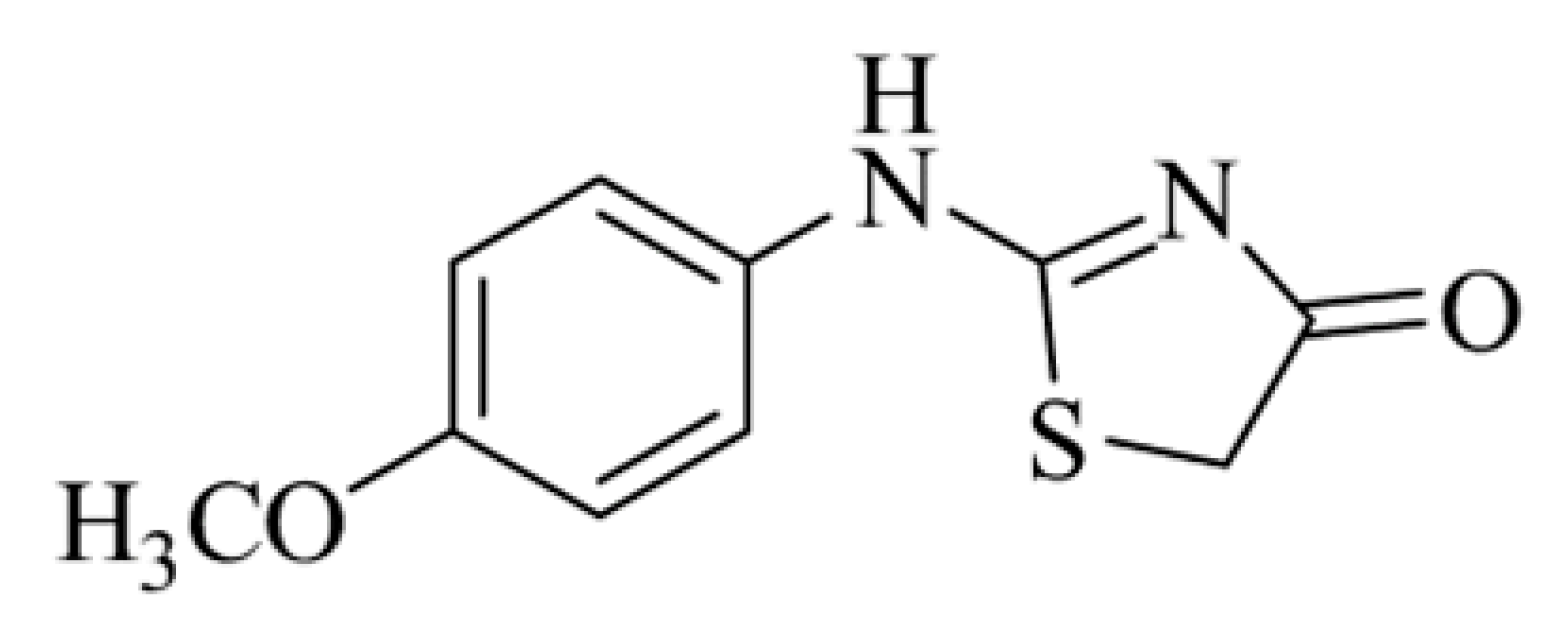	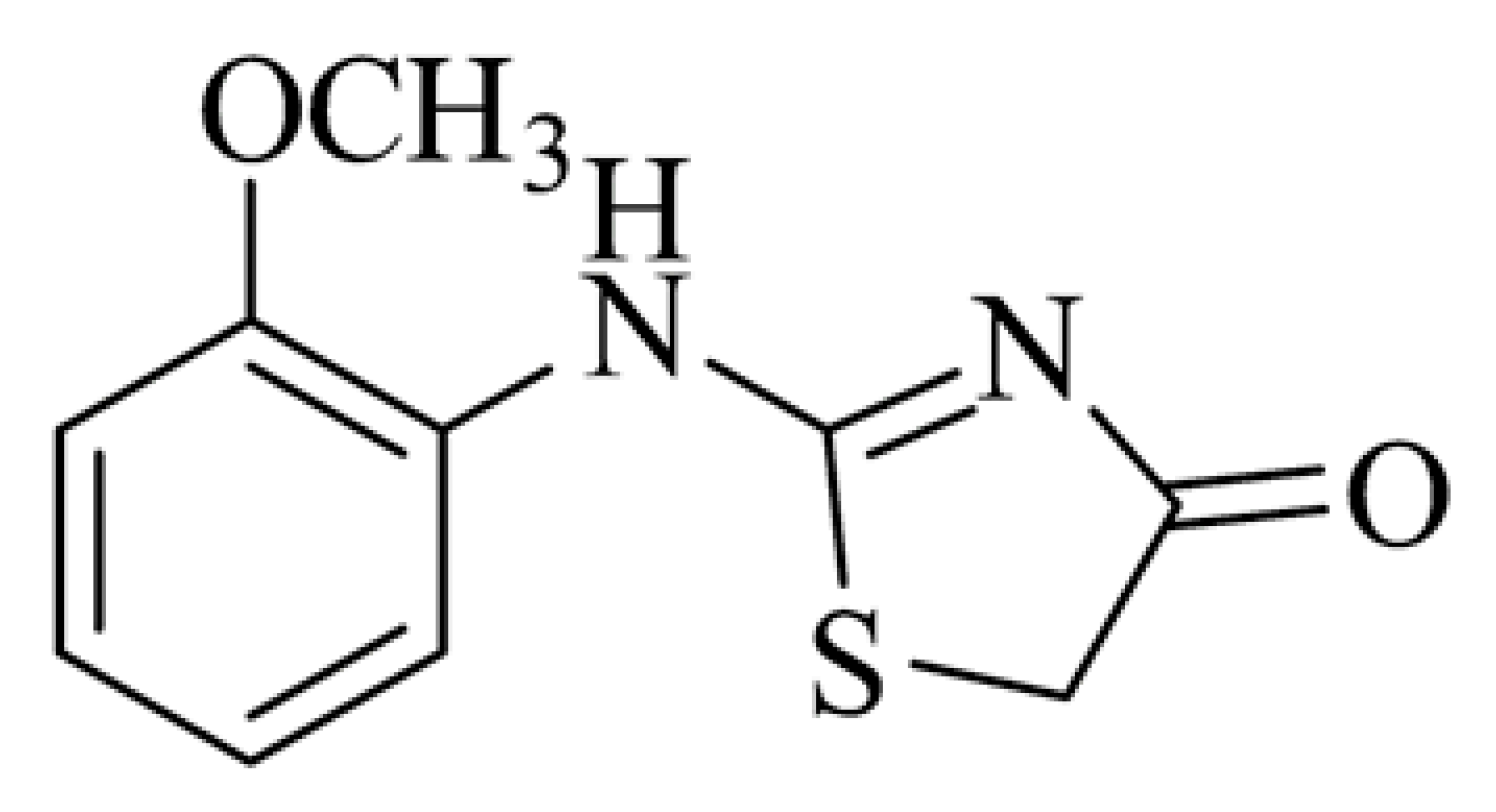
**3m** 5 min: 92% yield	**3n** 5 min: 93% yield	**3o** 4 min: 95% yield	**3p** 4 min: 95% yield

2-Chloro-*N*-arylacetamide (1.0 mmol), KSCN (1.0 mmol), ethanol–water [1:1] (10 mL/ mmol), MWI (80 °C).

**Table 2:** Molecular docking binding energies against acetylcholinesterase (AChE; PDB ID: 4EY7) and in vitro AChE inhibitory activity of donepezil and the selected compounds (**3n**, **3i**, and **3b**) against *Electrophorus electricus* acetylcholinesterase (EeAChE). Lower binding energy values indicate favorable predicted binding affinity. IC_50_ values are presented as mean ± SEM (N = 3).

Compound	Binding Energy (kcal/mol)	AChE (IC_50_)
Donepezil	−11.765	54.6± 1.5 nM
**3n**	−8.638	12.03 ± 0.17 μM
**3i**	−7.971	22.32 ± 0.26 μM
**3b**	−7.943	29.51 ± 0.05 μM

**Table 3:** Predicted physicochemical characteristics, pharmacokinetic parameters and drug-likeness of the selected compounds (**3n**, **3i**, and **3b**).

Compounds	3n	3i	3b	Donepezil
**Physicochemical properties**				
Molecular weight (g/mol)	220.29	207.05	221.07	379.49
Rotatable bonds	2	2	2	6
H-bond acceptors	2	2	2	4
H-bond donors	1	1	1	0
TPSA	66.76	66.76	66.76	38.77
**Absorption**				
Lipophilicity (iLOGP)	2.20	1.85	2.08	3.92
Water solubility	Moderately Soluble	Soluble	Moderately Soluble	Moderately Soluble
GI absorption	High	High	High	High
BBB permeant	Yes	Yes	Yes	Yes
**Metabolism**				
CYP1A2 inhibitor	Yes	Yes	Yes	No
CYP2D6 inhibitor	No	No	No	Yes
CYP3A4 inhibitor	No	No	No	Yes
**Drug-likeness**				
Lipinski’s rule	Yes	Yes	Yes	Yes
Veber’s rule	Yes	Yes	Yes	Yes

**Table 4:** Core target information in the PPI Network of 2-(arylamino) thiazol-4(5H)-one derivatives.

No	Target Name	Abbreviation	UniProtID	DC	BC	CC
1	Glycogen Synthase Kinase 3 Beta	GSK3B	P49841	18	0.254	0.846
2	Prostaglandin-Endoperoxide Synthase 2	PTGS2	P35354	16	0.159	0.785
3	Beta-Secretase 1	BACE1	P56817	12	0.061	0.687
4	Estrogen Receptor 1	ESR1	P03372	12	0.106	0.687
5	Mitogen-Activated Protein Kinase 1	MAPK1	P28482	10	0.015	0.647
6	Acetylcholinesterase	ACHE	P22303	10	0.086	0.647
7	Mitogen-Activated Protein Kinase Kinase 1	MAP2K1	Q02750	10	0.02	0.647

DC, degree centrality; BC, betweenness centrality; CC, closeness centrality.

**Table 5:** Cytotoxicity of 2-(arylamino)thiazol-4(5H)-one derivatives against neuroblastoma SH-SY5Y cell line. IC_50_ values were calculated by nonlinear regression (GraphPad Prism) and are presented with 95% confidence intervals (CIs).

Compound	IC_50_ (in µM)	95% CI
**3a**	23.85	23.07–25.00
**3b**	18.07	17.37–18.98
**3c**	21.46	20.23–23.33
**3d**	24.11	22.52–26.44
**3e**	23.73	22.99–24.82
**3f**	26.17	25.14–28.00
**3g**	26.08	25.12–27.75
**3h**	25.85	24.91–27.44
**3i**	18.45	17.65–19.53
**3j**	23.40	22.64–24.52
**3k**	26.37	25.26–28.47
**3l**	18.38	17.63–19.37
**3m**	23.52	21.68–26.87
**3n**	18.59	17.89–19.51
**3o**	23.21	22.25–24.76
**3p**	24.08	23.29–25.25

## Data Availability

Data are contained within the article and Supplementary Materials.

## Supplementary Materials

The following supporting information can be downloaded at https://www.mdpi.com/article/10.3390/ijms27156642/s1.

## Abbreviations

The following abbreviations are used in this manuscript:

NDD	Neurodegenerative diseases
OS	Oxidative stress
ROS	Reactive oxygen species
6-OHDA	6-hydroxydopamine
AD	Alzheimer’ disease
AChE	Acetylcholinesterase
PPI	Protein–protein interaction
