# Supplementary material for: 2-(Arylamino)thiazol-4(5H)-ones Mitigate Oxidative Stress and Confer Neuroprotection in Cellular and Drosophila Models of Alzheimer’s Disease
Source: Int J Mol Sci. 2026 Jul 25;27(15):6642. doi: 10.3390/ijms27156642 (PMC13465329; doi:10.3390/ijms27156642)
Supplement: Supplementary file 1 [file ijms-27-06642-s001.zip › ijms-4333874-supplementary.pdf]

2-(Arylamino) thiazol-4(5H)-ones Mitigate Oxidative Stress and Confer Neuroprotection in Cellular and Drosophila Models of Alzheimer's Disease

Supplementary Materials

S1. Spectral characterization of synthesized compounds

Contents	Page No.
Figure S1: ^{1}H NMR of 2-(phenylamino)thiazol-4(5*H*)-one (3a)	3
Figure S2: ^{13}C NMR of 2-(phenylamino)thiazol-4(5*H*)-one (3a)	3
Figure S3: ESI-HRMS of 2-(phenylamino)thiazol-4(5*H*)-one (3a)	4
Figure S4: ^{1}H NMR of 2-((2,6-dimethylphenyl)amino)thiazol-4(5*H*)-one (3b)	4
Figure S5: ^{13}C NMR of 2-((2,6-dimethylphenyl)amino)thiazol-4(5*H*)-one (3b)	5
Figure S6 ESI-HRMS of 2-((2,6-dimethylphenyl) amino)thiazol-4(5*H*)-one (3b)	5
Figure S7: ^{1}H NMR of 2-((4-chlorophenyl)amino)thiazol-4(5*H*)-one (3c)	6
Figure S8: ^{13}C NMR of 2-((4-chlorophenyl)amino)thiazol-4(5*H*)-one (3c)	6
Figure S9: ESI-HRMS of 2-((4-chlorophenyl)amino)thiazol-4(5*H*)-one (3c)	7
Figure S10: ^{1}H NMR of 2-(*m*-tolylamino))thiazol-4(5*H*)-one (3d)	7
Figure S11: ^{13}C NMR of 2-(*m*-tolylamino))thiazol-4(5*H*)-one (3d)	8
Figure S12: ESI-HRMS of 2-(*m*-tolylamino))thiazol-4(5*H*)-one (3d)	8
Figure S13: ^{1}H NMR of 2-((4-bromophenyl) amino)thiazol-4(5*H*)-one (3e)	9
Figure S14: ^{13}C NMR of 2-((4-bromophenyl) amino)thiazol-4(5*H*)-one (3e)	9
Figure S15: ESI-HRMS of 2-((4-bromophenyl) amino)thiazol-4(5*H*)-one (3e)	10
Figure S16: ^{1}H NMR of 2-((2-fluorophenyl)) amino)thiazol-4(5*H*)-one (3f)	10
Figure S17: ^{13}C NMR of 2-((2-fluorophenyl) amino)thiazol-4(5*H*)-one (3f)	11
Figure S18: ESI-HRMS of 2-((2-fluorophenyl)) amino)thiazol-4(5*H*)-one (3f)	11
Figure S19: ^{1}H NMR of 2-((3,5-dichlorophenyl) amino)thiazol-4(5*H*)-one (3g)	12
Figure S20: ^{13}C NMR of 2-((3,5-dichlorophenyl) amino)thiazol-4(5*H*)-one (3g)	12
Figure S21: ESI-HRMS of 2-((3,5-dichlorophenyl) amino)thiazol-4(5*H*)-one (3g)	13
Figure S22: ^{1}H NMR of 2-((4-fluorophenyl)amino)thiazol-4(5*H*)-one (3h)	13
Figure S23: ^{13}C NMR of 2-((4-fluorophenyl)amino)thiazol-4(5*H*)-one (3h)	14
Figure S24: ESI-HRMS of 2-((4-fluorophenyl)amino)thiazol-4(5*H*)-one (3h)	14
Figure S25: ^{1}H NMR of 2-(*p*-tolylamino)thiazol-4(5*H*)-one (3i)	15
Figure S26: ^{13}C NMR of 2-(*p*-tolylamino)thiazol-4(5*H*)-one (3i)	15
Figure S27: ESI-HRMS of 2--(*p*-tolylamino)thiazol-4(5*H*)-one (3i)	16
Figure S28: ^{1}H NMR of 2-((3-chlorophenyl)amino)thiazol-4(5H)-one (3j)	16
Figure S29: ^{13}C NMR of 2-((3-chlorophenyl)amino)thiazol-4(5*H*)-one (3j)	17
Figure S30: ESI-HRMS of 2-((3-chlorophenyl)amino)thiazol-4(5*H*)-one (3j)	17
Figure S31: ^{1}H NMR of 2-((2-chlorophenyl)amino)thiazol-4(5*H*)-one (3k)	18
Figure S32: ^{13}C NMR of 2-((2-chlorophenyl)amino)thiazol-4(5*H*)-one (3k)	18

Figure S33: ESI-HRMS of 2-((2-chlorophenyl)amino)thiazol-4(5*H*)-one (3k)	19
Figure S34: ^{1}H NMR of 2-((2,3-dichlorophenyl)amino)thiazol-4(5*H*)-one (3l)	19
Figure S35: ^{13}C NMR of 2-((2,3-dichlorophenyl)amino)thiazol-4(5*H*)-one (3l)	20
Figure S36: ESI-HRMS of 2-((2,3-dichlorophenyl)amino)thiazol-4(5*H*)-one (3l)	20
Figure S37: ^{1}H NMR of 2-(*o*-tolylamino)thiazol-4(5*H*)-one (3m)	21
Figure S38: ^{13}C NMR of 2-(*o*-tolylamino)thiazol-4(5*H*)-one (3m)	21
Figure S39: ESI-HRMS of 2-(*o*-tolylamino)thiazol-4(5*H*)-one (3m)	22
Figure S40: ^{1}H NMR of 2-((2,4-dimethylphenyl))amino)thiazol-4(5*H*)-one (3n)	22
Figure S41: ^{13}C NMR of 2-((2,4-dimethylphenyl)amino)thiazol-4(5*H*)-one (3n)	23
Figure S42: ESI-HRMS of 2-((2,4-dimethylphenyl)amino)thiazol-4(5*H*)-one (3n)	23
Figure S43: ^{1}H NMR of 2-(4-methoxyphenyl)amino)thiazol-4(5*H*)-one (3o)	24
Figure S44: ^{13}C NMR of 2-(4-methoxyphenyl)amino)thiazol-4(5*H*)-one (3o)	24
Figure S45: ESI-HRMS of 2-(4-methoxyphenyl)amino)thiazol-4(5*H*)-one (3o)	25
Figure S46: ^{1}H NMR of 2-(2-methoxyphenyl)amino)thiazol-4(5*H*)-one (3p)	25
Figure S47: ^{13}C NMR of 2-(2-methoxyphenyl)amino)thiazol-4(5*H*)-one (3p)	26
Figure S48: ESI-HRMS of 2-(2-methoxyphenyl)amino)thiazol-4(5*H*)-one (3p)	26

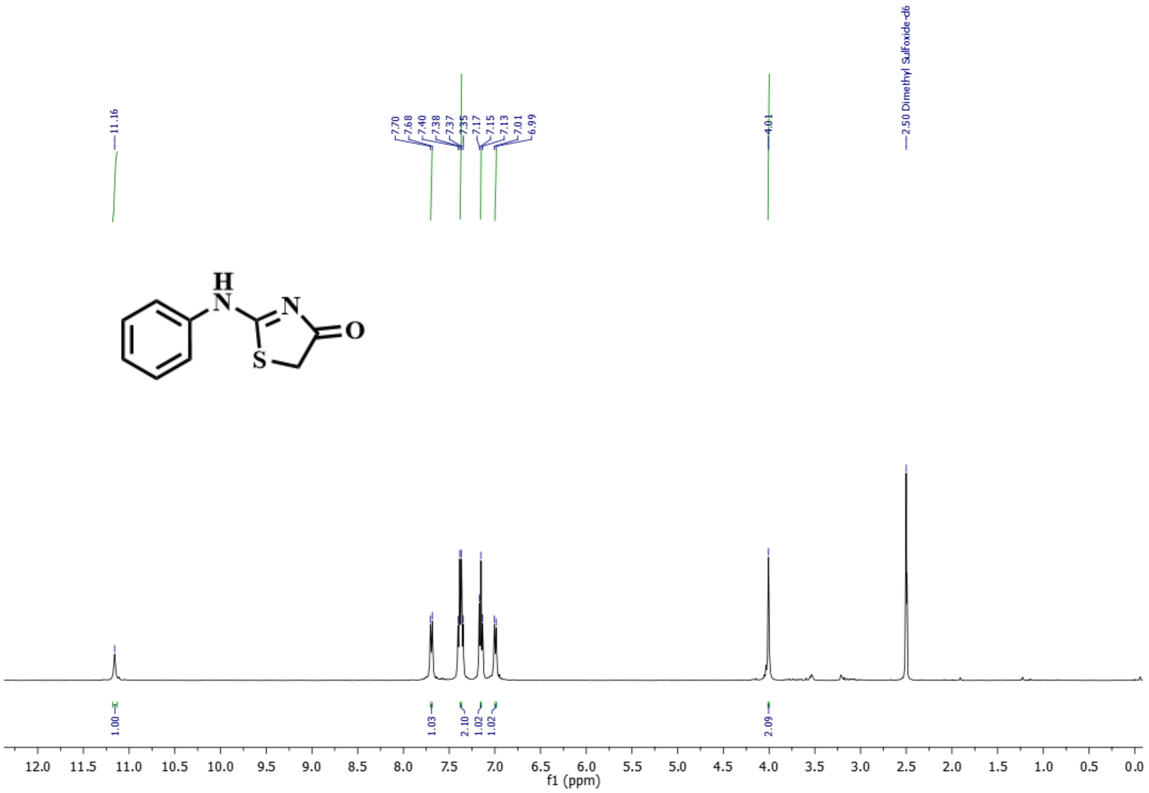

Figure S1: ^{1}H NMR of 2-(phenylamino)thiazol-4(5*H*)-one (3a)

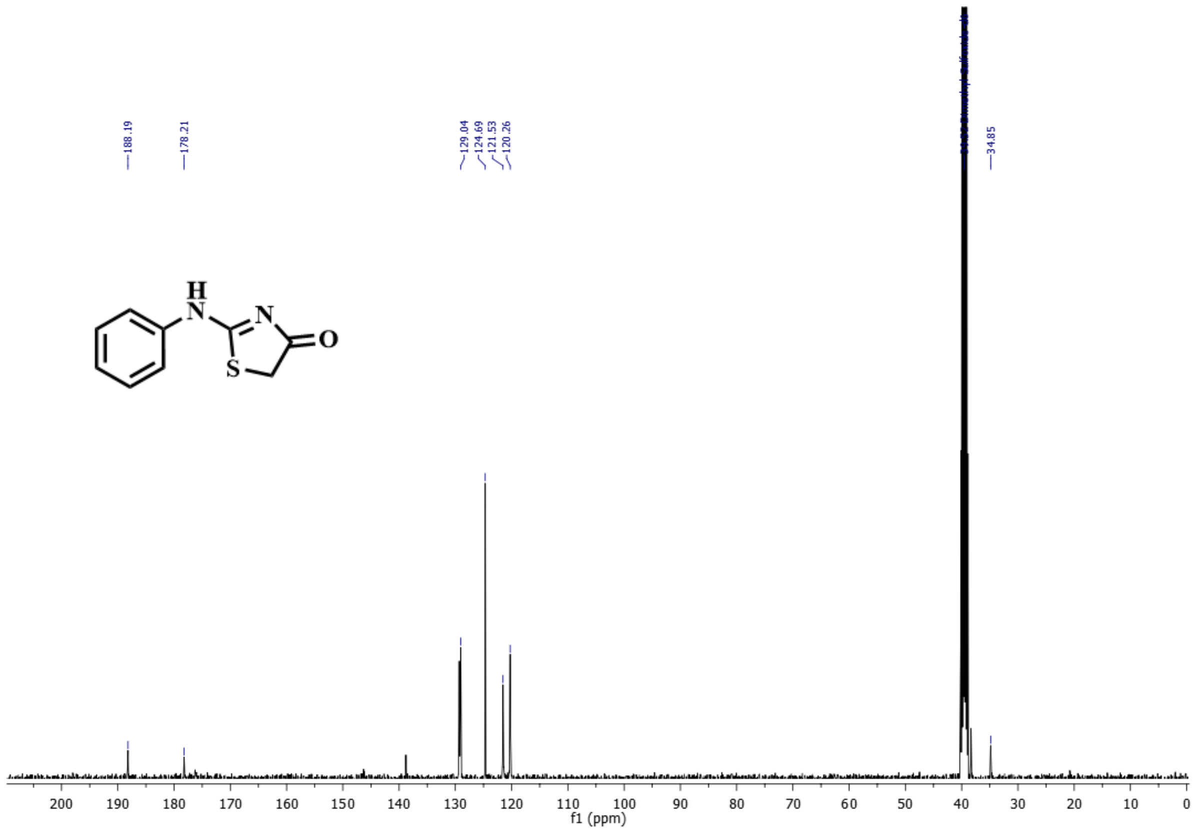

Figure S2: ^{13}C NMR of 2-(phenylamino)thiazol-4(5*H*)-one (3a)

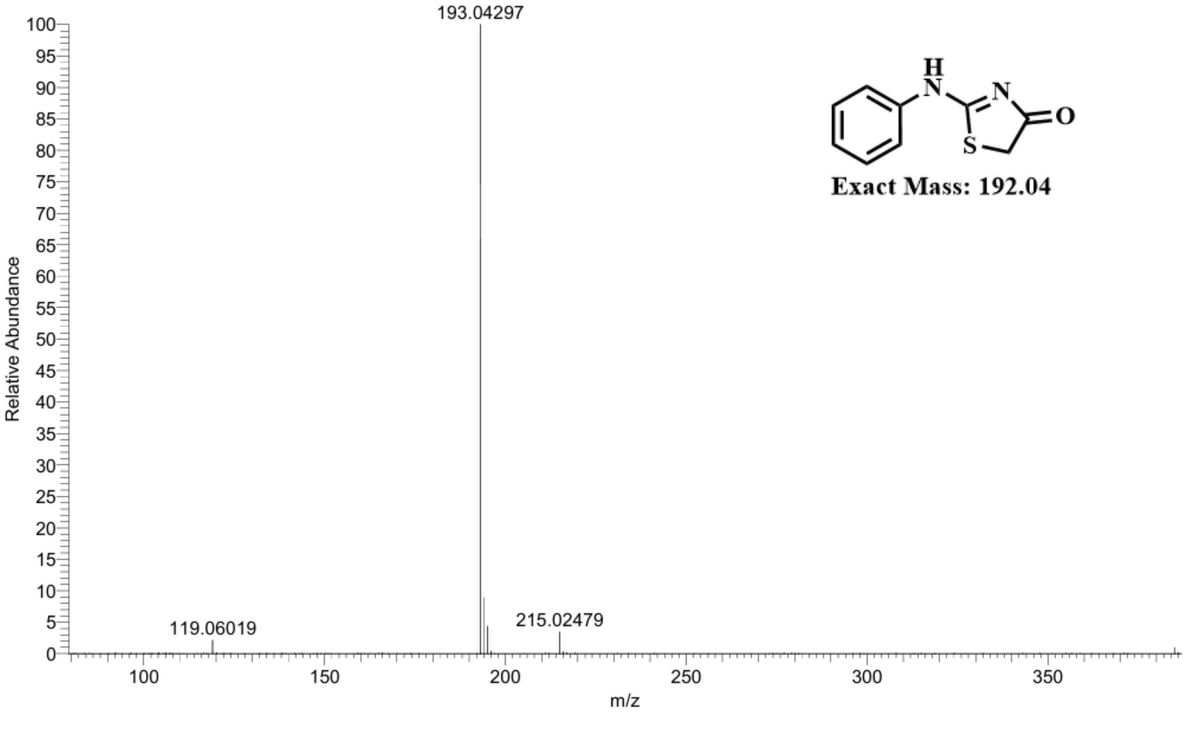

Figure S3: ESI-HRMS of 2-(phenylamino)thiazol-4(5*H*)-one (3a)

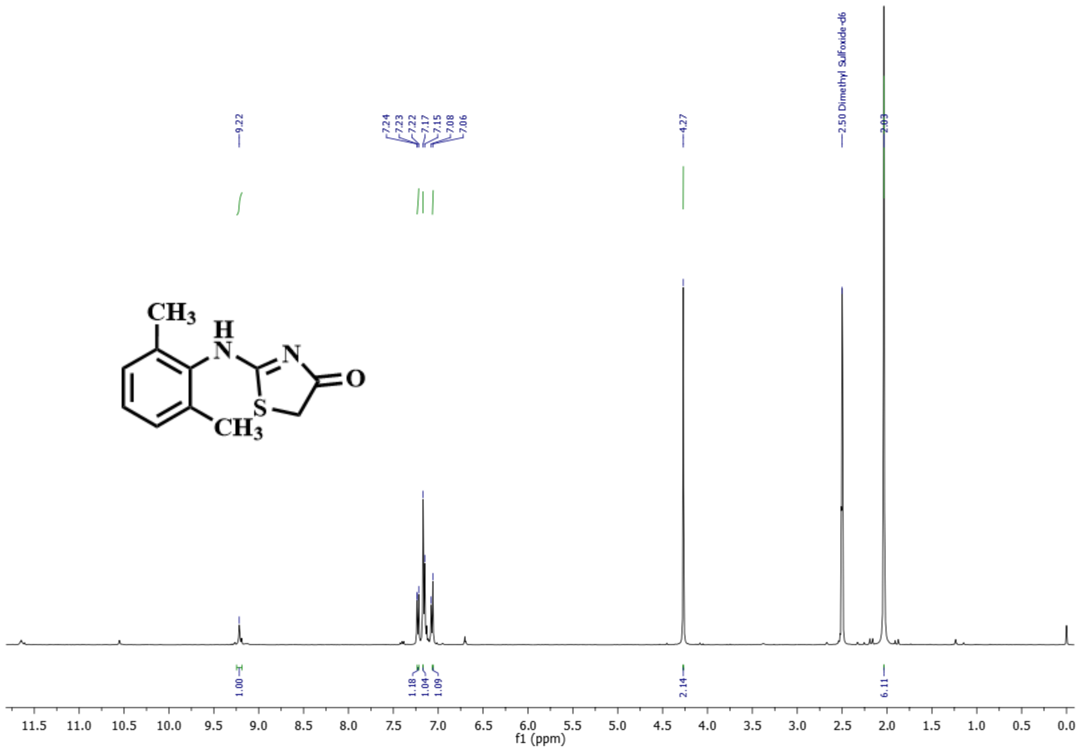

Figure S4: ^{1}H NMR of 2-((2,6-dimethylphenyl)amino)thiazol-4(5*H*)-one (3b)

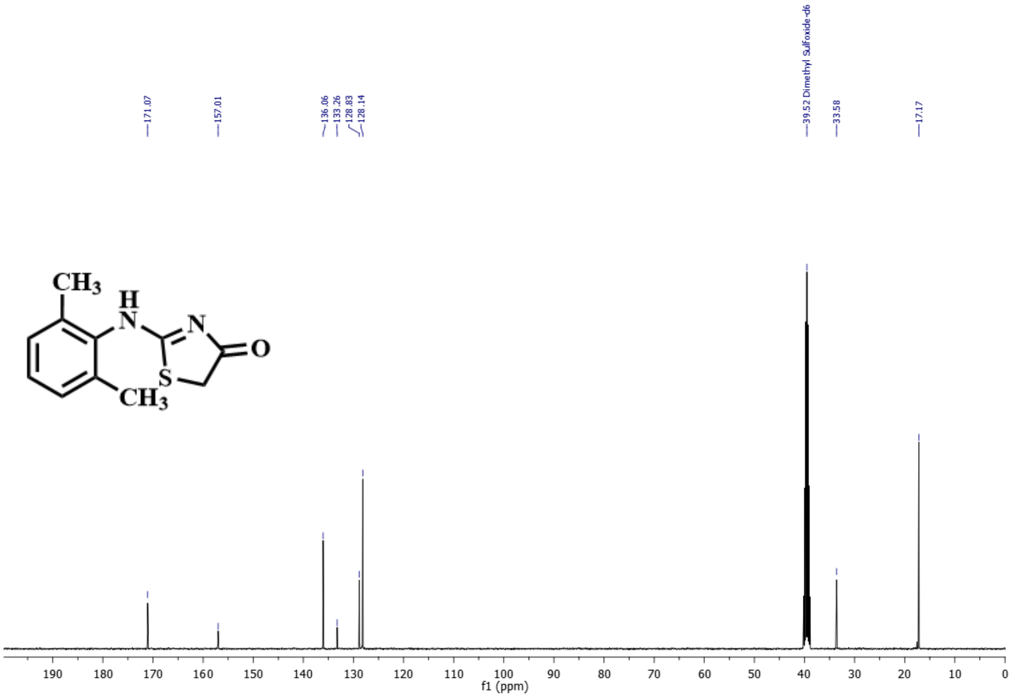

Figure S5: ^{13}C NMR of 2-((2,6-dimethylphenyl)amino)thiazol-4(5*H*)-one (3b)

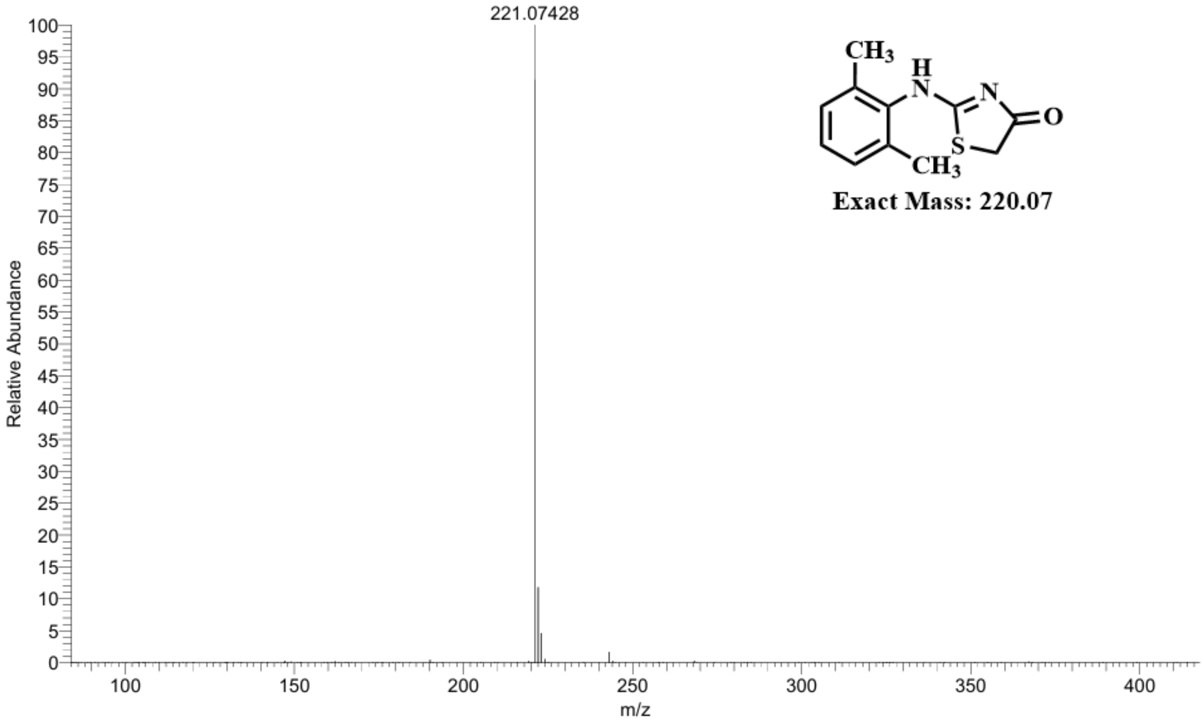

Figure S6: ESI-HRMS of 2-((2,6-dimethylphenyl)amino)thiazol-4(5*H*)-one (3b)

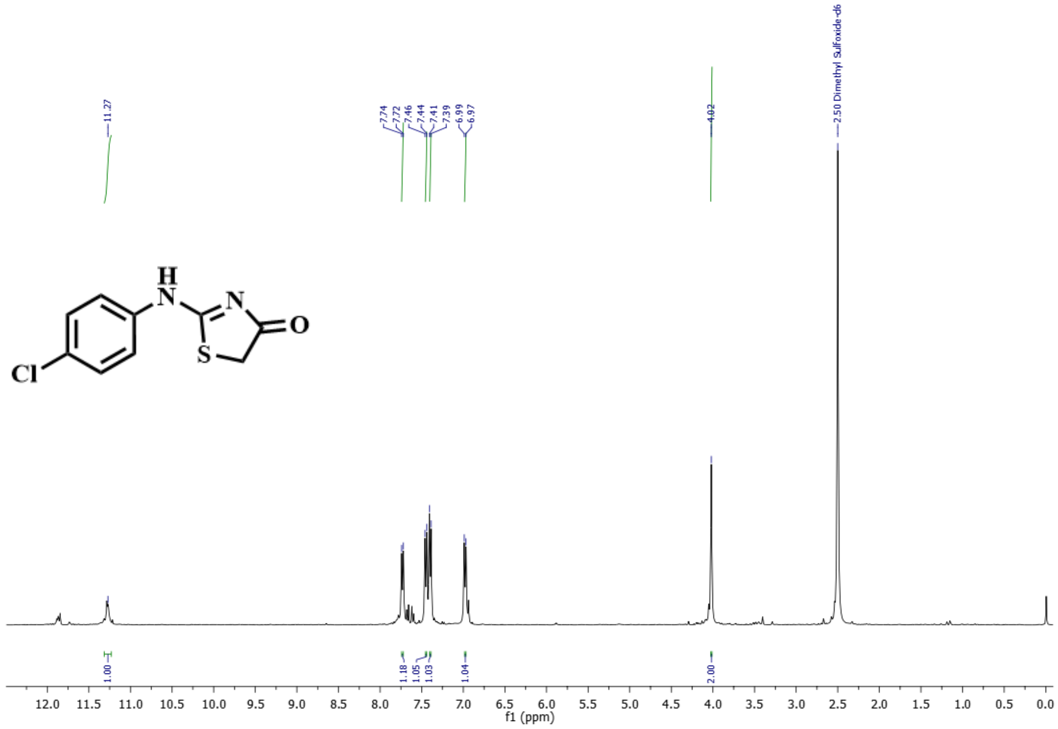

Figure S7: ^{1}H NMR of 2-((4-chlorophenyl)amino)thiazol-4(5*H*)-one (3c)

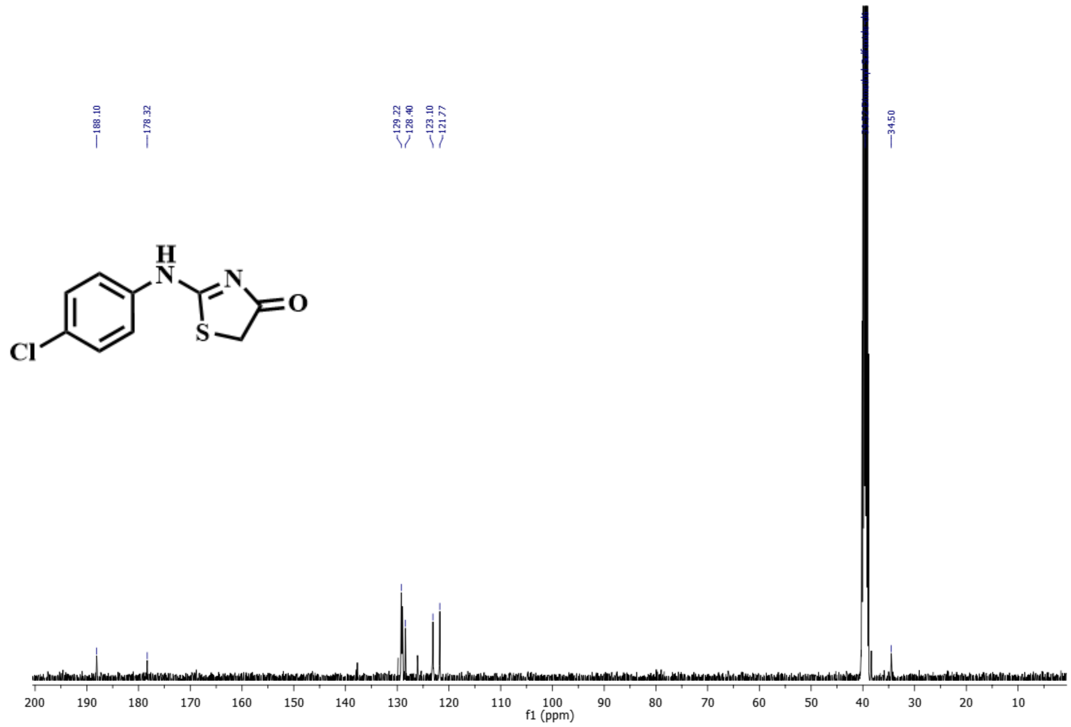

Figure S8: ^{13}C NMR of 2-((4-chlorophenyl)amino)thiazol-4(5*H*)-one (3c)

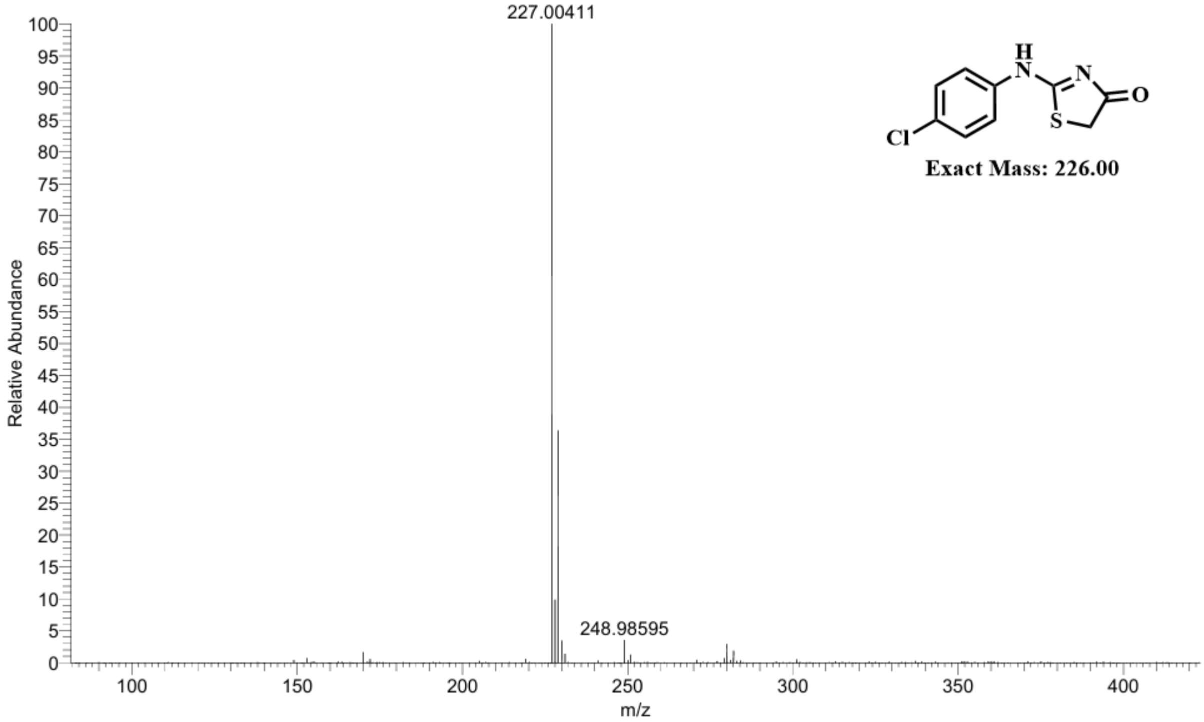

Figure S9: ESI-HRMS of 2-((4-chlorophenyl)amino)thiazol-4(5*H*)-one (3c)

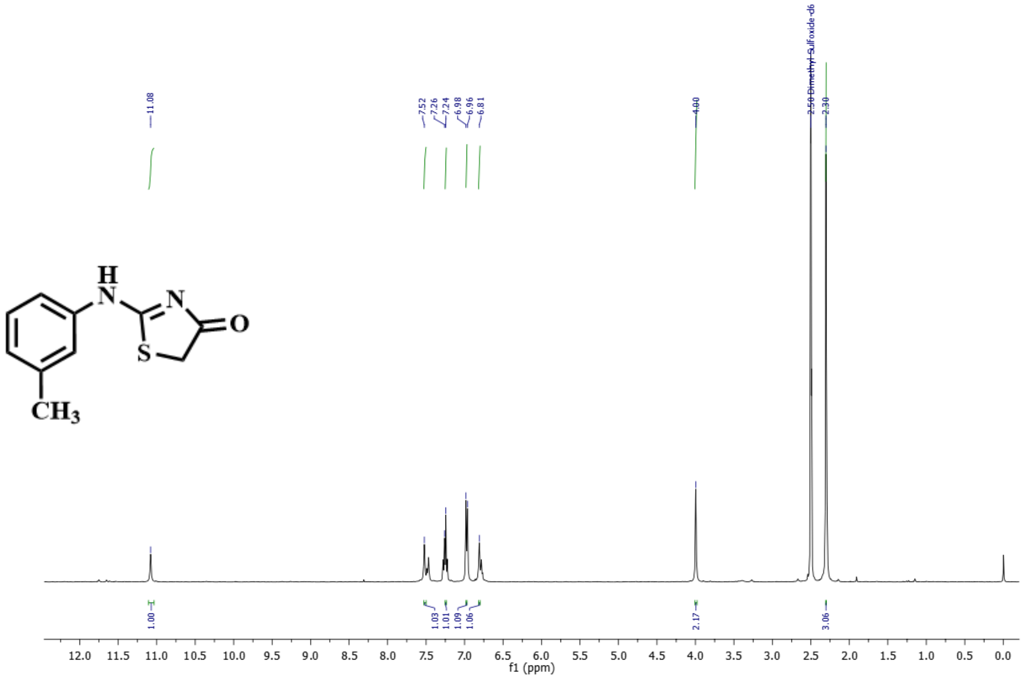

Figure S10: ^{1}H NMR of 2-(*m*-tolylamino)thiazol-4(5*H*)-one (3d)

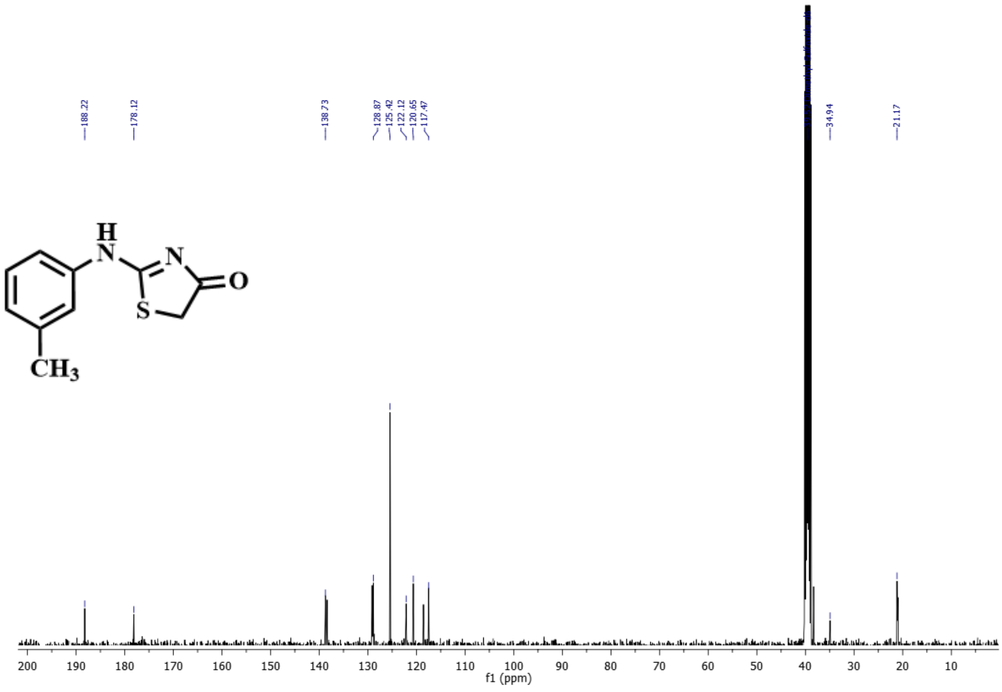

Figure S11: ^{13}C NMR of 2-(*m*-tolylamino)thiazol-4(5*H*)-one (3d)

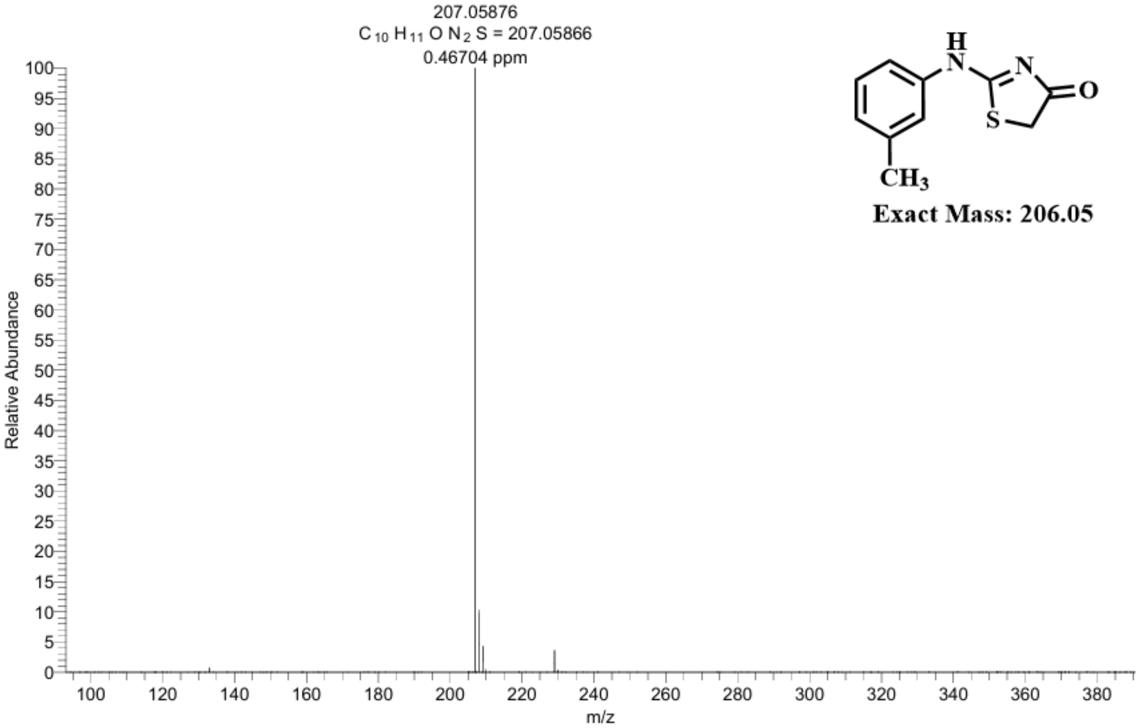

Figure S12: ESI-HRMS of 2-(*m*-tolylamino)thiazol-4(5*H*)-one (3d)

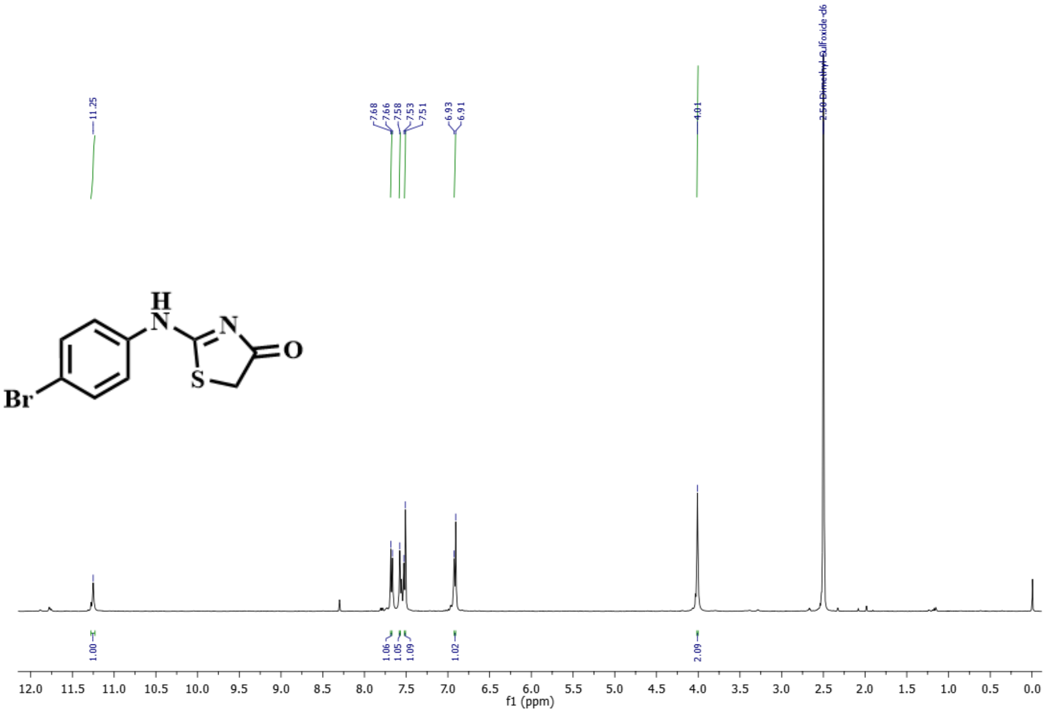

Figure S13: ^{1}H NMR of 2-((4-bromophenyl)amino)thiazol-4(5*H*)-one (3e)

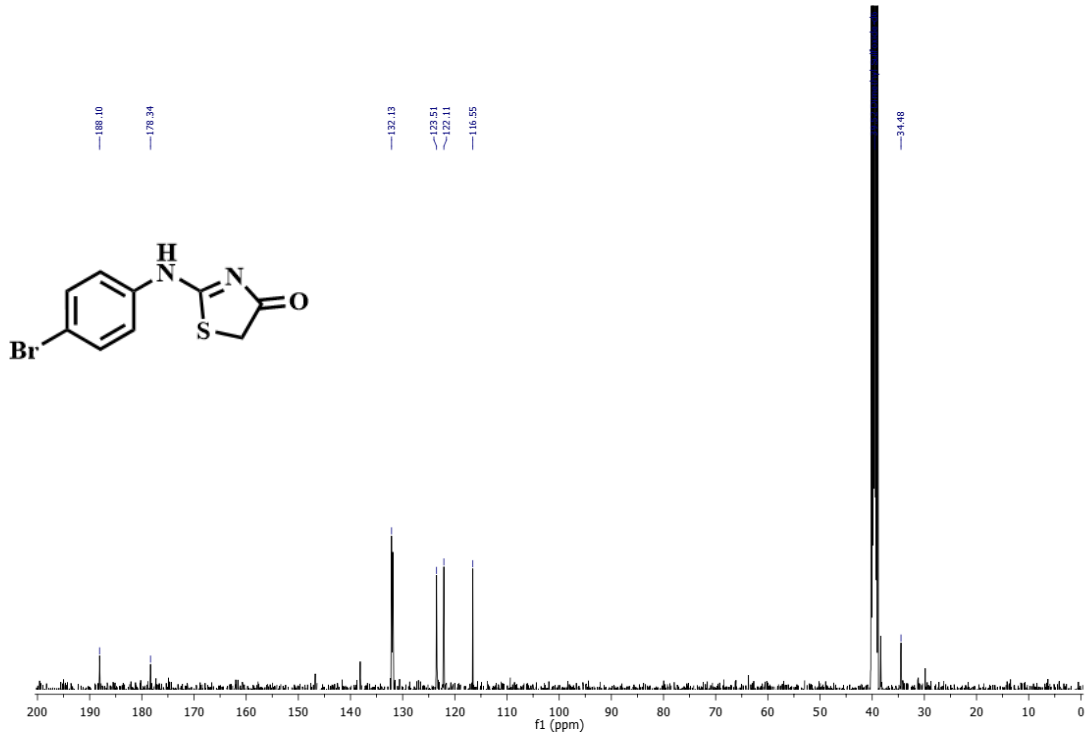

Figure S14: ^{13}C NMR of 2-((4-bromophenyl)amino)thiazol-4(5*H*)-one (3e)

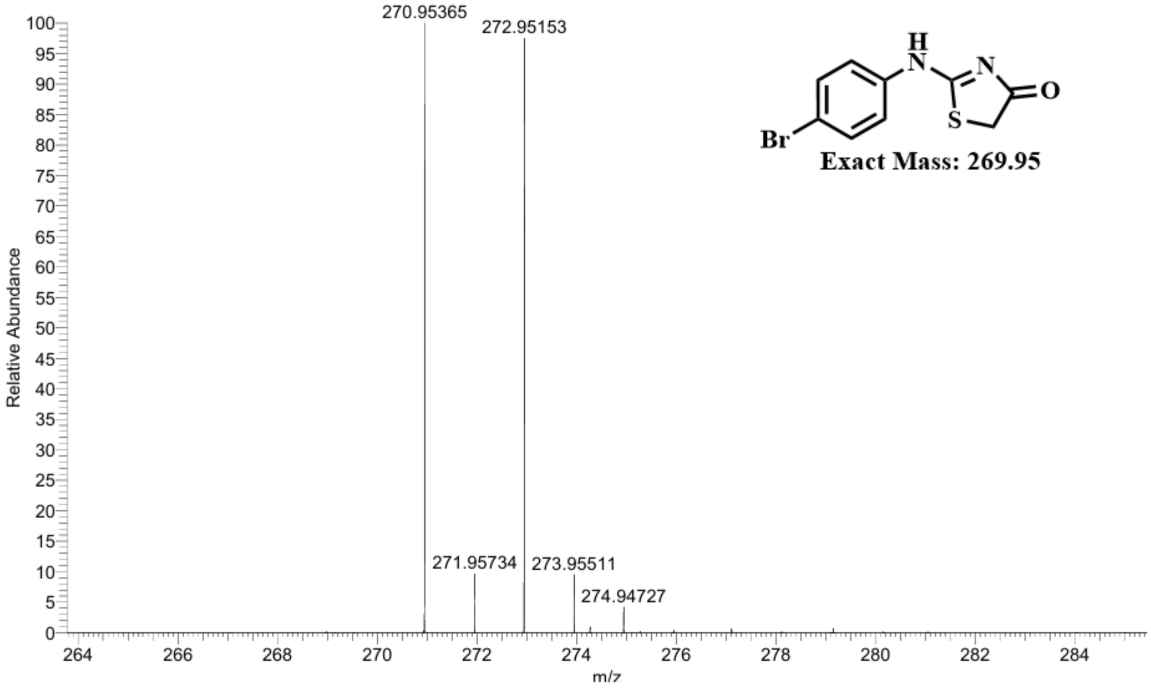

Figure S15: ESI-HRMS of 2-((4-bromophenyl)amino)thiazol-4(5*H*)-one (3e)

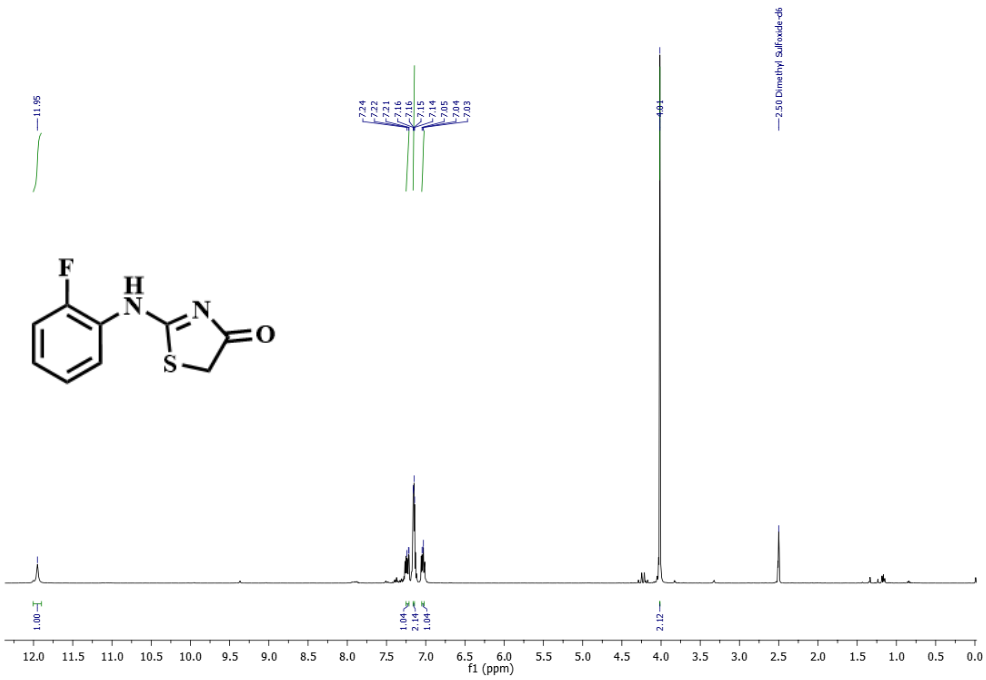

Figure S16: ^{1}H NMR of 2-((2-fluorophenyl)amino)thiazol-4(5*H*)-one (3f)

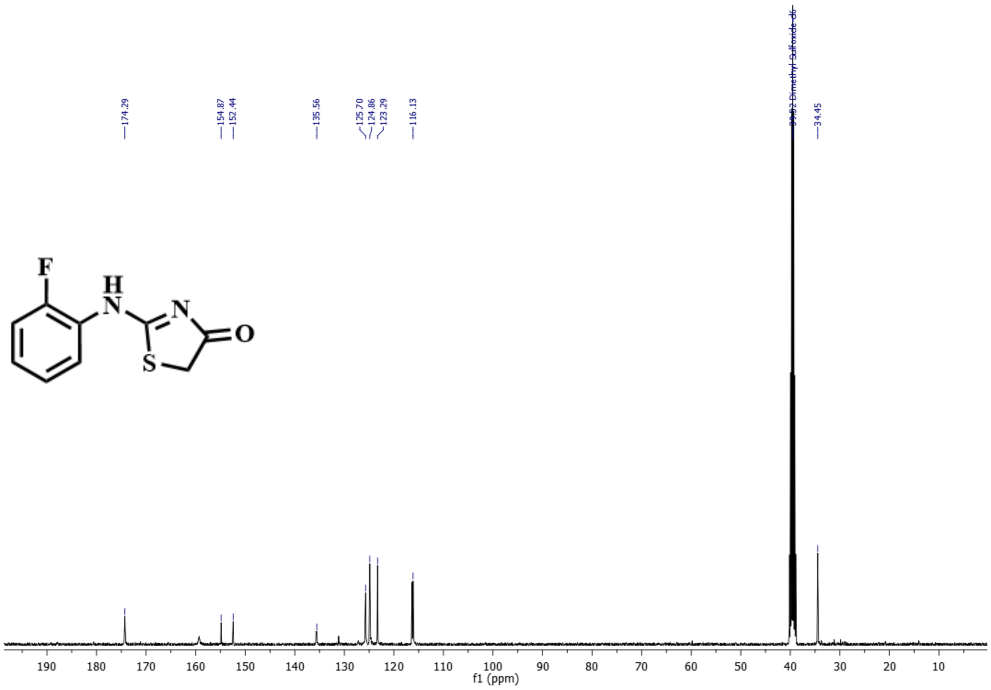

Figure S17: ^{13}C NMR of 2-((2-fluorophenyl)amino)thiazol-4(5*H*)-one (3f)

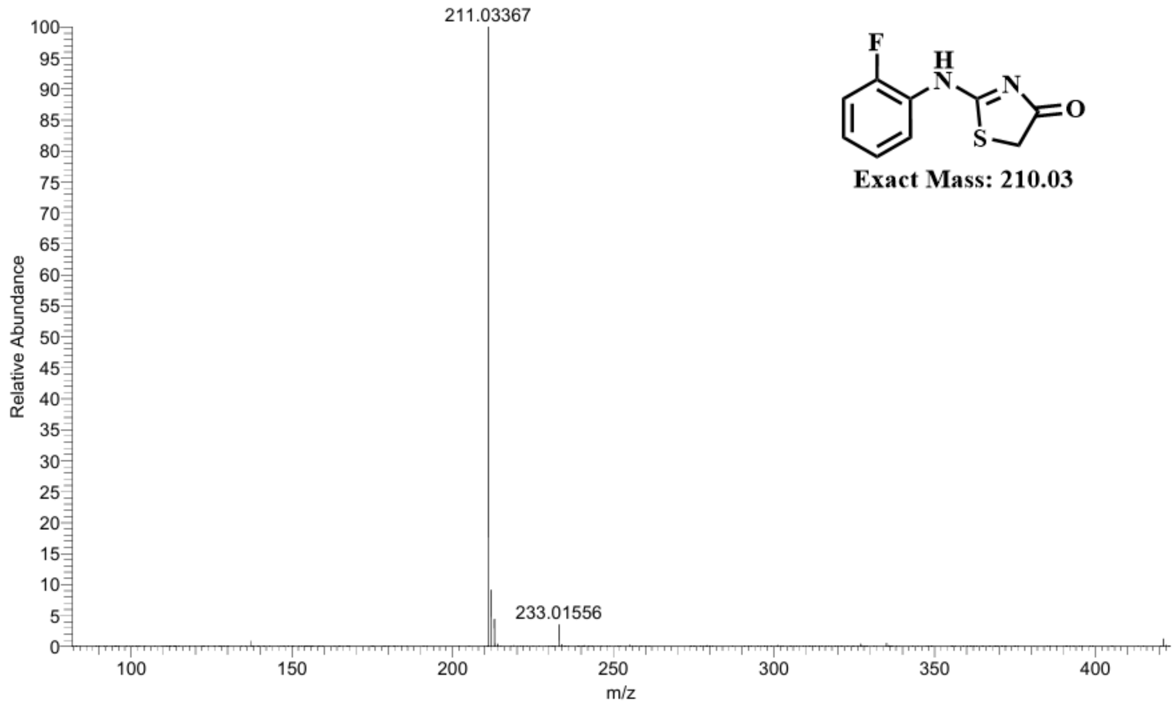

Figure S18: ESI-HRMS of 2-((2-fluorophenyl)amino)thiazol-4(5*H*)-one (3f)

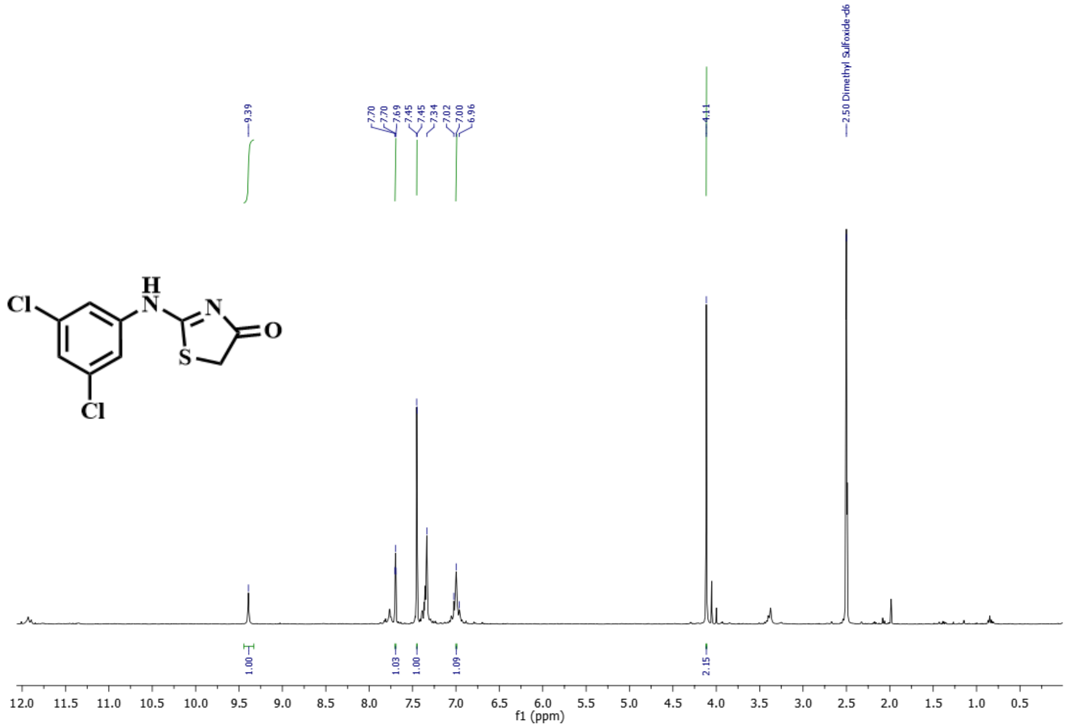

Figure S19: ^{1}H NMR of 2-((3,5-dichlorophenyl)amino)thiazol-4(5*H*)-one (3g)

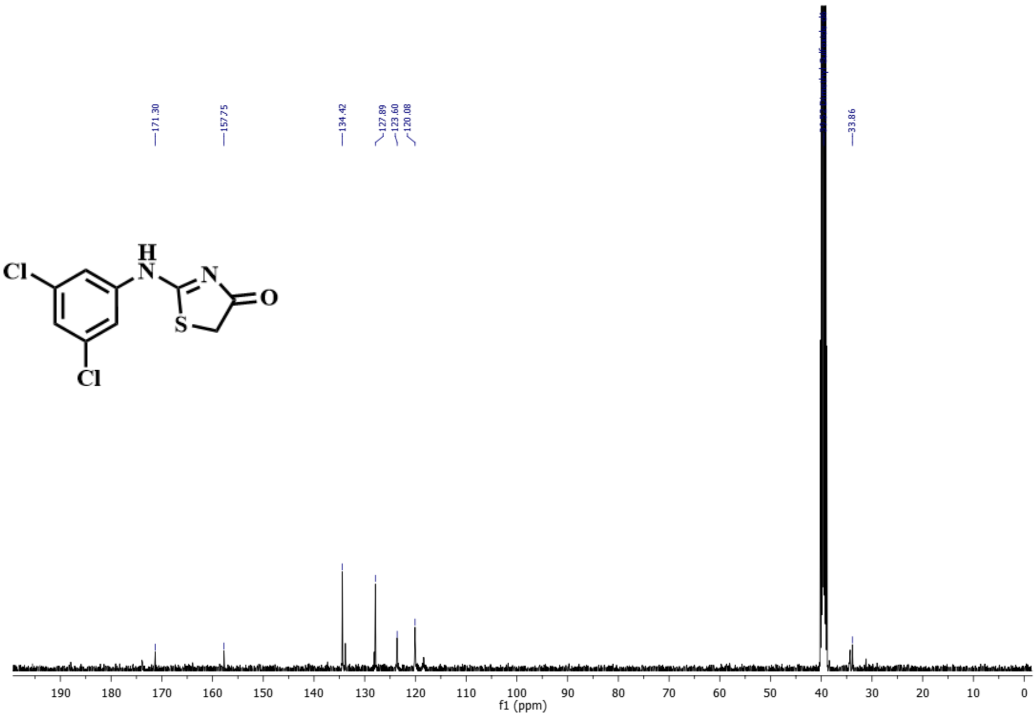

Figure S20: ^{13}C NMR of 2-((3,5-dichlorophenyl)amino)thiazol-4(5*H*)-one (3g)

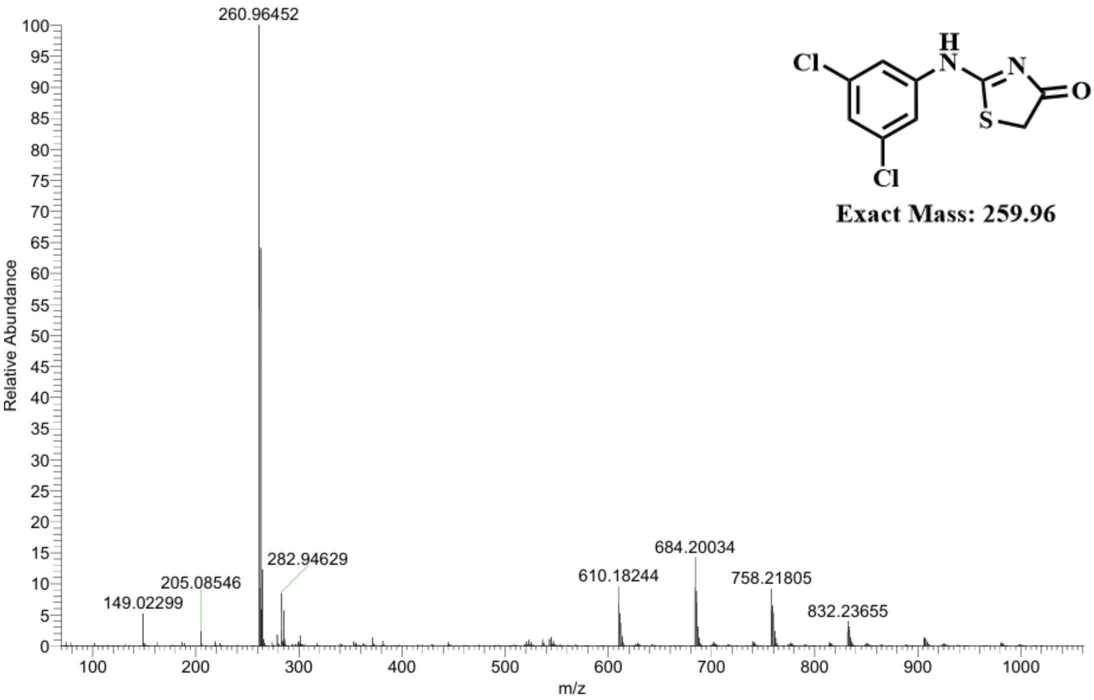

Figure S21: ESI-HRMS of 2-((3,5-dichlorophenyl)amino)thiazol-4(5*H*)-one (3g)

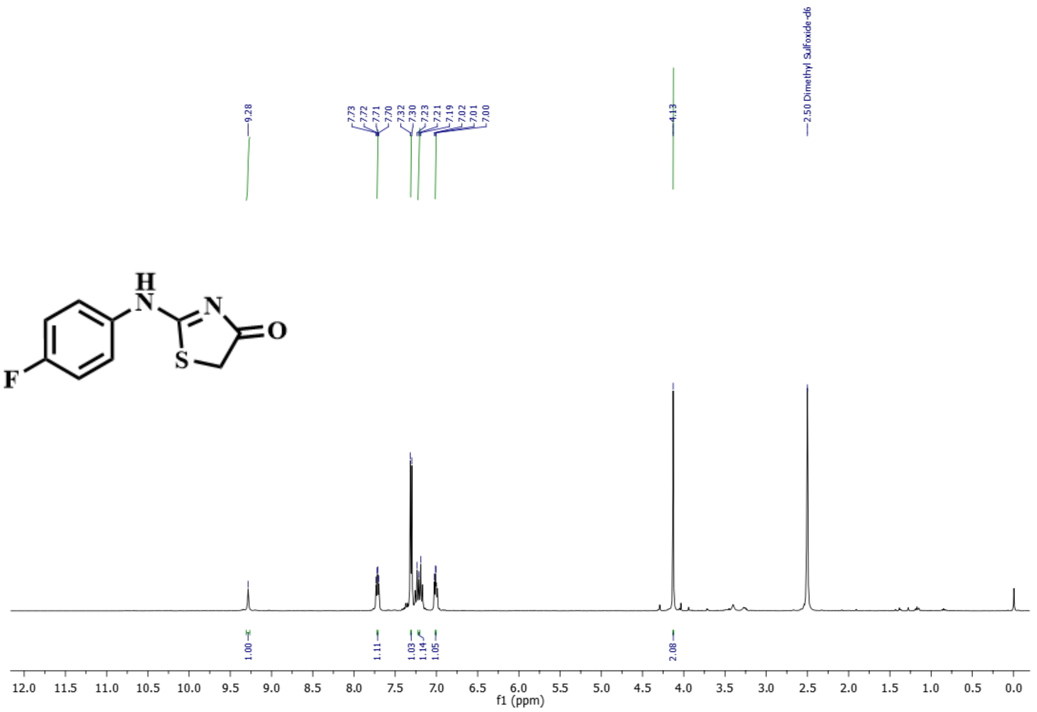

Figure S22: ^{1}H NMR of 2-((4-fluorophenyl)amino)thiazol-4(5*H*)-one (3h)

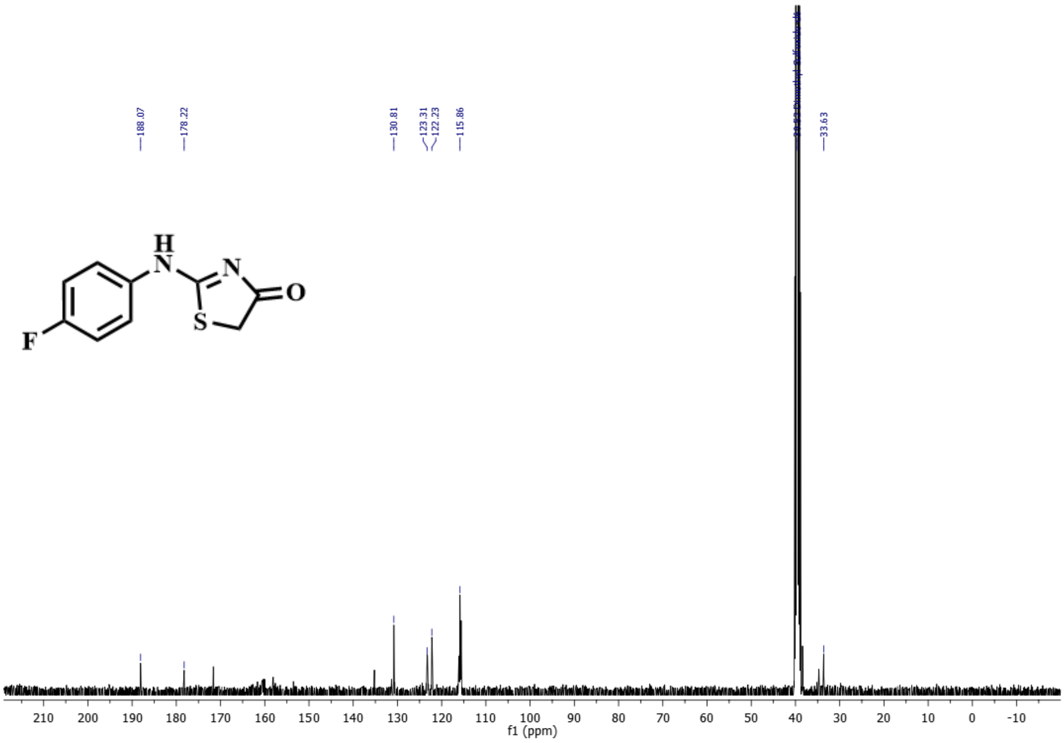

Figure S23: ^{13}C NMR of 2-((4-fluorophenyl)amino)thiazol-4(5*H*)-one (3h)

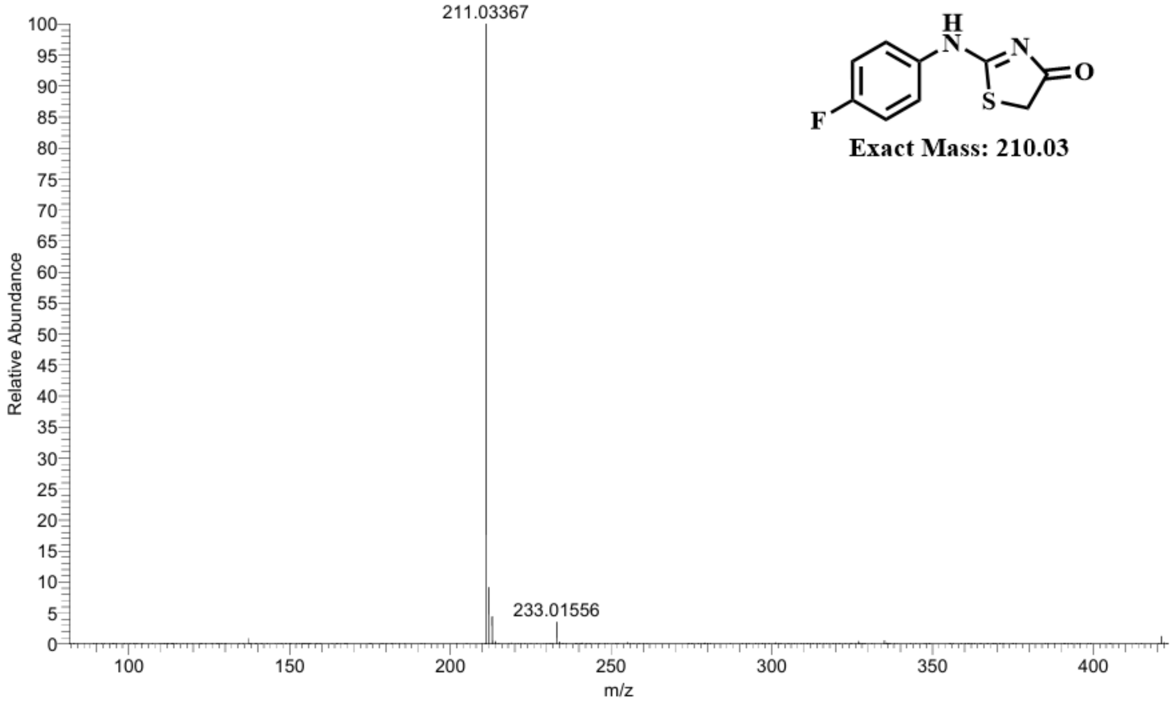

Figure S24: ESI-HRMS of 2-((4-fluorophenyl)amino)thiazol-4(5*H*)-one (3h)

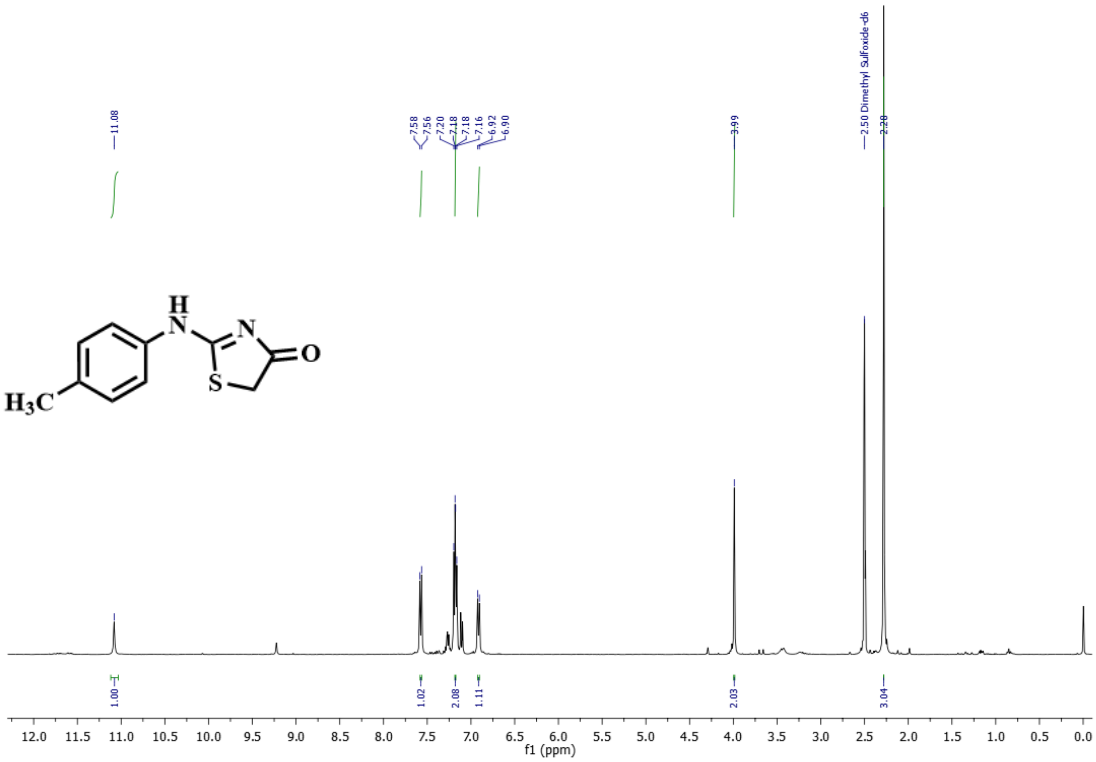

Figure S25: ^{1}H NMR of 2-(*p*-tolylamino)thiazol-4(5*H*)-one (3i)

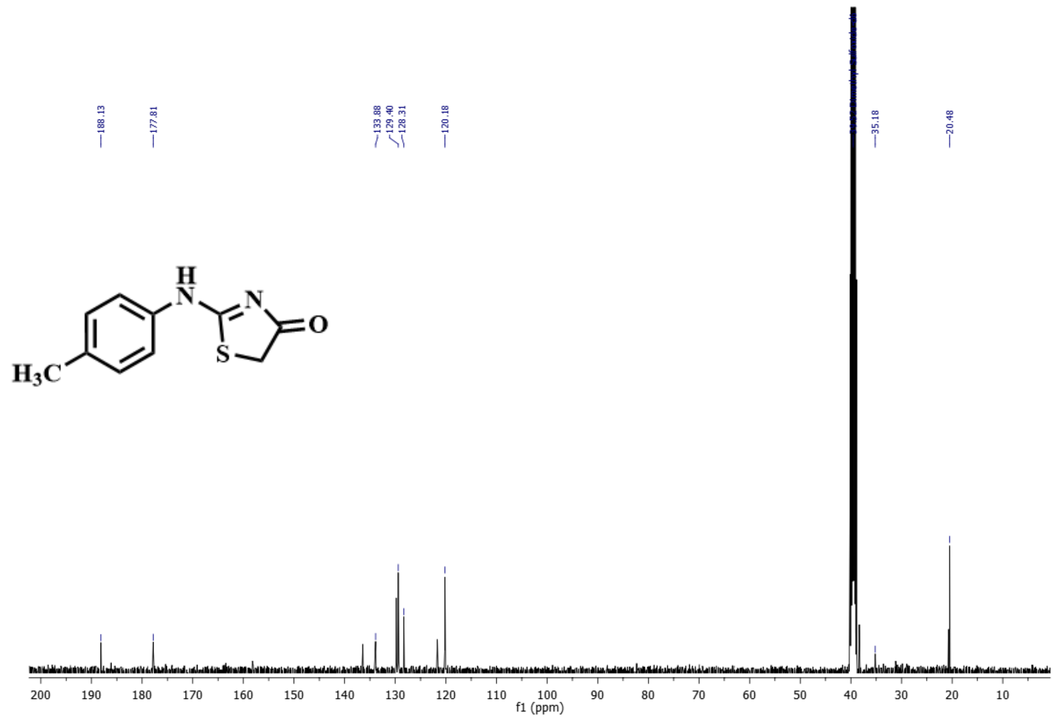

Figure S26: ^{13}C NMR of 2-(*p*-tolylamino)thiazol-4(5*H*)-one (3i)

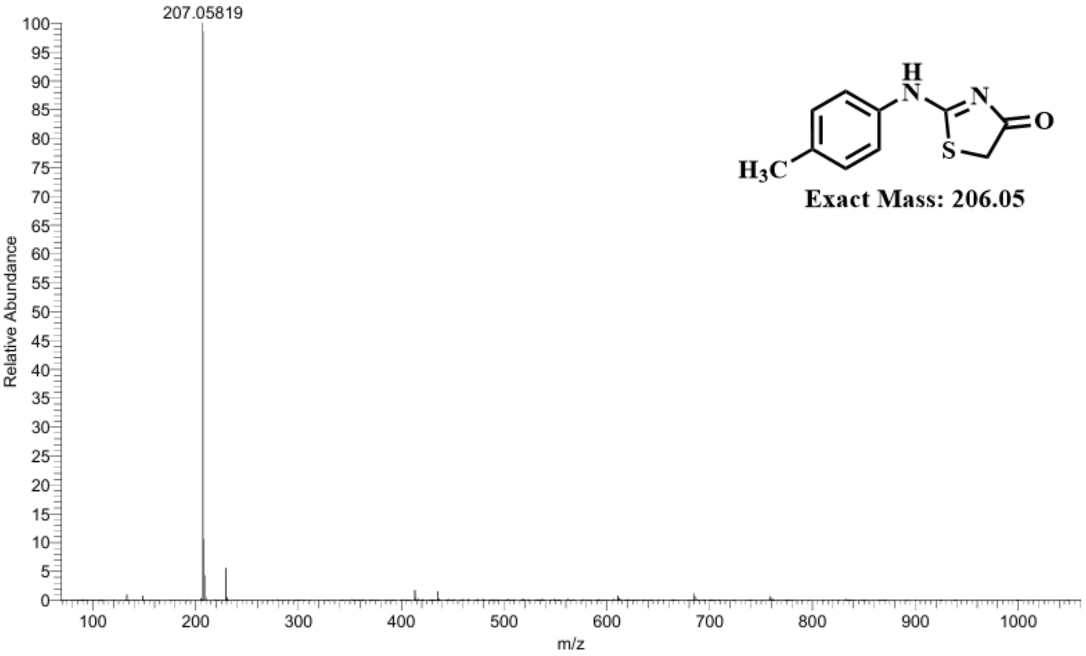

Figure S27: ESI-HRMS of 2-(*p*-tolylamino)thiazol-4(5*H*)-one (3i)

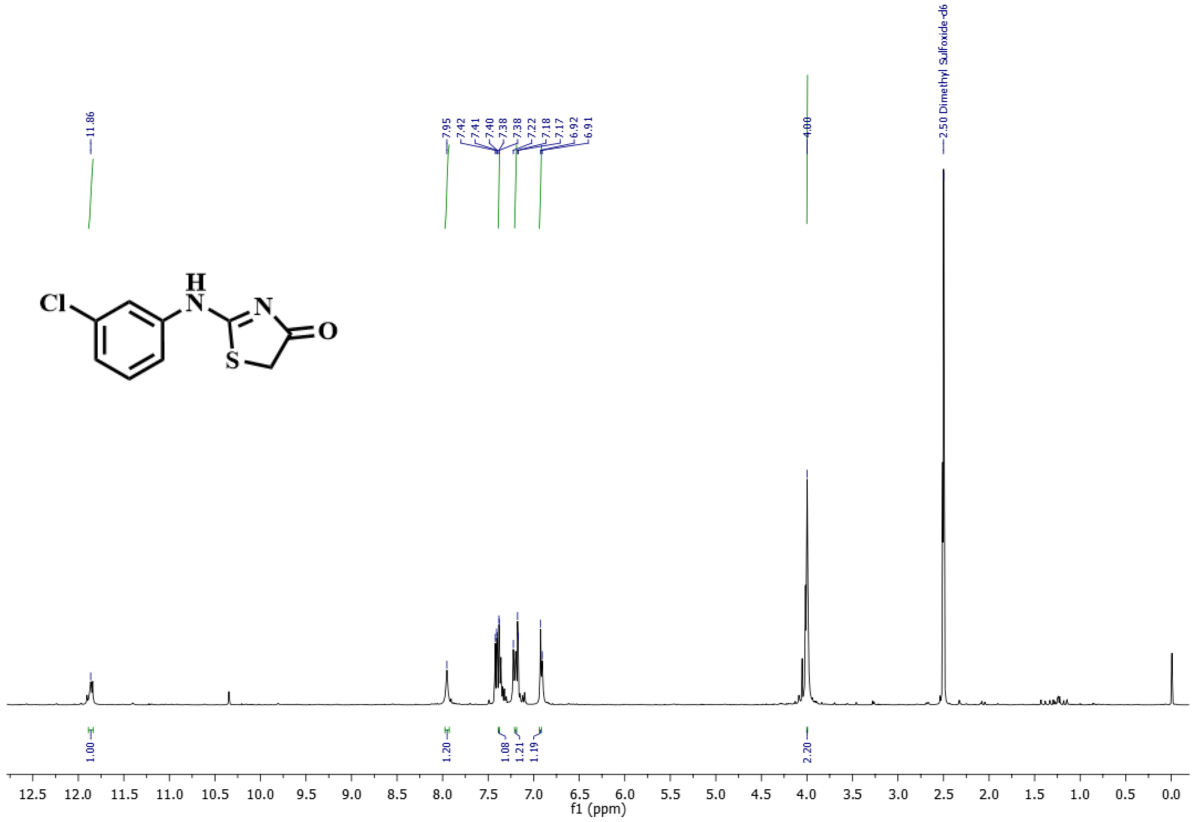

Figure S28: ^{1}H NMR of 2-((3-chlorophenyl)amino)thiazol-4(5*H*)-one (3j)

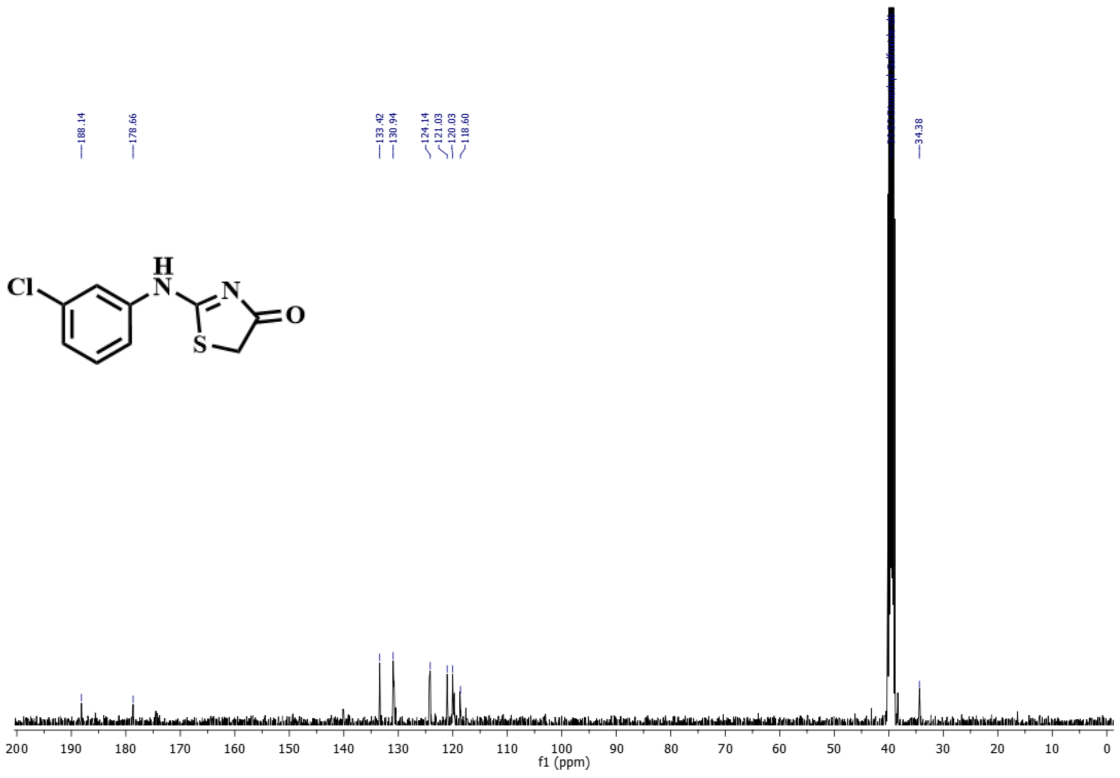

Figure S29: ^{13}C NMR of 2-((3-chlorophenyl)amino)thiazol-4(5*H*)-one (3j)

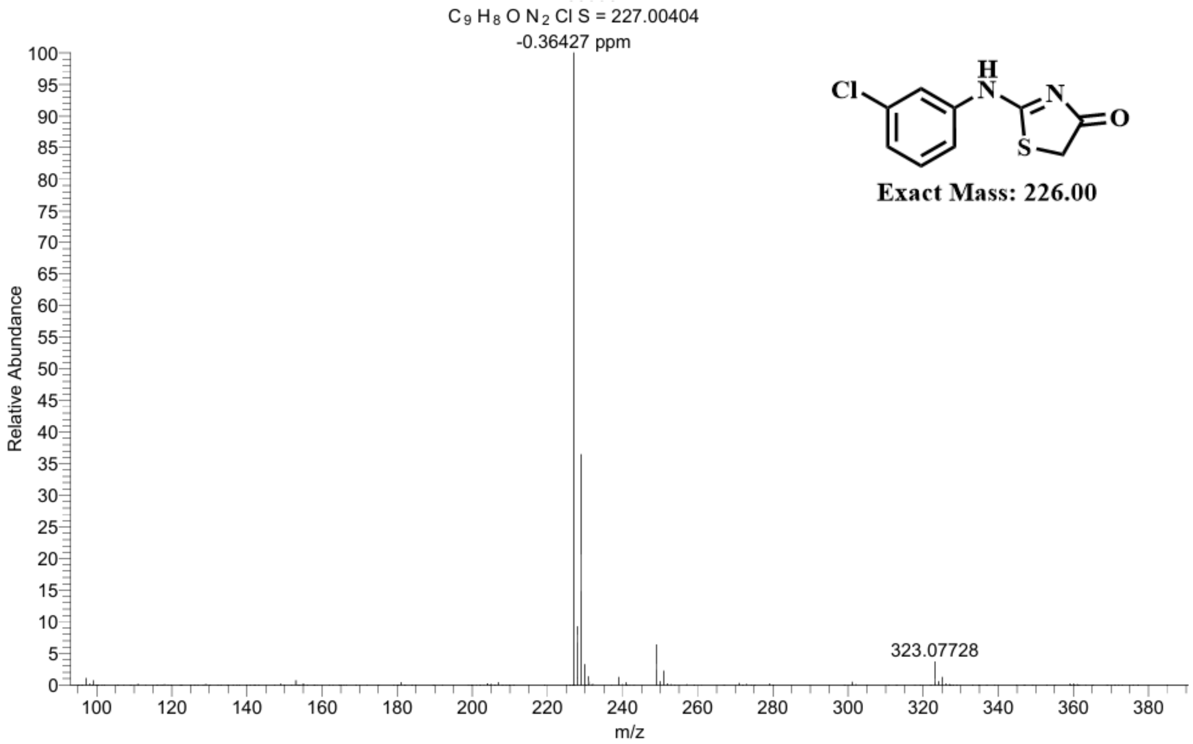

Figure S30: ESI-HRMS of 2-((3-chlorophenyl)amino)thiazol-4(5*H*)-one (3j)

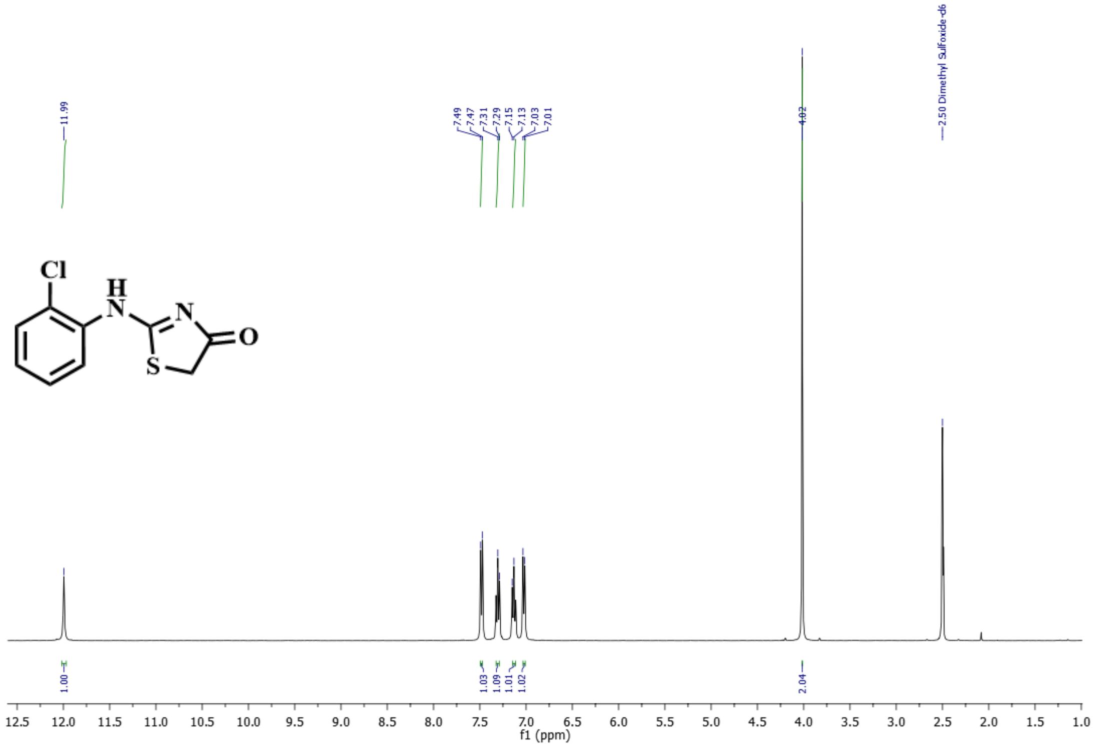

Figure S31: ^{1}H NMR of 2-((2-chlorophenyl)amino)thiazol-4(5*H*)-one (3k)

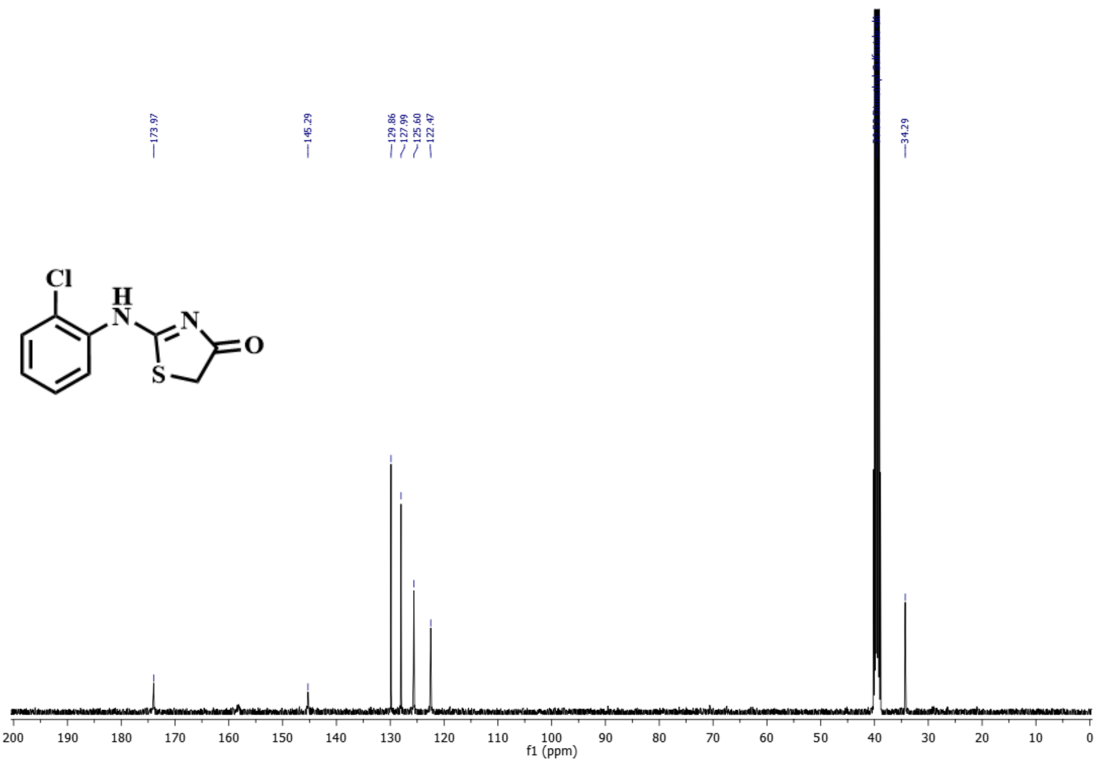

Figure S32: ^{13}C NMR of 2-((2-chlorophenyl)amino)thiazol-4(5*H*)-one (3k)

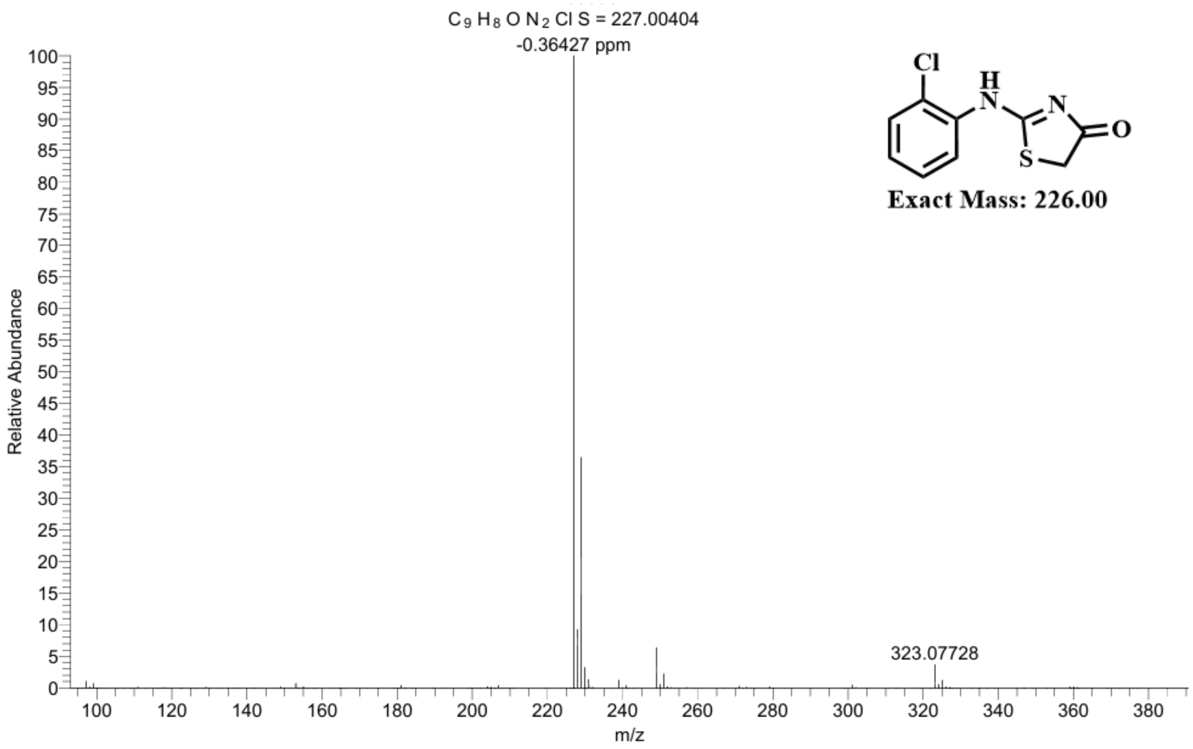

Figure S33: ESI-HRMS of 2-((2-chlorophenyl)amino)thiazol-4(5*H*)-one (3k)

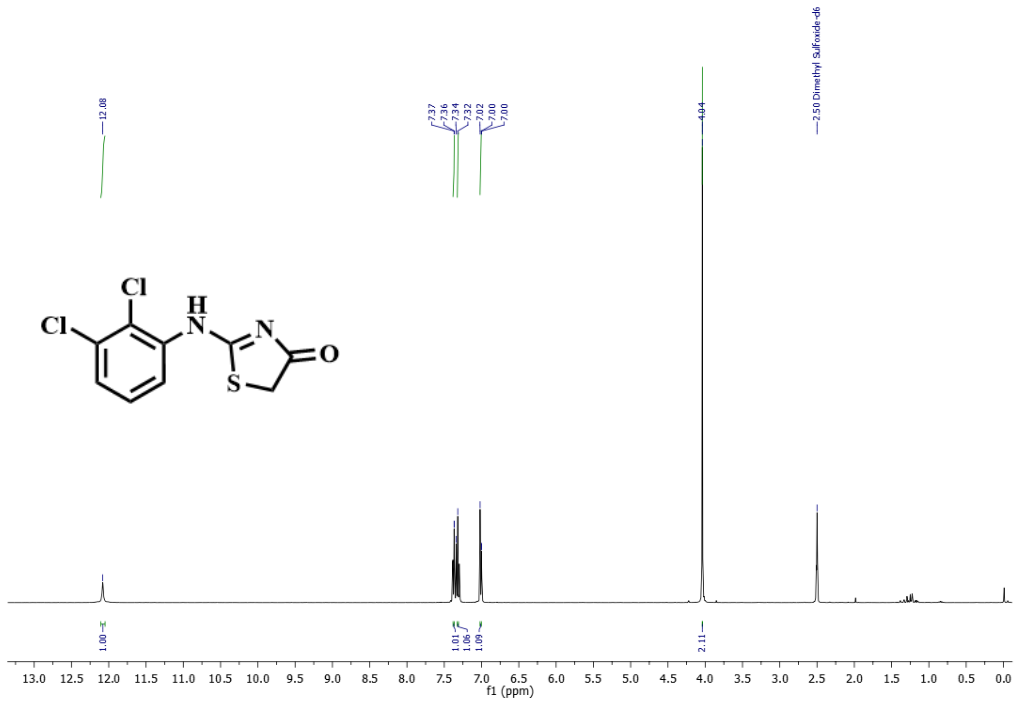

Figure S34: ^{1}H NMR of 2-((2,3-dichlorophenyl)amino)thiazol-4(5*H*)-one (3l)

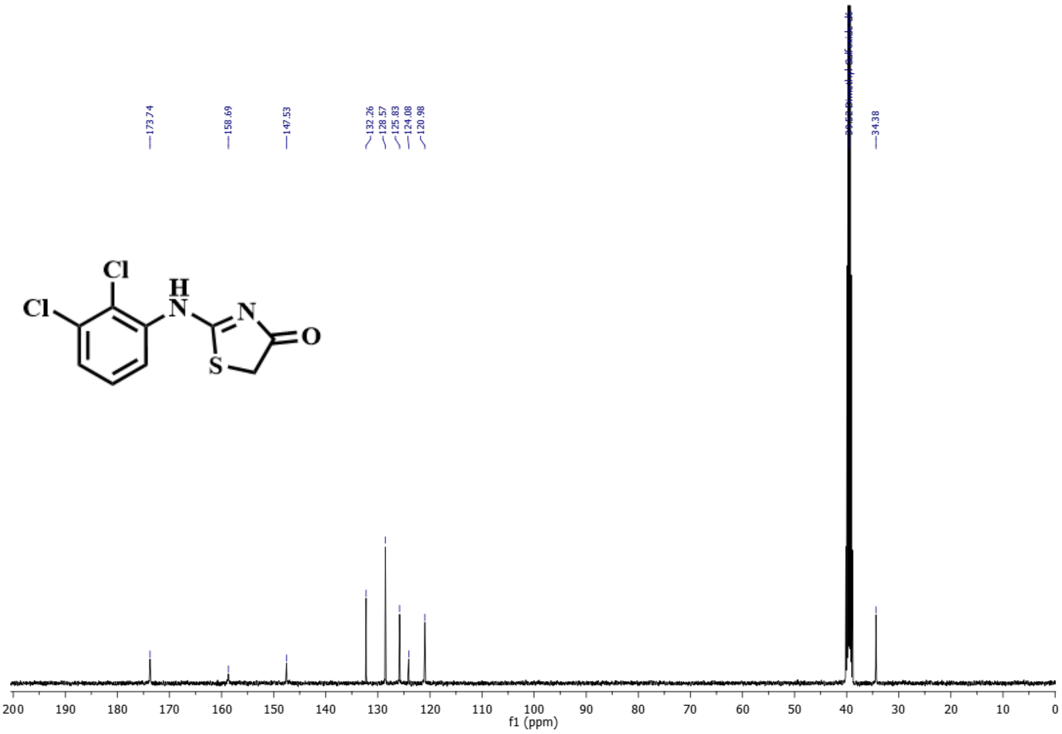

Figure S35: ^{13}C NMR of 2-((2,3-dichlorophenyl)amino)thiazol-4(5*H*)-one (3l)

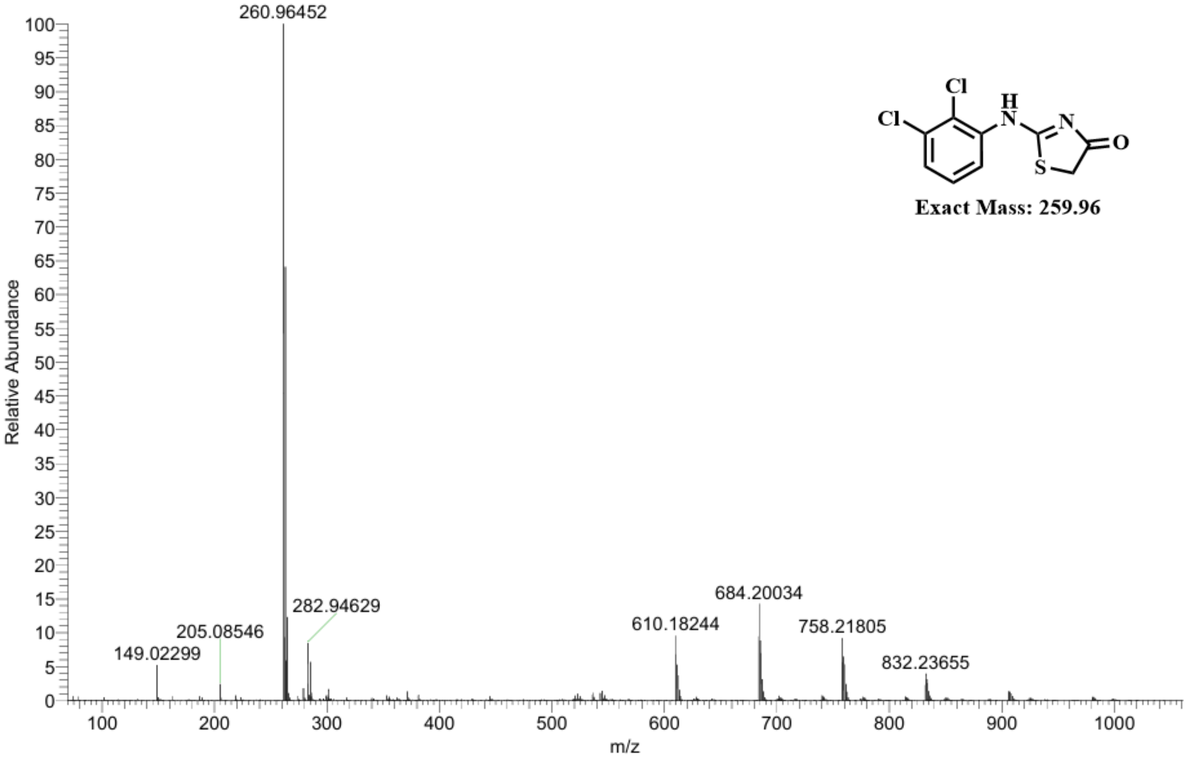

Figure S36: ESI-HRMS of 2-((2,3-dichlorophenyl)amino)thiazol-4(5*H*)-one (3l)

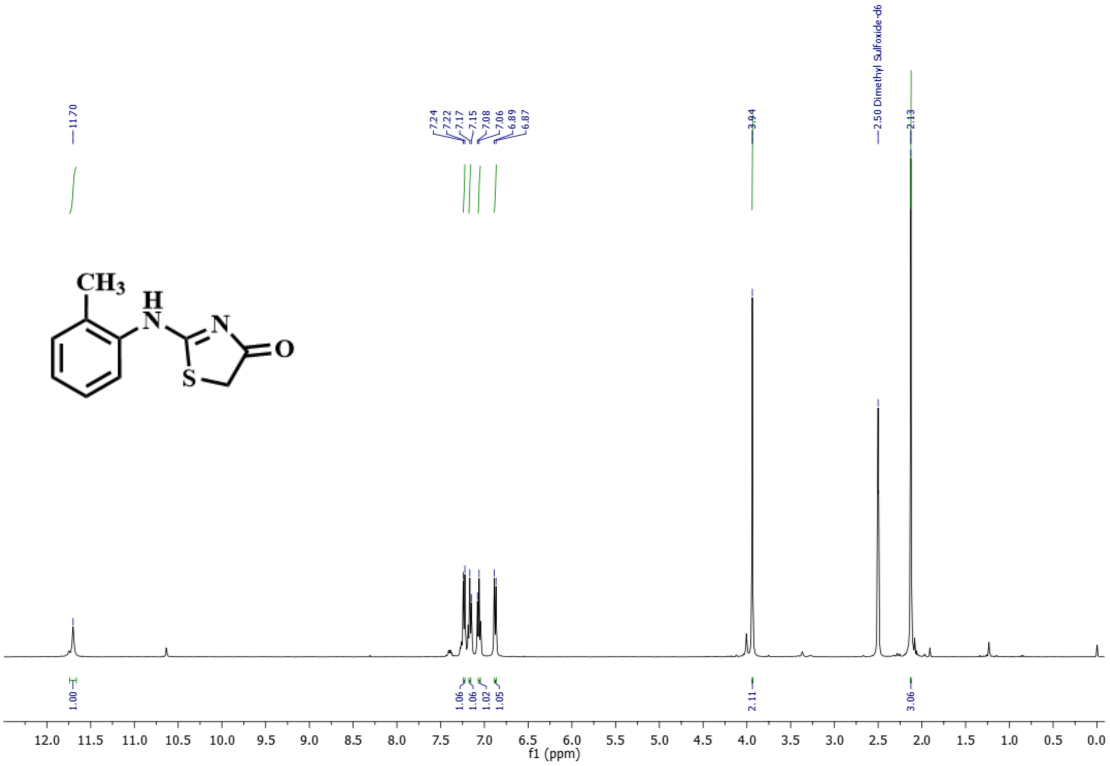

Figure S37: ^{1}H NMR of 2-(*o*-tolylamino)thiazol-4(5*H*)-one (3m)

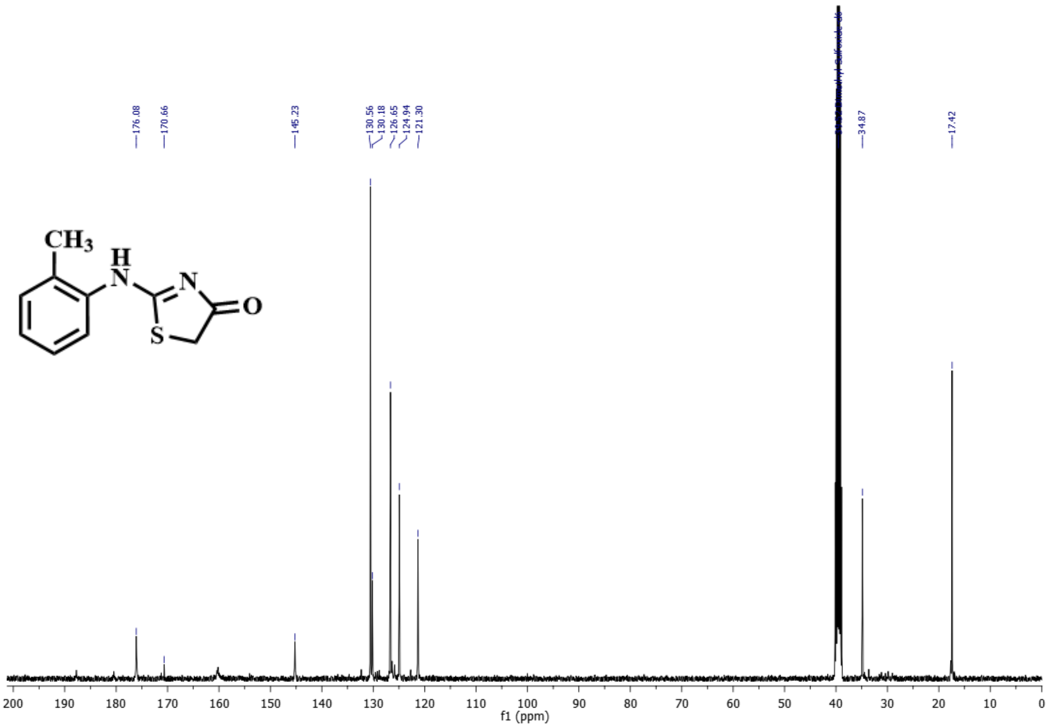

Figure S38: ^{13}C NMR of 2-(*o*-tolylamino)thiazol-4(5*H*)-one (3m)

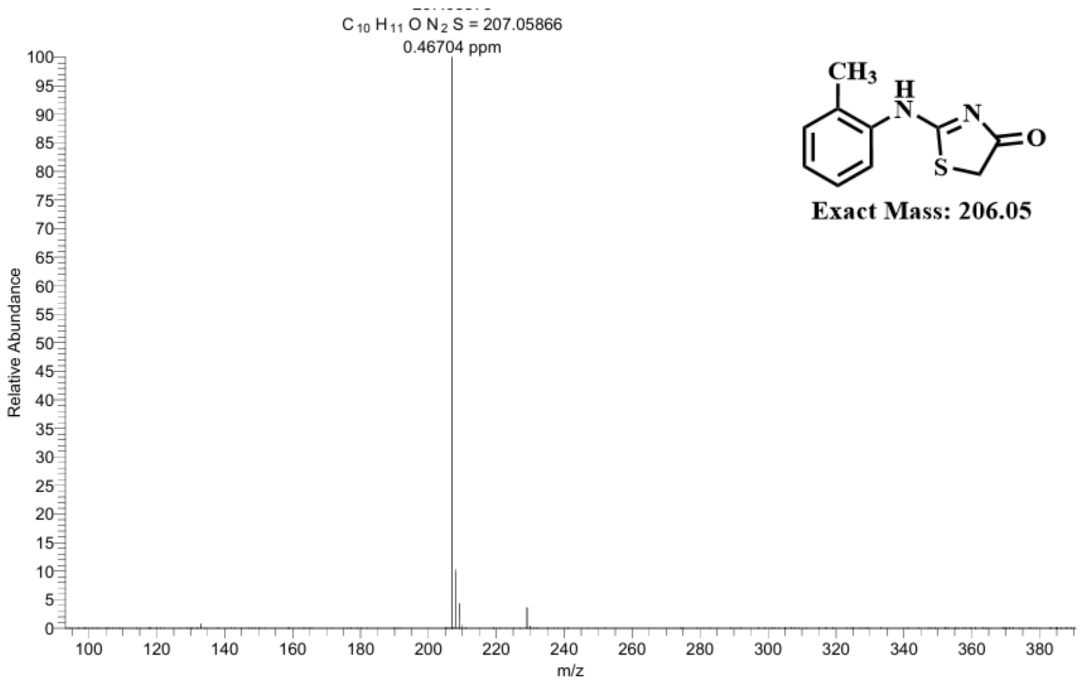

Figure S39: ESI-HRMS of 2-(*o*-tolylamino)thiazol-4(5*H*)-one (3m)

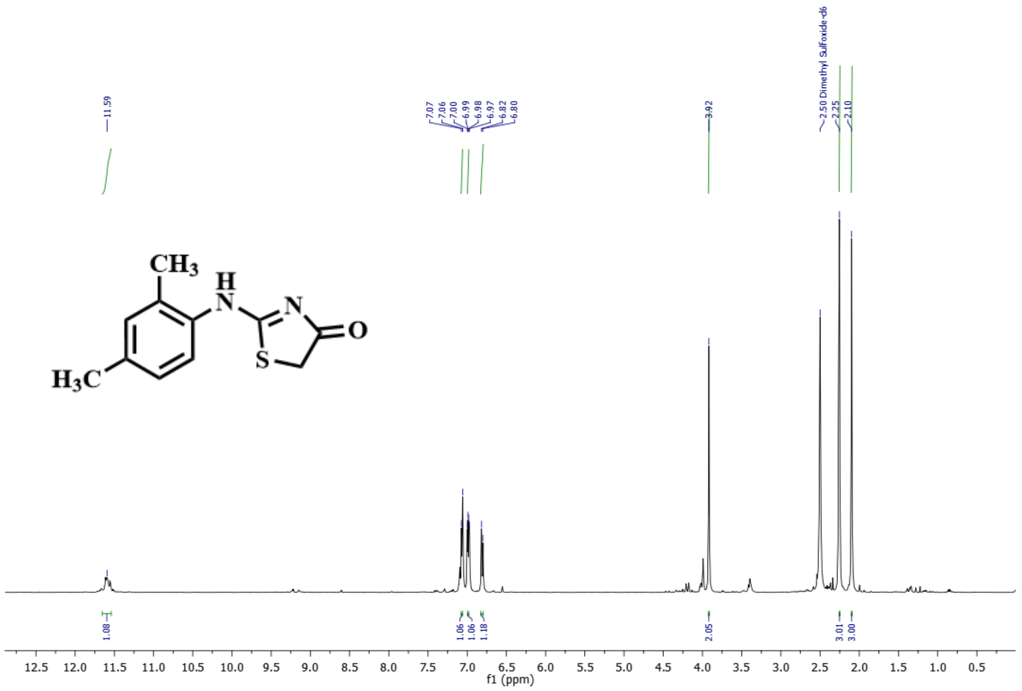

Figure S40: ^{1}H NMR of 2-((2,4-dimethylphenyl)amino)thiazol-4(5*H*)-one (3n)

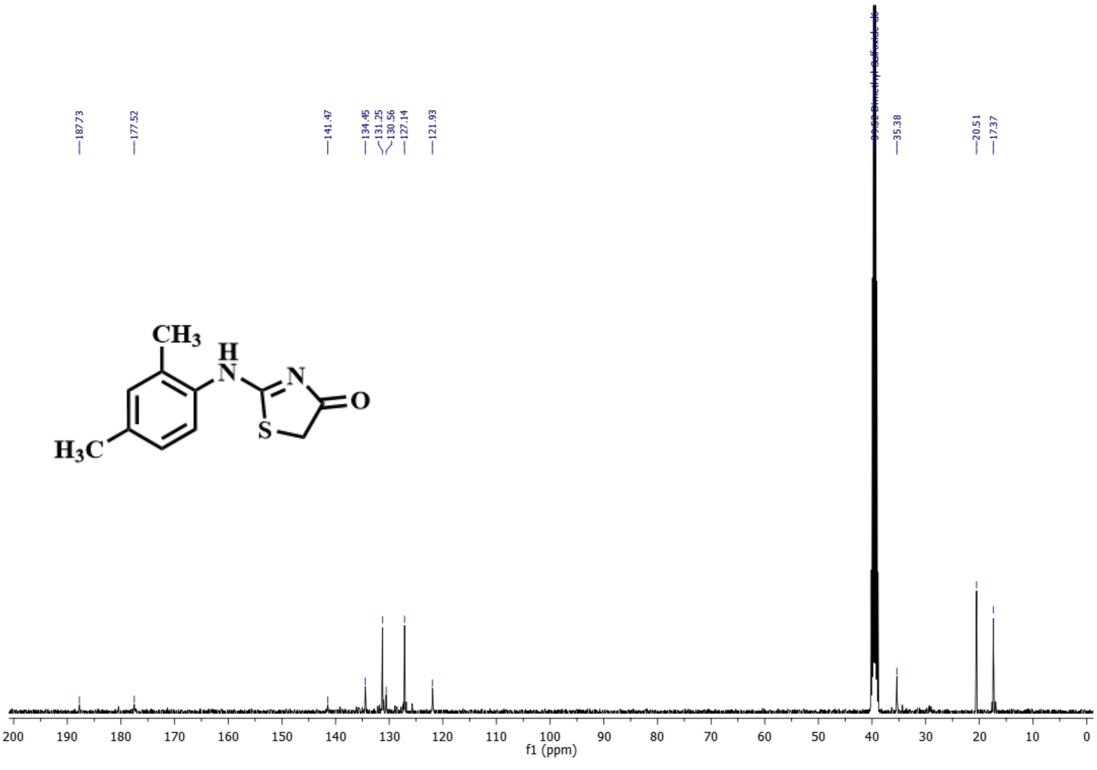

Figure S41: ^{13}C NMR of 2-((2,4-dimethylphenyl)amino)thiazol-4(5*H*)-one (3n)

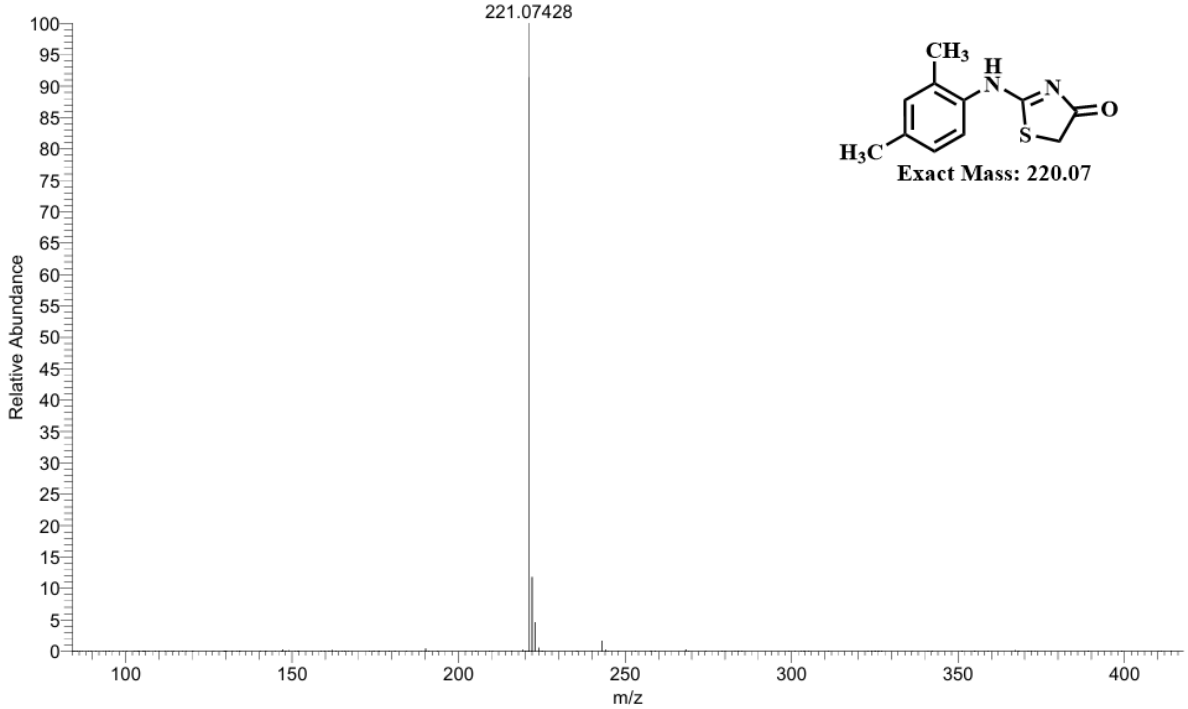

Figure S42: ESI-HRMS of 2-((2,4-dimethylphenyl)amino)thiazol-4(5*H*)-one (3n)

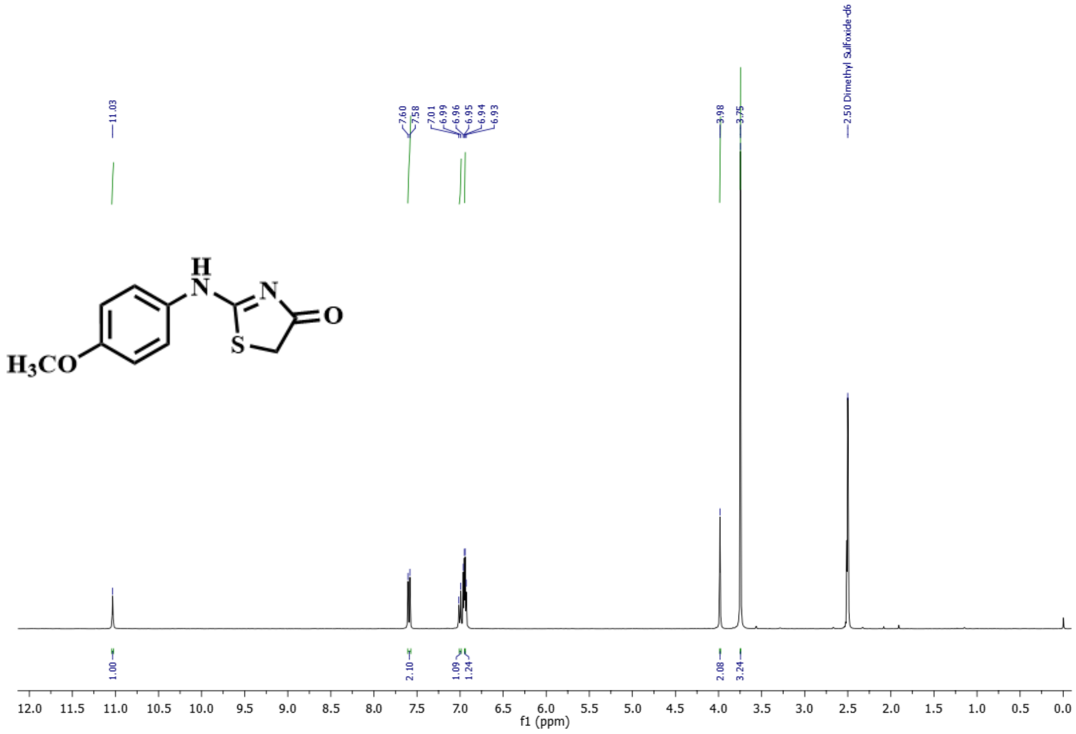

Figure S43: ^{1}H NMR of 2-((4-methoxyphenyl)amino)thiazol-4(5*H*)-one (3o)

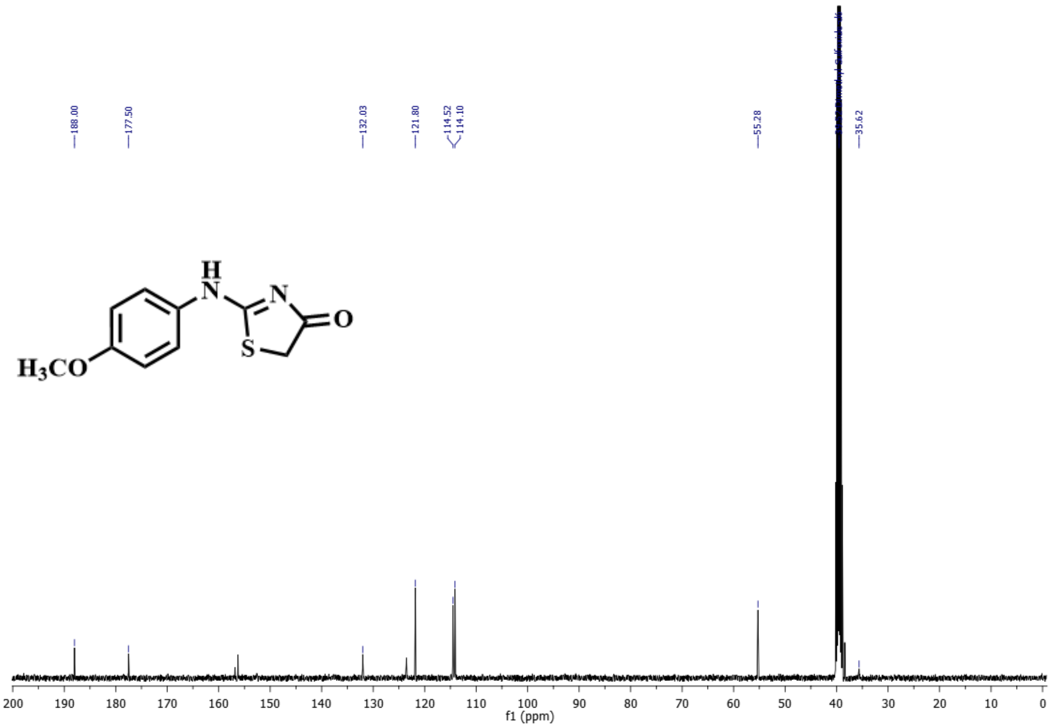

Figure S44: ^{13}C NMR of 2-((4-methoxyphenyl)amino)thiazol-4(5*H*)-one (3o)

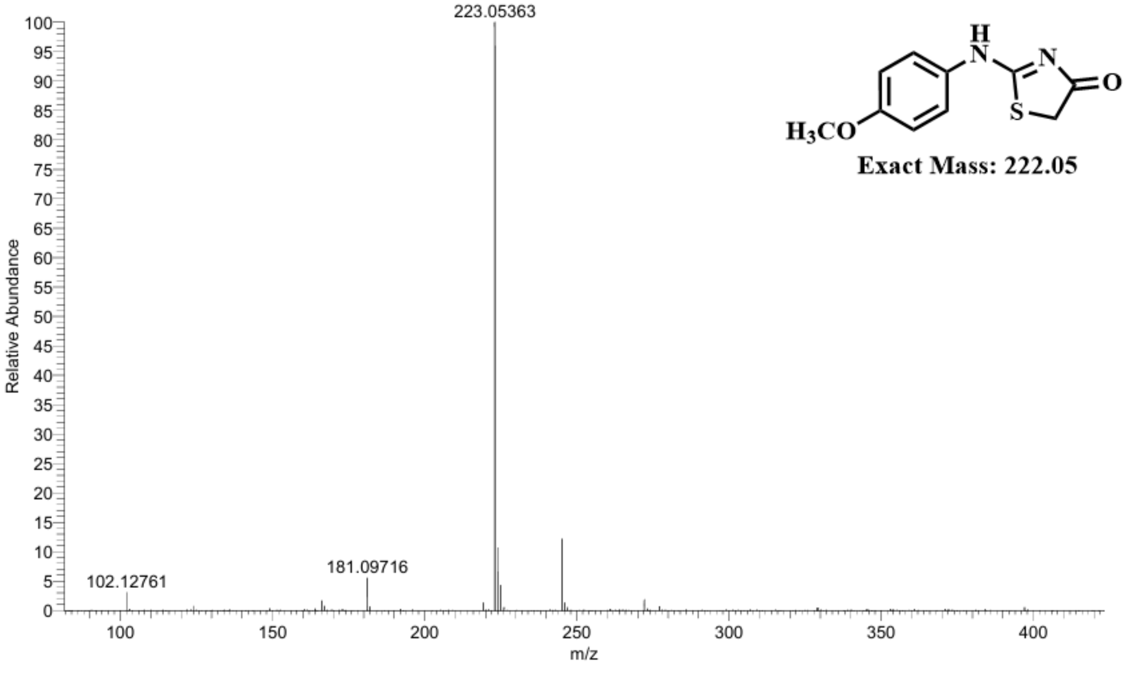

Figure S45: ESI-HRMS of 2-((4-methoxyphenyl)amino)thiazol-4(5*H*)-one (3o)

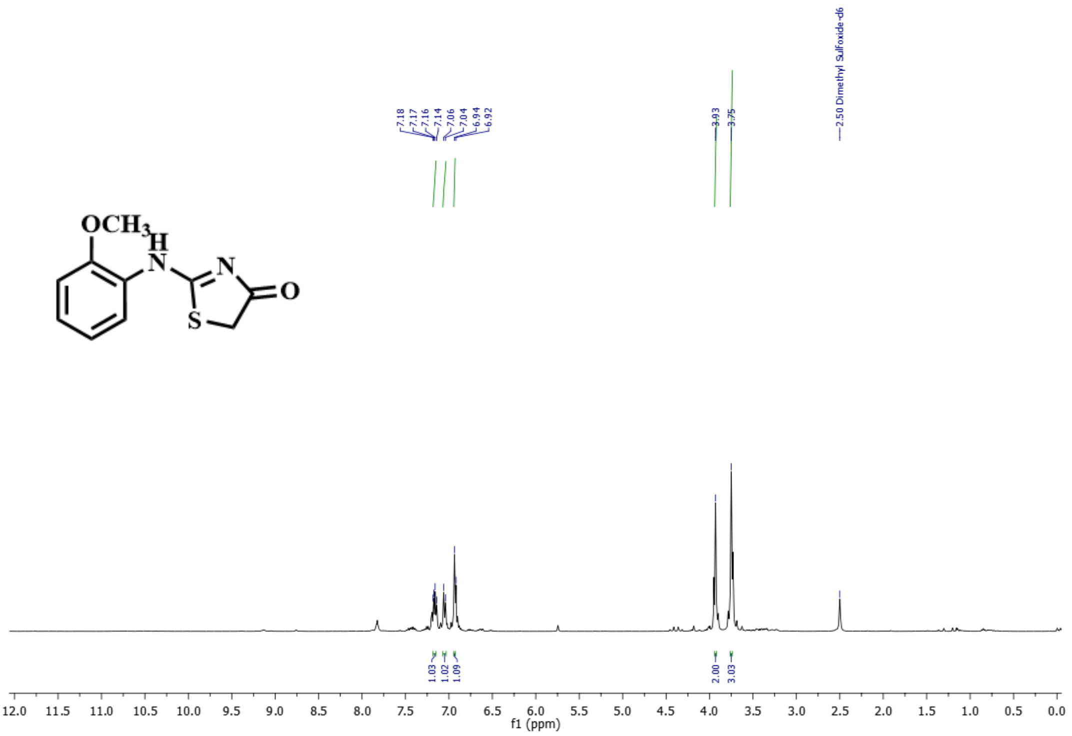

Figure S46: ^{1}H NMR of 2-((2-methoxyphenyl)amino)thiazol-4(5*H*)-one (3p)

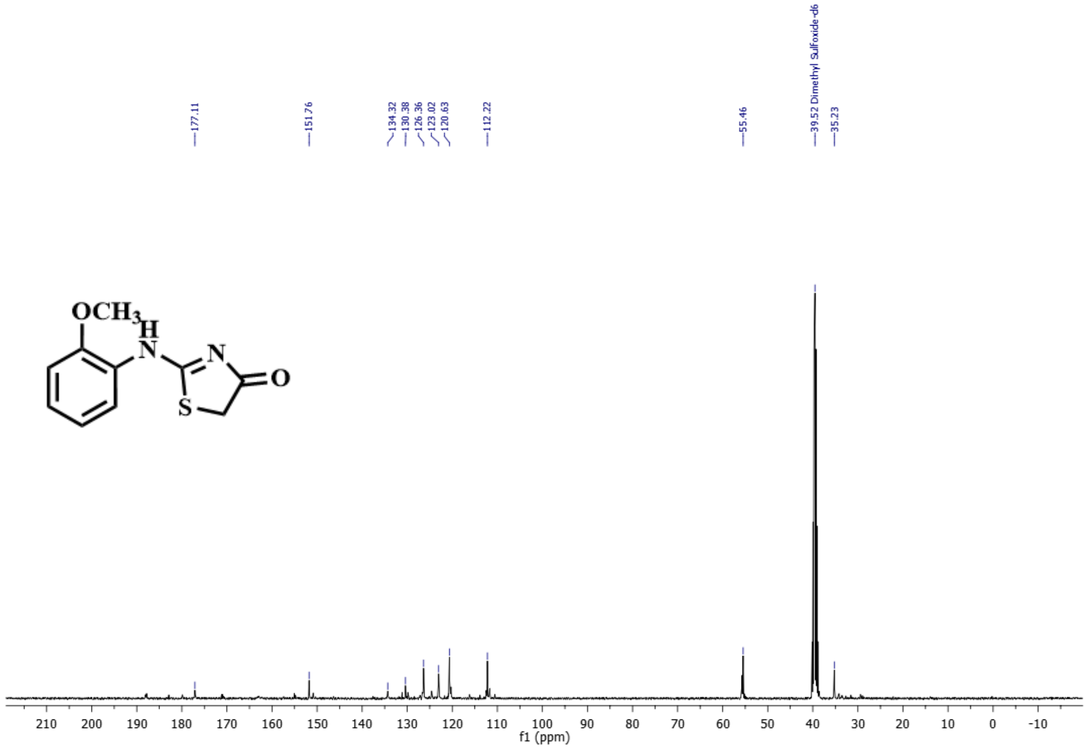

Figure S47: ^{13}C NMR of 2-((2-methoxyphenyl)amino)thiazol-4(5*H*)-one (3p)

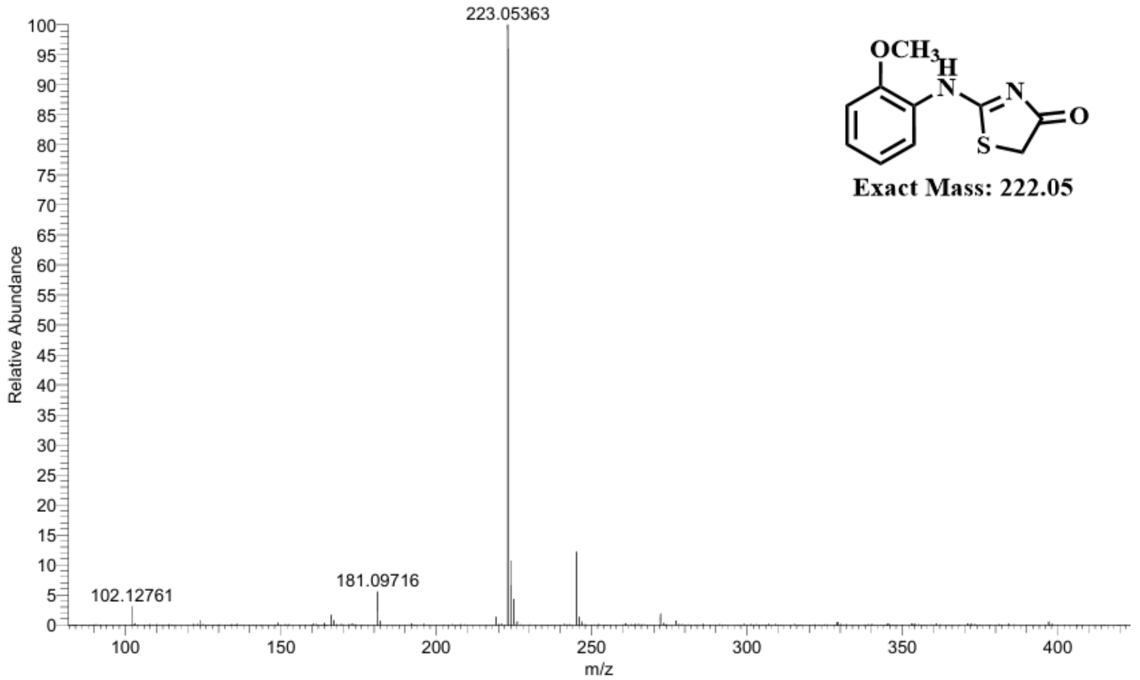

Figure S48: ESI-HRMS of 2-((2-methoxyphenyl)amino)thiazol-4(5*H*)-one (3p)

S2. HPLC chromatographic analysis of lead compounds

Methodology

The lead compounds (**3n, 3i,** and **3b**) were analyzed using an Agilent 1260 Infinity II HPLC system equipped with an Agilent Zorbax C18 column (150 × 4.6 mm, 5 µm). The mobile phase consisted of solvent A (Milli-Q water containing 0.1% formic acid) and solvent B (methanol containing 0.1% formic acid). The flow rate was maintained at 1.0 mL min^{-1}. Samples were monitored at 207 nm using a UV detector. The gradient was programmed from 20% to 70% solvent B over 5 min, followed by 70–99% B over 15 min, held at 99% B for 5 min, and re-equilibrated to the initial conditions for 5 min.

Results

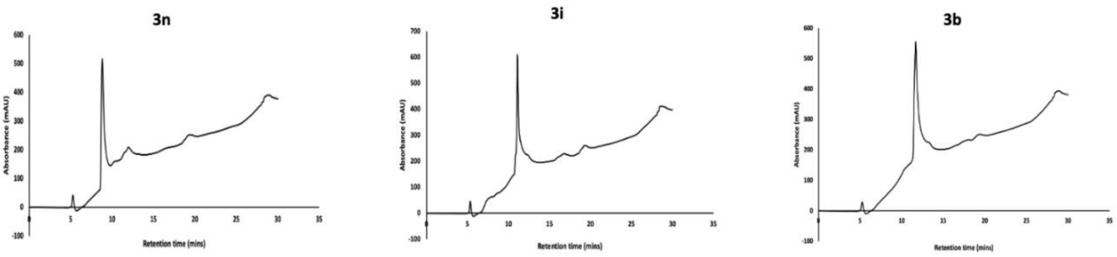

Figure S49. Representative RP-HPLC chromatograms of compounds **3n**, **3i**, and **3b** recorded using an Agilent 1260 Infinity II HPLC system equipped with a Zorbax C18 column under the chromatographic conditions described in the experimental procedure at 207 nm. The major peaks corresponding to the target thiazol derivatives were observed at retention times of 8.8 min, 11.0 min, and 11.6 min, respectively.

Integrated profiles

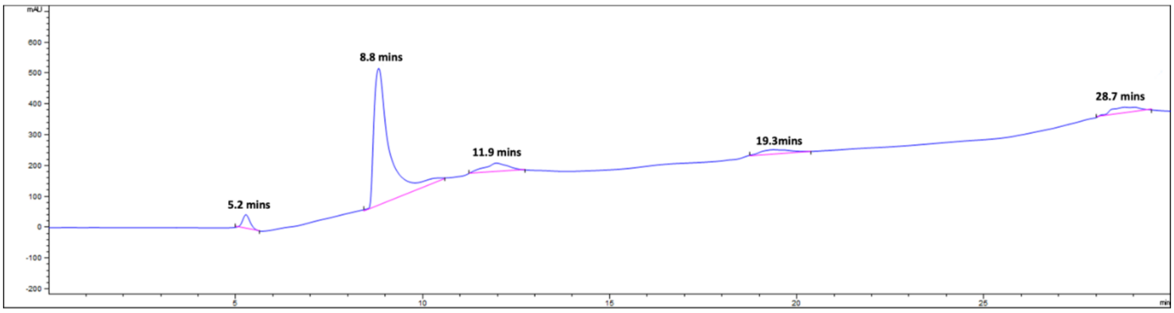

Figure S50. Integrated RP-HPLC chromatogram of **3n** recorded at 207 nm.

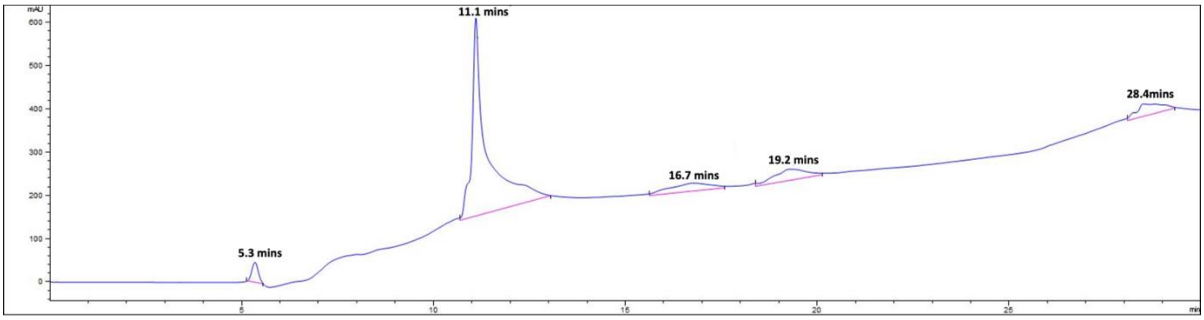

Figure S51. Integrated RP-HPLC chromatogram of **3i** recorded at 207 nm.

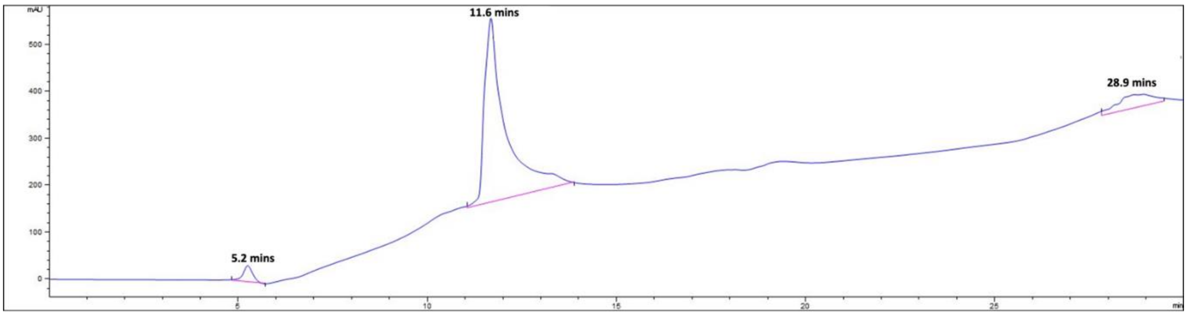

Figure S52. Integrated RP-HPLC chromatogram of **3b** recorded at 207 nm.

S3. Optimization of the microwave-assisted synthesis

Table S1. Optimization of reaction conditions for the target molecule

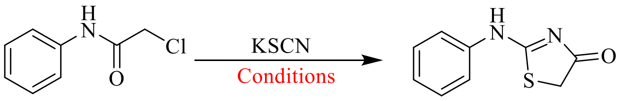

2a **3a**

Entry	Temp (°C)	Solvent	Time (min)	Yield (%)
1	100	Toluene	120	30
2	30	CH_2Cl_2	120	52
3	80	1,4 Dioxane	95	50

4	60	THF	100	55
5	80	Acetonitrile	45	78
6	100	DMF	40	82
7	100	DMSO	40	84
8	60	MeOH	35	85
9	80	EtOH	30	90
10	100	Water	30	91
11	100	EtOH-Water	25	93
12	MWI, 80	EtOH	10	90
13	MWI, 100	Water	5	92
14	MWI, 80	EtOH-Water	5	96
15	MWI, 50	EtOH-Water	5	82
16	MWI, RT	EtOH-Water	5	64

2-Chloro-*N*-phenyl acetamide (1 mmol), KSCN (1 mmol), EtOH-Water (1:1), Heating/ MWI (RT-100 ℃)

S4. Molecular docking, binding interaction analysis and target prediciton of 2-(arylamino)thiazol-4(5H)-one derivatives

Table S2. Compounds binding energy (in kcal/mol) after docking with AChE (PDB ID:4EY7). Lower binding energy values indicate stronger predicted binding affinity of the ligands toward the active site.

**Compounds**	**Binding Energy (kcal/mol)**	**Rank**
**3n**	-8.638	1
**3i**	-7.971	2
**3b**	-7.943	3
**3l**	-7.752	4
**3c**	-7.715	5
**3m**	-7.636	6
**3d**	-7.614	7
**3k**	-7.549	8
**3o**	-7.541	9
**3g**	-7.472	10
**3j**	-7.426	11
**3e**	-7.384	12
**3h**	-7.330	13
**3f**	-7.329	14
**3p**	-7.303	15
Donepezil	-11.765	Reference

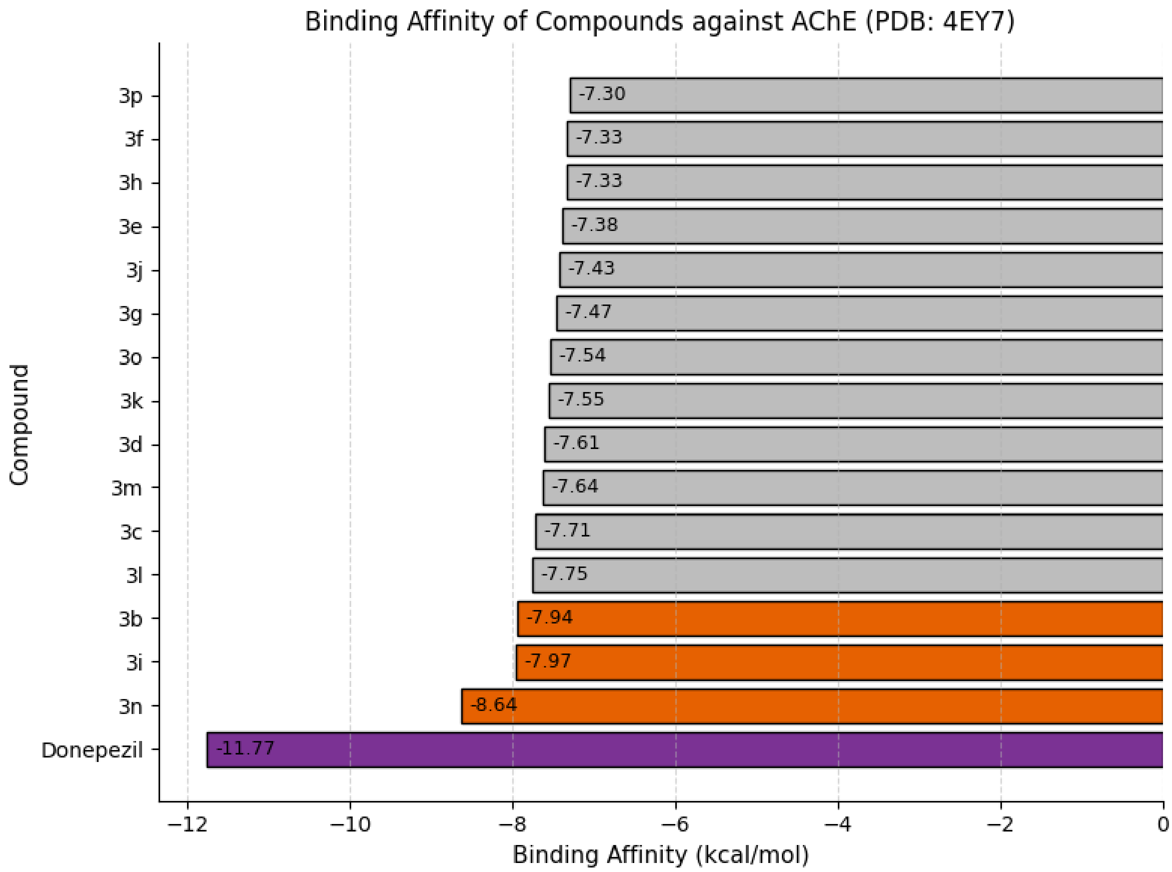

Figure S53. The graphical depiction of Binding affinity values of 2-(arylamino)thiazol-4(5H)-one derivatives. The top-performing compounds (**3n, 3i, and 3b**) are highlighted in orange, the reference ligand (Donepezil) in purple, and the remaining compounds in grey.

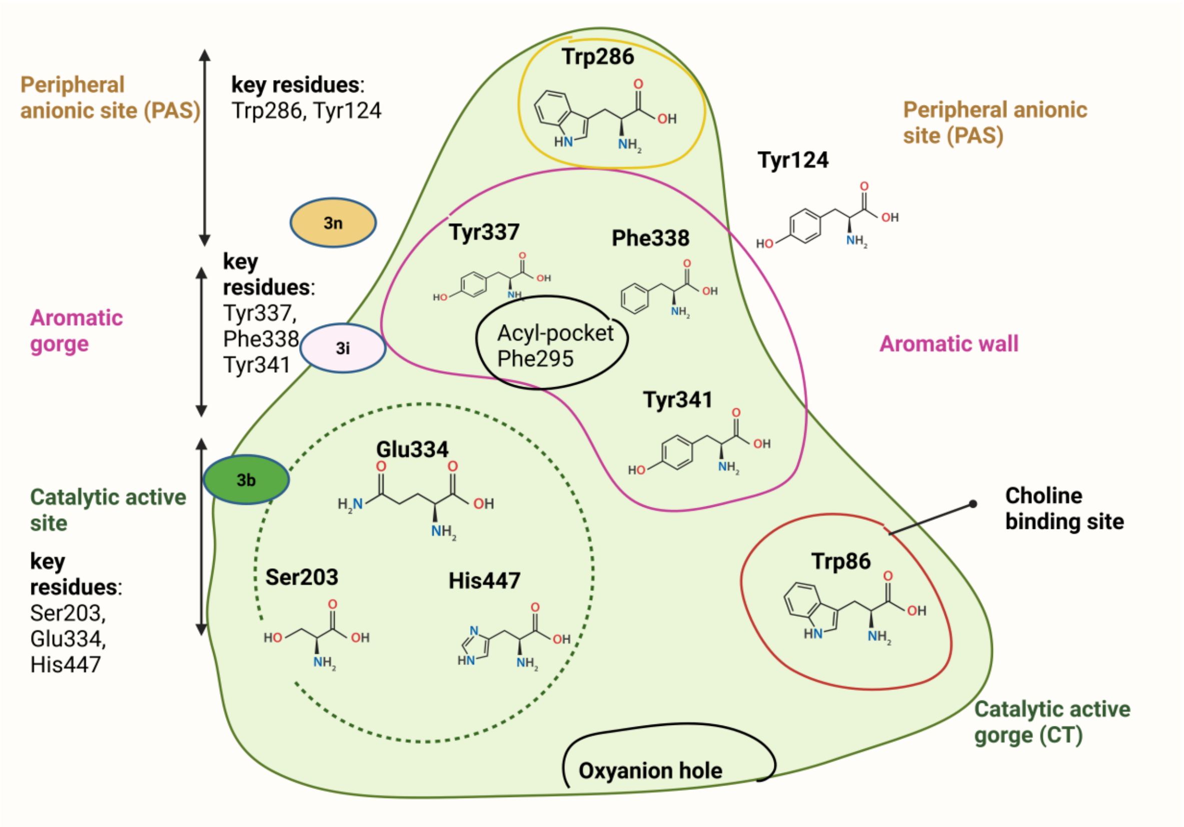

Figure S54. Schematic representation of the acetylcholinesterase (AChE) active-site gorge showing key functional regions, including the peripheral anionic site (PAS), aromatic gorge, catalytic active site (CAS), acyl pocket, and oxyanion hole. Important residues involved in ligand binding are highlighted, along with the approximate binding positions of compounds **3n, 3i,** and **3b**. Image was made using biorender, adapted from Peitzika *et al*., 2023 [42].

Table S3. Detailed protein–ligand interaction profiles of compounds **3n, 3i,** and **3b** with acetylcholinesterase (AChE; PDB ID: 4EY7), obtained using the Protein–Ligand Interaction Profiler (PLIP).

**Compound: 3n (Binding energy: −8.638 kcal/mol)**				
**Interaction Type**	**Residue**	**Amino Acid**	**Distance (Å)**	**Description**
Hydrophobic	286A	TRP	3.66–3.59	Aromatic contact within gorge
Hydrophobic	337A	TYR	3.69	π-associated hydrophobic interaction
Hydrophobic	338A	PHE	3.74	Stabilization via aromatic residue
Hydrophobic	341A	TYR	3.71–3.80	Multiple contacts with aromatic side chain
Hydrogen bond	124A	TYR	2.53 (H–A)	Backbone-mediated interaction
π–π stacking	341A	TYR	4.18	Parallel stacking interaction
**Compound: 3i (Binding energy: −7.971 kcal/mol)**				
**Interaction Type**	**Residue**	**Amino Acid**	**Distance (Å)**	**Description**
Hydrophobic	286A	TRP	3.40	Interaction with peripheral site
Hydrophobic	338A	PHE	3.65	Aromatic stabilization
Hydrophobic	341A	TYR	3.76	Key interaction in binding pocket
π–π stacking	337A	TYR	4.41	Aromatic stacking (parallel)

π–π stacking	341A	TYR	4.01	Additional aromatic stacking
**Compound: 3b (Binding energy: −7.943 kcal/mol)**				
Hydrophobic	124A	TYR	3.48	Peripheral hydrophobic contact
Hydrophobic	297A	PHE	3.72	Stabilizing interaction
Hydrophobic	337A	TYR	3.24	Strong Aromatic contact
Hydrophobic	338A	PHE	3.47-3.84	Multiple interactions
Hydrogen bond	120A	GLY	3.41	Backbone interaction
Hydrogen bond	133A	TYR	2.83	Side-chain mediated bond
Hydrogen bond	203A	SER	2.60	Strong polar interaction
Hydrogen bond	447A	HIS	2.86	Catalytic site interaction
π–π stacking	337A	TYR	5.28	T-shaped stacking
**Reference ligand: Donepezil / E20 (Binding energy: −11.765 kcal/mol)**				
Hydrophobic	86A	TRP	3.84	Aromatic pocket interaction
Hydrophobic	337A	TYR	3.66	Stabilizing aromatic contact
Hydrophobic	338A	PHE	3.77–3.83	Multiple interactions
Hydrophobic	341A	TYR	3.60–3.95	Dominant interaction region
Hydrogen bond	295A	PHE	1.99 (H–A)	Strong hydrogen bonding
Water bridge	341A	TYR	3.06	Solvent mediated stabilization
π–π stacking	86A	TRP	3.89	Parallel stacking
π–π stacking	286A	TRP	3.82	Aromatic cage interaction
π–π stacking	341A	TYR	4.23	Key stacking interaction

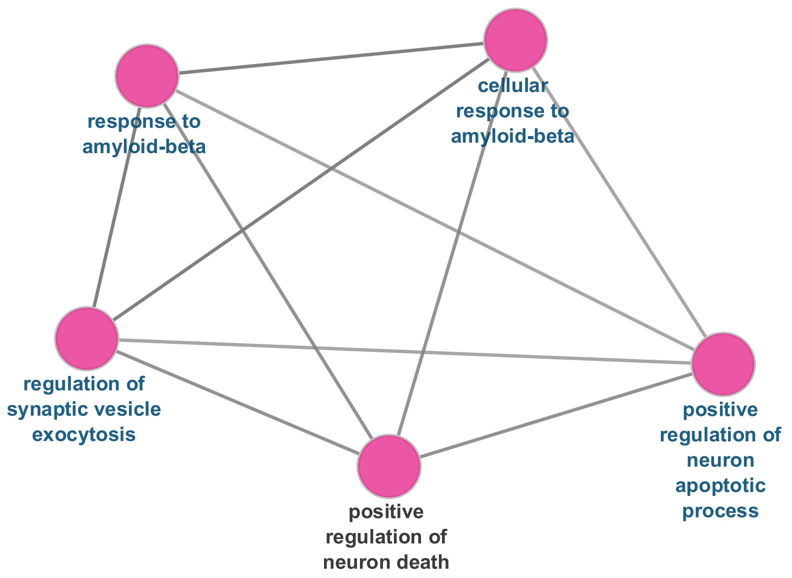

Figure S55. ClueGO network showing enriched biological processes associated with the overlapping targets. Gene Ontology enrichment analysis further indicates that these targets are involved in key processes, including amyloid-β response, neuronal apoptosis, and synaptic function.

S5. *In-vitro* studies

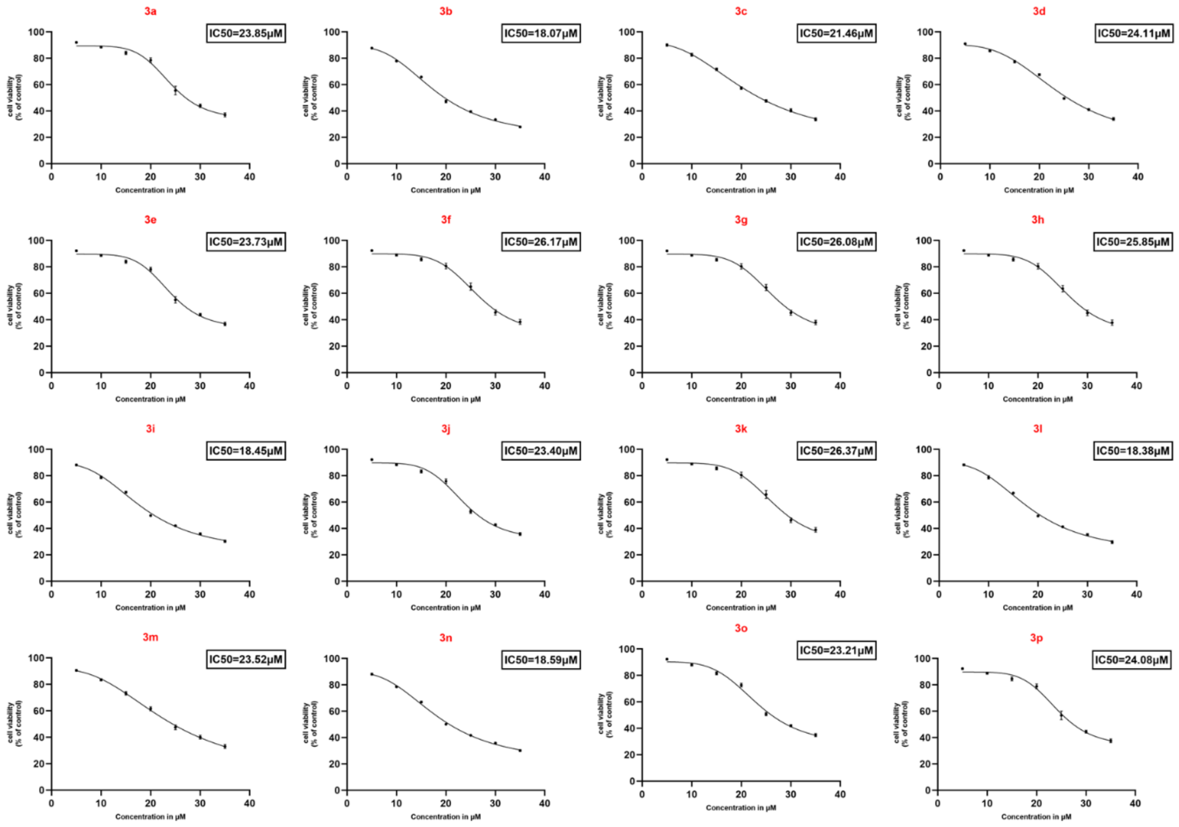

Figure S56. Dose–response curves with IC50 values for 2-(arylamino)thiazol-4(5H)-one derivatives Data are mean ± SEM (N = 3).

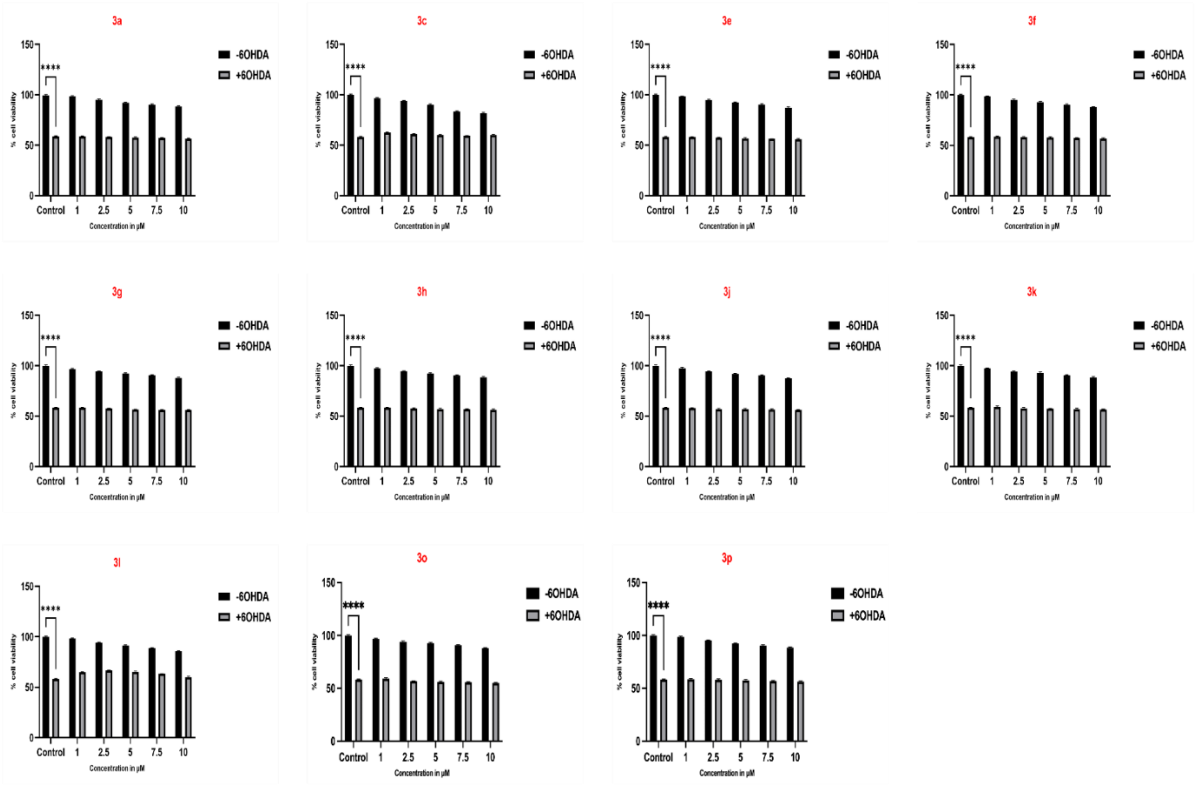

Figure S57. Neuroprotective activity of 2-(arylamino)thiazol-4(5H)-one derivatives on 6-OHDA-induced SH-SY5Y neuronal cells. Cell viability was assessed through the MTT assay ($N = 5$). Data are reported as the mean ± SEM and were statistically analyzed with one-way ANOVA. $*p \leq 0.05$ compared to the control group. $\#p \leq 0.05$ compared to the 6-OHDA-induced group.

Table S4. Summary of the structure–activity relationship (SAR) of the lead 2-(arylamino)thiazol-4(5H)-one derivatives

**Compound**	**Structure**	**Docking (kcal/mol)**	**AChE IC_{50} (μM)**	**ROS reduction**	***Drosophila* motor function**
**3n**	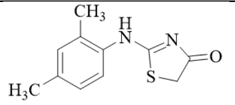	- 8.638	12.03 ± 0.17	Highest	Restored
**3i**	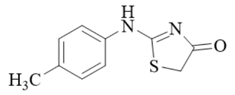	**-7.971**	22.32 ± 0.26	High	No
**3b**	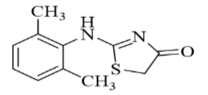	**-7.943**	29.51 ± 0.05	Moderate	No

S6. ***In-vivo*** **studies**

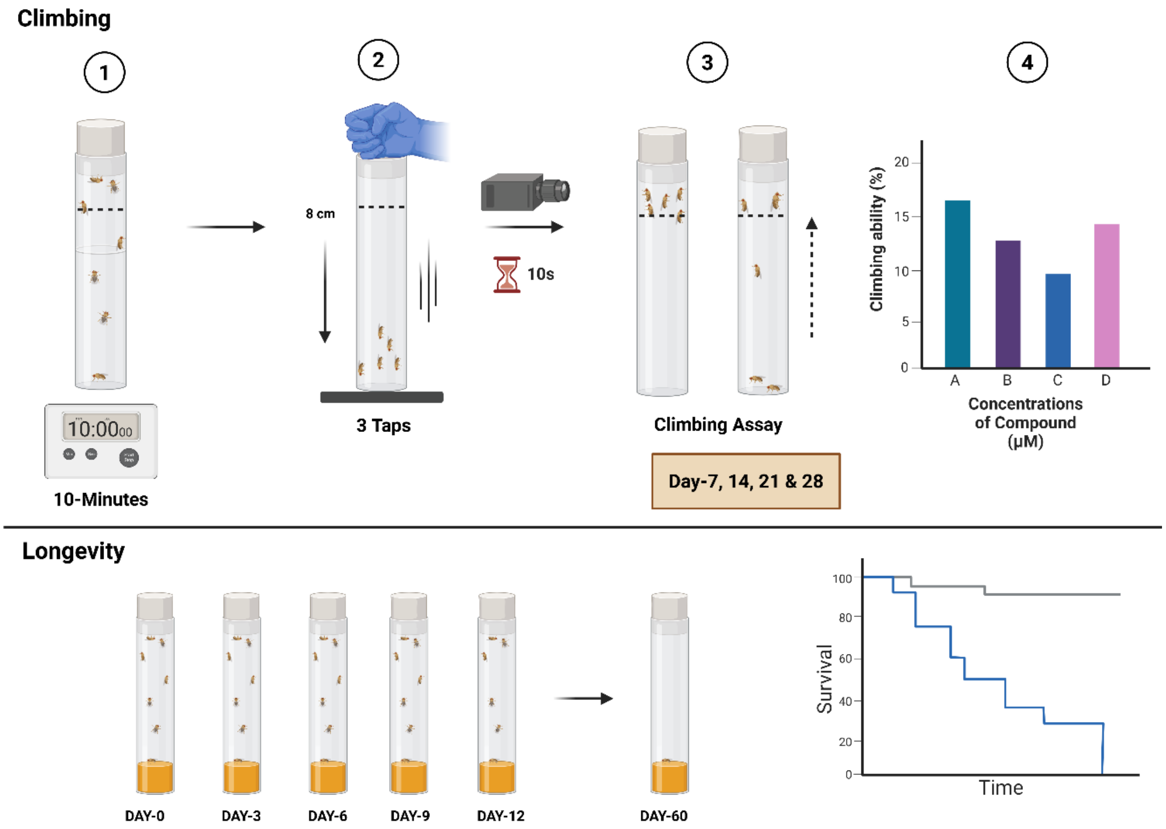

Figure S58. Schematic overview of the *Drosophila melanogaster* experimental design
